# Bispecific antibody engineered extracellular vesicles redirect T cells to prevent postoperative epidural fibrosis

**DOI:** 10.1038/s41467-026-74883-3

**Published:** 2026-06-27

**Authors:** Shanwei Ye, Qian Xu, Yong Xu, Hui Lu, Zhenfeng Liu, Jianfeng Guo, Yichuan Li, Wei Wang, Jun Ran, Xiaohua Zhu, Dongling Zhu, Wei Wu, Zechuan Yang, Shiqi Gu, Feng Li, Lugui Qiu, Ellen Puré, Vijay G. Bhoj, Liang Huang, Wei Xiong, Zheng Zhang

**Affiliations:** 1 Department of Orthopedics, Tongji Hospital, Tongji Medical College, Huazhong University of Science and Technology, Wuhan, Hubei China; 2 Department of Hematology, Tongji Hospital, Tongji Medical College, Huazhong University of Science and Technology, Wuhan, Hubei China; 3 State Key Laboratory of Experimental Hematology, National Clinical Research Center for Blood Diseases, Haihe Laboratory of Cell Ecosystem, Institute of Hematology & Blood Diseases Hospital, Chinese Academy of Medical Science & Peking Union Medical College, Tianjin, China; 4 Tianjin Institutes of Health Science, Tianjin, China; 5 Department of Pathology & Laboratory Medicine, University of Pennsylvania, Perelman School of Medicine, Philadelphia, PA USA; 6 Department of Orthopedics, The First Affiliated Hospital, Zhejiang University School of Medicine, Hangzhou, Zhejiang China; 7 Department of Nuclear Medicine, The First Affiliated Hospital, Zhejiang University School of Medicine, Hangzhou, Zhejiang China; 8 Department of Dermatology, Tongji Hospital, Tongji Medical College, Huazhong University of Science and Technology, Wuhan, Hubei China; 9 Department of Surgery, University of Pennsylvania, Perelman School of Medicine, Philadelphia, PA USA; 10 Department of Radiology, Tongji Hospital, Tongji Medical College, Huazhong University of Science and Technology, Wuhan, Hubei China; 11 Department of Nuclear Medicine, Tongji Hospital, Tongji Medical College, Huazhong University of Science and Technology, Wuhan, Hubei China; 12 Institute of Organ Transplantation, Tongji Hospital, Tongji Medical College, Huazhong University of Science and Technology, Wuhan, Hubei China; 13 Department of Biomedical Sciences, University of Pennsylvania, Philadelphia, PA USA

**Keywords:** Immunotherapy, Translational research, Biomaterials, Mechanisms of disease

## Abstract

Epidural fibrosis (EF) is a frequent and debilitating complication that impairs recovery following spinal surgery, yet effective targeted therapies are lacking. Here we observe enrichment of FAP⁺ fibroblasts at surgical sites in patients after laminectomy. To therapeutically target this subset, we develop bispecific antibody–decorated extracellular vesicles (BsAb EVs), which redirect endogenous T cells to eliminate FAP⁺ fibroblasts in situ. In a preclinical model, BsAb EVs selectively eliminate pathogenic fibroblasts, reduce fibrotic collagen accumulation, and prevent the development of postoperative epidural fibrosis without detectable systemic toxicity under the tested conditions. Single-cell RNA sequencing reveals that FAP⁺ fibroblasts represent a transcriptionally distinct subset from α-SMA⁺ myofibroblasts, characterized by enhanced extracellular matrix remodeling and TGF-β production. Together, these findings highlight a critical stromal subset in EF pathogenesis and position BsAb EVs as a promising immunotherapeutic strategy for targeting pathogenic stromal cells in fibrotic and tissue-remodeling disorders.

## Abstract (web summary)

Scar tissue after spine surgery can trap nerves and complicate revision operations. Here, authors implicate FAP-marked stromal cells in fibrotic remodeling and develop BsAb-engineered extracellular vesicles that redirect T-cells to reduce epidural fibrosis.

## Introduction

Postoperative epidural fibrosis (EF) is a distinctive and clinically significant form of fibrotic remodeling that frequently occurs after spinal surgery, with reported incidence ranging from 24 to 86% depending on the surgical type and assessment method^1,2^. Despite technically successful procedures, EF can still develop, forming dense adhesions that compress nerve roots and are widely regarded as a major contributor to failed back surgery syndrome, accounting for approximately 20–36% of the cases^1,3,4^. In revision cases, the presence of EF is observed in over 90% of patients, complicating dissection by obscuring anatomical planes, increasing the risk of dural or nerve injury, and prolonging operative time^5–7^. With spine surgery rates rising markedly across the world, the clinical burden of postoperative EF is escalating.

Current management strategies largely focus on physical barriers, such as grafts or biomaterials, to mechanically separate the dura from adjacent tissues^8–13^. However, these approaches merely provide transient mechanical separation without deeply eliminating the underlying pro-fibrotic responses. Moreover, they are frequently associated with complications such as neural compression, infection, or immune rejection caused by implanted materials^14^. Critically, mechanistic insights into EF pathogenesis remain scarce. The specific fibroblast subsets that drive adhesion formation, and their interactions with immune cells, have not been systematically defined. This gap hinders the development of targeted therapies that could prevent pathological wound remodeling without impairing normal healing.

Immunotherapeutic strategies, particularly chimeric antigen receptor (CAR) T cell therapy, are increasingly being explored beyond oncology, including in autoimmune and fibrotic diseases^15–21^. These approaches are characterized by high specificity and the ability to eliminate well-defined pathogenic cell populations, making them attractive options for treating non-malignant conditions involving refractory or relapsing immune-mediated pathology^22^. Nevertheless, the clinical translation of CAR-T therapy in benign disease contexts remains limited by logistical and safety barriers^23^.

In this study, we identify a marked enrichment of FAP-expressing fibroblasts in epidural fibrotic tissue following laminectomy, which are closely associated with ECM accumulation and adhesion formation. Further experiments reveal that FAP⁺ fibroblasts play a central pathogenic role in driving EF progression. To therapeutically eliminate these pathogenic fibroblasts, we engineer a bispecific antibody (BsAb) decorated extracellular vesicle (EV) platform targeting both FAP and CD3, building upon our previous work on EV-based immune redirection systems^24,25^. This strategy recruits endogenous T cells to eliminate FAP⁺ fibroblasts at the site of injury. In a preclinical immunocompetent EF mouse model, BsAb EVs significantly reduced fibrosis, preserved neural architecture, and reprogrammed the stromal immune interface without detectable systemic toxicity under the tested conditions. Collectively, these findings establish a proof of concept that FAP targeted BsAb engineered EVs can redirect endogenous T cells to selectively eliminate pathogenic fibroblasts and prevent the development of postoperative EF.

## Results

### Impact of recurrent surgery on postoperative adverse outcomes

To assess the clinical impact of prior surgery on subsequent lumbar revision procedures, we analyzed clinical data from 207 patients who underwent Transforaminal Lumbar Interbody Fusion (TLIF) at our center (Supplementary Table 1). Based on surgical history, patients were categorized into the primary surgery cohort and the revision surgery cohort. Among patients receiving single-level procedures, those in the revision cohort exhibited significantly longer operative times, greater intraoperative blood loss, prolonged drainage duration, increased total drainage volume, and extended postoperative hospitalization compared with the primary cohort (Supplementary Fig. 1A–E). In patients undergoing multi-level surgical procedures, only drainage duration, total drainage volume, and operative time showed significant intergroup differences (Supplementary Fig. 1F–J), likely reflecting the increased procedural complexity associated with extensive interventions. Moreover, the revision surgery cohort demonstrated a higher incidence of postoperative adverse events, including neurological deficits, poor wound healing, and dural tears (DT) (Supplementary Table 1). Notably, even in single-segment surgeries, the revision group exhibited a substantial risk of DT (9.09%), whereas no cases were observed in the primary surgery group.

**Fig. 1:**
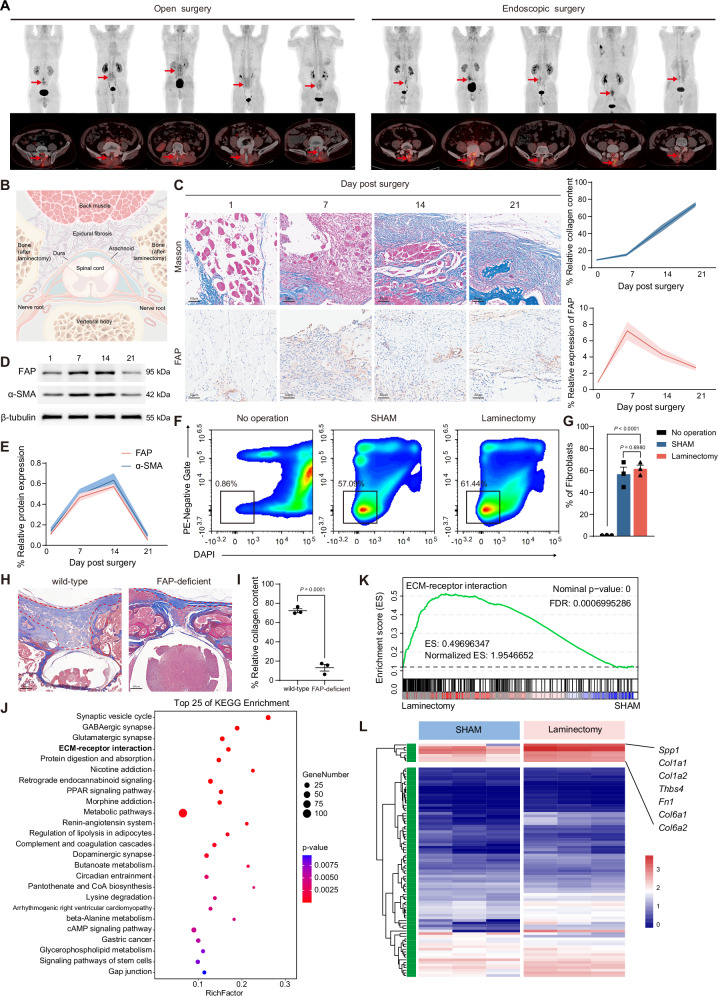
Post-laminectomy fibrotic tissue exhibits elevated FAP expression and ECM pathway activation. **A** Representative whole-body maximum intensity projection and axial fusion images of Gallium-68 (⁶⁸Ga)-labeled fibroblast activation protein inhibitor (FAPI) PET/CT acquired one week after surgery in ten participants with lumbar disc herniation (*n* = 10; 9 males, 1 female; mean age ± SD, 54.8 ± 17.4 years). Red arrows indicate elevated ⁶⁸Ga-FAPI uptake at the surgical site. **B** Schematic illustration of the epidural scar cross-section. **C** Time-course histological analysis of the laminectomy site by Masson’s trichrome staining and immunohistochemistry for FAP. Quantification of relative collagen content and the percentage of FAP-positive area per field was performed using ImageJ on three randomly selected high-power fields per mouse (*n* = 3 mice per time point, Scale bar, 50 μm). **D**, **E** Immunoblot analysis and corresponding densitometric quantification of FAP and α-smooth muscle actin (α-SMA) protein levels at the injury site over time (*n* = 3 mice per group). **F**, **G** Flow cytometry analysis and quantification of the percentage of fibroblasts in the injured tissue from naïve (no surgery), sham (removal of paraspinal soft tissue without lamina excision), and laminectomy groups at 7 days post-operation (*n* = 3 mice per group). Fibroblasts were identified as PE-lineage (CD31, CD45, Tie2, Ter119, EpCAM)-negative and DAPI-negative cells. **H**, **I** Masson’s trichrome staining and quantification of collagen-positive area in wild-type mice and FAP-deficient mice at 56 days post-laminectomy (*n* = 3 mice per group, Scale bar, 200 μm). **J** KEGG pathway enrichment analysis of bulk RNA sequencing data from SHAM versus laminectomy groups. **K** Gene Set Enrichment Analysis (GSEA) plot for the ECM-receptor interaction pathway. NES normalized enrichment score. **L** Heatmap of differentially expressed genes between the Sham and Laminectomy groups. Color scale represents Z-score of normalized read counts (blue, low expression; red, high expression). For all quantitative data, data are presented as mean ± SEM. Comparisons between two groups were performed using two-tailed unpaired Student’s *t*-test, while comparisons among three or more groups were performed using one-way ANOVA followed by Tukey’s multiple comparisons test. Source data are provided as a Source Data file.

Because postoperative scarring and EF are commonly considered contributors to the technical difficulty of revision lumbar surgery^1,4^, we next sought supportive clinical evidence of FAP-associated tissue remodeling at the surgical site. ^68^Ga-FAPI PET/CT imaging was performed one week postoperatively in ten patients who underwent lumbar decompression, including five who underwent open surgery and five who underwent endoscopic surgery. All patients demonstrated focal radiotracer uptake along the surgical trajectories, providing supportive evidence of FAP-associated stromal remodeling at the operative site (Fig. 1A). Although open surgery showed a trend toward higher tracer uptake compared to endoscopic surgery (SUVmax: 6.08 [3.85–8.31] vs. 5.26 [4.21–6.31]), the difference did not reach statistical significance (*P* = 0.3821). These clinical imaging data provided a rationale for further investigating FAP upregulation in postoperative fibrotic remodeling.

### FAP^+^ fibroblasts accumulate at postoperative sites

To further investigate the healing process of injured tissues following spine surgery, we established a murine laminectomy model. A schematic cross-section of EF-induced epidural scarring is presented in Fig. 1B. Progressive collagen deposition was observed to form a fibrous capsule encasing the spinal cord injury site and adhering to the dura mater (Fig. 1C). Immunohistochemical (IHC) staining, Western blot and qPCR showed a marked increase in FAP expression at postoperative day 7 and day 14, followed by a gradual decline at later time points, a pattern that closely paralleled α-SMA expression (Fig. 1C–E and Supplementary Figs. 1K, L and 2A, B). Following this, flow cytometry analyses of surgical tissue further demonstrated that both the laminectomy and sham groups exhibited significantly increased fibroblast accumulation compared to the non-operated control, with no notable difference between the two surgical groups (Fig. 1F, G). qPCR did not detect a corresponding increase in *Fap* transcript levels in non-surgical tissues (Supplementary Fig. 2C, D). To further investigate the functional role of FAP, we subjected both wild-type (WT) and FAP-deficient mice to the surgical model. On postoperative day 60, evaluation of collagen deposition at the lesion site revealed a marked attenuation of fibrosis in FAP-deficient mice compared with WT controls (Fig. 1H, I). These findings indicate that genetic loss of *Fap* attenuates postoperative EF, supporting a functional contribution of FAP to postoperative fibrotic remodeling in this model.

To investigate the distinct molecular features of tissue repair associated with spinal canal exposure, we performed transcriptomic profiling on scar tissues from the previously described No operation, SHAM, and Laminectomy groups. Differential expression analysis identified 4741 differentially expressed genes (DEGs) in the SHAM group compared with the No operation group, and 4922 DEGs in the Laminectomy group compared with the No operation group (Supplementary Fig. 1M, N). Then, a Venn diagram comparison of DEGs between the Laminectomy and SHAM groups relative to No operation controls was performed (Supplementary Fig. 1O). This analysis revealed 1046 genes uniquely differentially expressed in the Laminectomy group, with baseline expression maintained in SHAM-treated animals. KEGG pathway enrichment analysis showed that these laminectomy-specific genes were predominantly associated with extracellular matrix (ECM)-receptor interactions (Fig. 1J). Gene Set Enrichment Analysis further revealed significant enrichment of the ECM-receptor interaction pathway (Fig. 1K, NES = 1.95, *P* < 0.001). Notably, the Laminectomy group exhibited marked upregulation of key fibrosis-associated genes within the ECM network, including *Spp1*, *Col1a1*, *Col1a2*, and *Thbs4*, suggesting their critical roles in postsurgical fibrotic remodeling (Fig. 1L and Supplementary Fig. 1Q–S).

### SPP1⁺ macrophage-associated signaling contributes to FAP upregulation after spine surgery

**Fig. 2:**
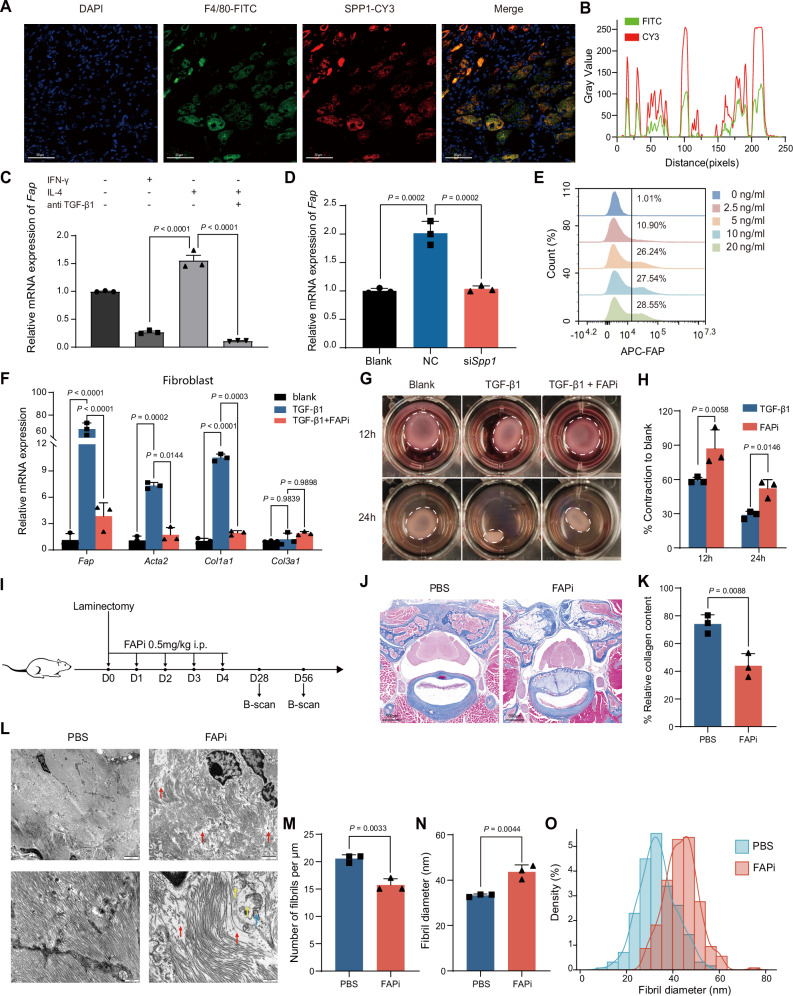
SPP1⁺ macrophage-associated signaling contributes to FAP⁺ fibroblast activation following laminectomy. **A**, **B** Representative immunofluorescence images and quantification of SPP1⁺ macrophage accumulation in laminectomy scar tissue at postoperative day 7 (*n* = 3 mice per group; scale bar, 50 μm). **C** *FAP* mRNA expression in fibroblasts exposed to conditioned medium (CM) from M0, M1, or M2 macrophages, with or without TGF-β1-neutralizing antibody (10 ng/mL, 48 h). Data are representative of three independent experiments. **D** *FAP* mRNA expression in fibroblasts treated with M2-CM following siRNA-mediated knockdown of *Spp1*. Data are from three independent experiments. **E** Flow cytometry analysis showing the proportion of FAP-positive fibroblasts after stimulation with increasing concentrations of TGF-β1 (0, 2.5, 5, 10, and 20 ng/mL, 48 h). **F** qPCR analysis of *Fap*, *Acta2*, *Col1a1*, and *Col3a1* mRNA levels in TGF-β1-stimulated fibroblasts treated with or without FAP inhibitor (FAPi, 10 μM, 48 h). Data are from three independent experiments. **G**, **H** Collagen gel contraction assay showing that FAPi attenuates TGF-β1-induced gel contraction. Data are from three independent experiments. **I** Schematic of the in vivo experimental design. Mice underwent laminectomy and received intraperitoneal injections of FAPi or PBS from day 0 to day 4 post-surgery. **J**, **K** Representative Masson’s trichrome staining of laminectomy scar tissue at day 56 and corresponding quantification of collagen deposition scores (*n* = 3 mice per group; scale bar, 500 μm). **L** Transmission electron microscopy of scar tissue at 56 days post-laminectomy. Red arrowheads indicate collagen fibers. Yellow arrowheads indicate mitochondrial rupture, and blue arrowheads indicate mild endoplasmic reticulum dilation. *n* = 3 mice per group. **M** Quantitative analysis of collagen fiber number in the scar tissue (*n* = 3 mice per group). **N**, **O** Quantitative analysis of collagen fiber diameter distribution (**N**) and mean fiber diameter (**O**) in the scar tissue (*n* = 3 mice per group). For all quantitative data, data are presented as mean ± SEM. Comparisons between two groups were performed using two-tailed unpaired Student’s *t*-test, while comparisons among three or more groups were performed using one-way ANOVA followed by Tukey’s multiple comparisons test. Source data are provided as a Source Data file.

Given the pivotal role of macrophages in early wound healing and the elevated SPP1 expression identified by RNA-seq, we sought to elucidate its involvement in postoperative scar formation. A significant accumulation of SPP1^+^ macrophages was observed in day 7 scar tissue (Fig. 2A, B). Meanwhile, TGF-β1—a key profibrotic factor secreted by M2 macrophages that promotes fibroblast activation—exhibited a progressive increase in serum concentration during the first postoperative week (Supplementary Fig. 3A). To further dissect macrophage–fibroblast interactions, in vitro assays were conducted using macrophages polarized toward M1 and M2 phenotypes via IFN-γ and IL-4 stimulation, respectively. M2 macrophages expressed markedly higher levels of SPP1 than M1 macrophages (Supplementary Fig. 3B). Conditioned medium from M2, but not M1 macrophages, robustly induced FAP expression in primary fibroblasts, an effect abolished by TGF-β1 neutralization (Fig. 2C). Moreover, siRNA-mediated knockdown of SPP1 in M2 macrophages reduced TGF-β1 secretion and attenuated their ability to promote FAP expression in fibroblasts (Fig. 2D and Supplementary Fig. 3C, D). Flow cytometric analysis confirmed that TGF-β1 induced a dose-dependent expansion of the FAP^+^ fibroblasts (Fig. 2E). Together, these findings suggest that M2 macrophages contribute to fibroblast activation and FAP upregulation through SPP1-TGF-β1 signaling.

Building on the genetic evidence from FAP-deficient mice, we next evaluated the small-molecule FAP inhibitor Ac-Gly-BoroPro (FAPi) to assess the therapeutic potential of pharmacological FAP blockade. In vitro, FAPi significantly suppressed TGF-β1-induced fibroblast activation, as evidenced by decreased *Acta2* and *Col1a1* expression (Fig. 2F) and impaired fibroblast contractility (Fig. 2G, H and Supplementary Fig. 3E, F). In vivo, laminectomy mice treated with FAPi exhibited a substantial reduction in epidural scar area and optical density by ultrasound imaging at late healing stages compared to PBS controls (Fig. 2I and Supplementary Fig. 3G–I). Masson’s trichrome staining further revealed that FAPi-treated scars displayed reduced and more loosely organized collagen deposition (Fig. 2J, K). Moreover, transmission electron microscopy (TEM) revealed marked ultrastructural alterations in fibroblasts after FAPi treatment. In the PBS group, fibroblasts exhibited densely organized collagen fibers and preserved mitochondrial structure, whereas fibroblasts from FAPi-treated mice showed disorganized collagen architecture, expanded interstitial spaces, and mitochondrial membrane disruption, accompanied by mild endoplasmic reticulum dilation (Fig. 2L). Quantitative analysis further confirmed reduced collagen fiber density and increased fiber diameter following FAPi treatment (Fig. 2M–O). Notably, mice in the FAPi-treated group exhibited transient body weight loss and compromised survival compared with control animals (Supplementary Fig. 3J, K). These observations raise potential safety concerns regarding FAPi administration. Taken together, these findings support FAP as a therapeutic target in postoperative fibrosis and highlight the need for more precise targeting strategies.

### Design, generation, and characterization of the FAP × CD3 BsAb EV platform

**Fig. 3:**
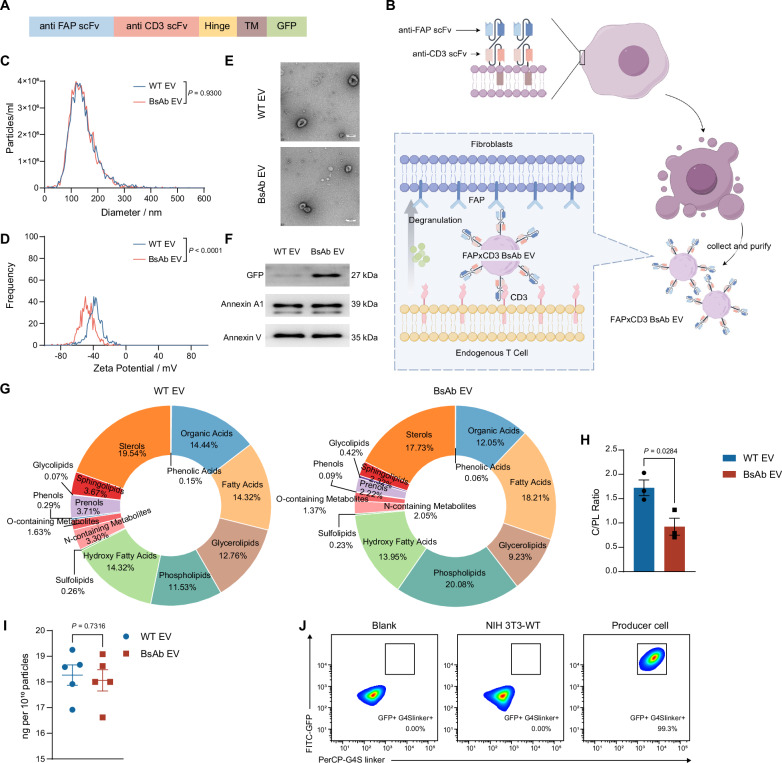
Construction and validation of FAP × CD3 BsAb EV platform. **A** Schematic representation of the FAP × CD3 bispecific antibody (BsAb) structure. **B** Design and proposed working principle of FAP × CD3 BsAb engineered extracellular vesicle (EV). This schematic was created using Figdraw (https://www.figdraw.com) with publication license No. WTWTY4c98c. **C–F** Morphological and physical characterization of FAP × CD3 BsAb EV. Nanoparticle tracking analysis revealed that both WT and BsAb EV preparations exhibited a predominant particle size distribution ranging from 100 to 200 nm in diameter, with no significant difference in size or concentration between the two groups (**C**). Zeta potential measurements confirmed surface charge stability, with a mean zeta potential of approximately −40 mV for WT EVs and −53 mV for BsAb EVs (**D**). Representative transmission electron microscopy images of negatively stained EVs displayed the characteristic cup-shaped vesicle morphology with clearly discernible phospholipid bilayer structures (scale bar, 100 nm); no morphological differences were observed between WT and BsAb EVs (**E**). Immunoblot analysis further validated the presence of GFP, Annexin A1, and Annexin V in the purified EV preparations (*n* = 3 biologically independent samples per group). **G** Pie chart depicting the percentage distribution of major lipid classes in BsAb EV, as determined by untargeted lipidomic profiling. **H** Cholesterol-to-phospholipid molar ratio in WT EVs and BsAb EV, quantified by fluorometric assay (*n* = 3 biologically independent samples per group). **I** Total RNA content in WT EVs versus BsAb EV. Data are from three independent experiments. Total RNA content in WT EVs versus BsAb EV, measured by Qubit RNA HS assay. *n* = 5 biological replicates per group. **J** Flow cytometry analysis of BsAb surface expression on parental producer cells and NIH 3T3-wt cells. Cells were stained with PerCP-conjugated anti-G4S linker antibody. Representative dot plots from one of three independent experiments are shown. For all quantitative data, data are presented as mean ± SEM. Comparisons between two groups were performed using two-tailed unpaired Student’s *t*-test or one-way ANOVA with Tukey’s multiple-comparisons test. Source data are provided as a Source Data file.

To eliminate FAP^+^ fibroblasts, we then developed the BsAb-engineered EV platform. The membrane-anchored FAP × CD3 BsAb was generated by tandem linkage of anti-FAP and anti-CD3 single-chain variable fragments (scFvs), followed by fusion to the CD8 hinge and transmembrane domains to ensure surface display on EV. A GFP tag was incorporated into the intracellular region for quantification (Fig. 3A). The construct was introduced into NIH/3T3 cells via lentiviral transduction, and cells with high BsAb expression were enriched by FACS sorting (Supplementary Fig. 4A). BsAb-modified EVs were subsequently collected and purified (Fig. 3B). BsAb EVs retained typical EV morphology and displayed greater stability than WT EVs, as indicated by a lower zeta potential (Fig. 3C–E).

Western blot analysis confirmed the presence of Annexin V, Annexin A1, TSG101, and CD63 in the EV preparations (Fig. 3F and Supplementary Fig. 4C). In addition, BsAb EVs were specifically positive for GFP, whereas Calnexin was undetectable in both EV preparations, supporting successful incorporation of the engineered construct and minimal contamination from cellular components. Nanoparticle tracking analysis (NTA) of independent batches confirmed comparable particle concentrations across preparations (Supplementary Fig. 4D). Lipidomic profiling further revealed an increased relative abundance of fatty acids and phospholipids, accompanied by a reduction in glycerolipids, in BsAb EVs compared with WT EVs, while other lipid classes remained largely unchanged (Fig. 3G and Supplementary Fig. 4E). Consistent with these changes, the cholesterol-to-phospholipid ratio was reduced in BsAb EVs (Fig. 3H). Total RNA content was comparable between WT EVs and BsAb EVs (Fig. 3I).

Finally, flow cytometric surface staining of the BsAb under non-permeabilizing conditions showed high concordance with the GFP signal, supporting efficient cell-surface display of the BsAb construct (Fig. 3J). Based on GFP fluorescence normalized to NTA-derived particle counts, we estimated an average construct loading of approximately 1.51 × 10^4^ GFP-tagged BsAb molecules per EV particle across three independent batches (Supplementary Fig. 4B).

### FAP × CD3 BsAb EVs redirect T cells to eliminate FAP-positive target cells in vitro and in vivo

**Fig. 4:**
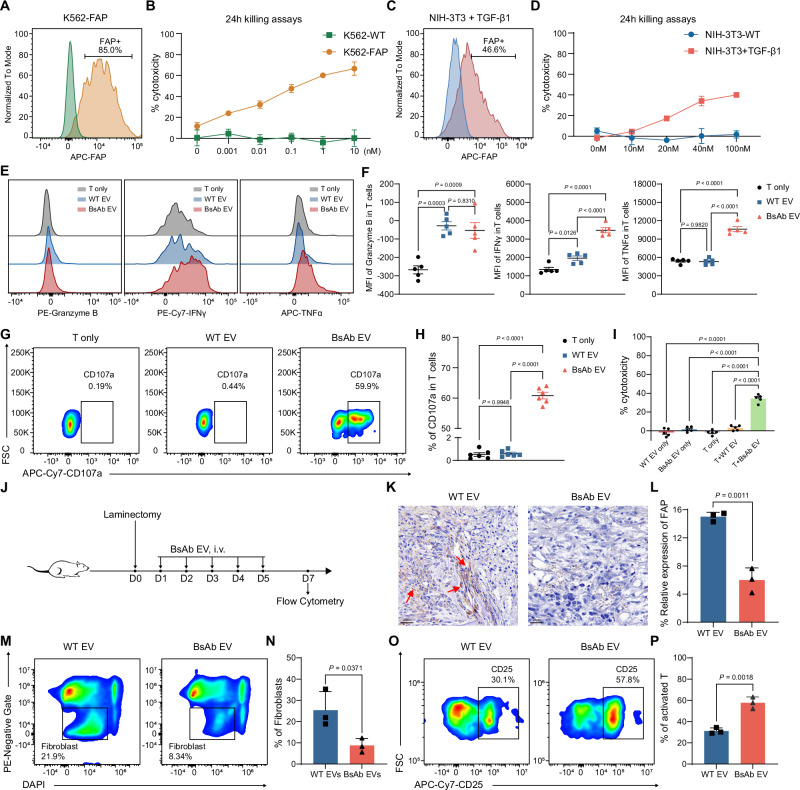
FAP × CD3 BsAb EVs redirect T cell cytotoxicity against FAP-positive target cells in vitro and in vivo. **A–D** Functional validation of FAP × CD3 BsAb EVs using FAP-overexpressing cell lines. **A** Representative overlaid flow cytometry histograms confirming FAP expression on K562-FAP cells compared with K562-wt cells. **B** Representative cytotoxicity assay showing specific lysis of K562-FAP and K562-WT target cells at an effector-to-target (E:T) ratio of 10:1. Similar results were observed across five independent experiments. **C** Representative overlaid flow cytometry histograms showing FAP expression on NIH 3T3 cells following stimulation with TGF-β1 (10 ng/mL, 48 h) versus untreated NIH 3T3-wt cells. **D** Cytotoxicity assay measuring specific lysis of TGF-β1-induced NIH 3T3 cells and NIH 3T3-wt cells at an E:T ratio of 10:1. Data are presented as mean ± SEM from three independent experiments. **E**, **F** Representative overlaid flow cytometry histograms (**E**) and quantification (**F**) of Granzyme B, IFN-γ, and TNF-α expression in CD3⁺CD8⁺ T cells following 24 h co-culture with BsAb EVs and FAP-positive target cells. *n* = 5 per group. **G**, **H** Representative overlaid density plots (**G**) and quantification (**H**) of CD107a expression in CD3⁺CD8⁺ T cells following 24 h co-culture with BsAb EVs and K562-FAP target cells. *n* = 6 per group. **I** Cytotoxicity against K562-FAP cells at an E:T ratio of 1:1 under the indicated treatment conditions. *n* = 5 per group. **J** The schematic diagram of the in vivo experiment. **K**, **L** Representative immunohistochemistry images and quantification of laminectomy scar tissue one week after surgery (*n* = 3 mice per group; scale bar, 25 μm). **M–P** Flow cytometric analysis of fibroblast and activated T cell populations in the scar tissue. Fibroblasts were identified as PE-lineage (CD31, CD45, Tie2, Ter119, EpCAM)-negative and DAPI-negative cells. Activated T cells were identified as CD3⁺CD25⁺ cells (*n* = 3 mice per group). For all quantitative data, data are presented as mean ± SEM. Comparisons between two groups were performed using two-tailed unpaired Student’s *t*-test, while comparisons among three or more groups were performed using one-way ANOVA followed by Tukey’s multiple comparisons test. Source data are provided as a Source Data file.

To assess functionality, FAP-overexpressing K562 cells and TGF‑β1‑treated NIH/3T3 cells were employed as target cells. A luciferase‑based cytotoxicity assay revealed dose‑dependent, T‑cell‑mediated killing in response to BsAb EV treatment (Fig. 4A–D). T cells were then co‑cultured either with FAP‑positive target cells in the presence of BsAb EVs or with BsAb EVs alone. In the presence of FAP‑positive target cells, BsAb EVs robustly activated T cells, as evidenced by increased degranulation and enhanced production of Granzyme B, TNF‑α, and IFN‑γ (Fig. 4E–H). In contrast, BsAb EVs alone induced only modest T-cell activation compared with that observed in the presence of FAP-positive target cells (Supplementary Fig. 5A–D). Consistent with this, substantial target-cell killing was observed only when T cells were present together with BsAb EVs (Fig. 4I). In addition, although BsAb EVs underwent progressive time-dependent loss during in vitro incubation, they retained detectable cytotoxic activity in both PBS and mouse serum for up to 48 h (Supplementary Fig. 5E–G).

To evaluate the in vivo targeting capability of the BsAb EV platform, we administered DIR-labeled WT EVs and BsAb EVs to mice following laminectomy. Compared with WT EV, BsAb EVs showed markedly greater accumulation at the laminectomy site as confirmed by quantitative fluorescence analysis (Supplementary Fig. 6A–D). This enrichment was absent in FAP-deficient mice (Supplementary Fig. 6E, F), indicating that local retention of BsAb EVs is FAP dependent.

We next examined whether BsAb EV treatment could deplete FAP⁺ fibroblasts in vivo. Daily tail vein administration of 40 nmol BsAb EVs for five days reduced the abundance of FAP⁺ fibroblasts at the surgical site (Fig. 4J–L). Flow cytometric analysis further showed a reduction in fibroblast populations accompanied by an increase in CD25⁺ activated T cells following treatment (Fig. 4M–P). Dose-dependent effects on fibroblast depletion and T-cell activation were observed across the tested dose range (Supplementary Fig. 7A–N). Consistent with local immune engagement, wound sections from BsAb EV-treated mice showed increased CD3⁺ T-cell infiltration and colocalization of CD3 and FAP signals (Supplementary Fig. 8A–C). Moreover, depletion of CD3⁺ T cells abolished the BsAb EV-mediated reduction in FAP⁺ fibroblasts (Supplementary Fig. 8D–I), supporting a T-cell-dependent mechanism in vivo. Together, these findings establish FAP × CD3 BsAb EVs as a functional and stable platform for the selective depletion of FAP⁺ fibroblasts.

### Single-cell analysis reveals selective depletion of profibrotic fibroblasts

**Fig. 5:**
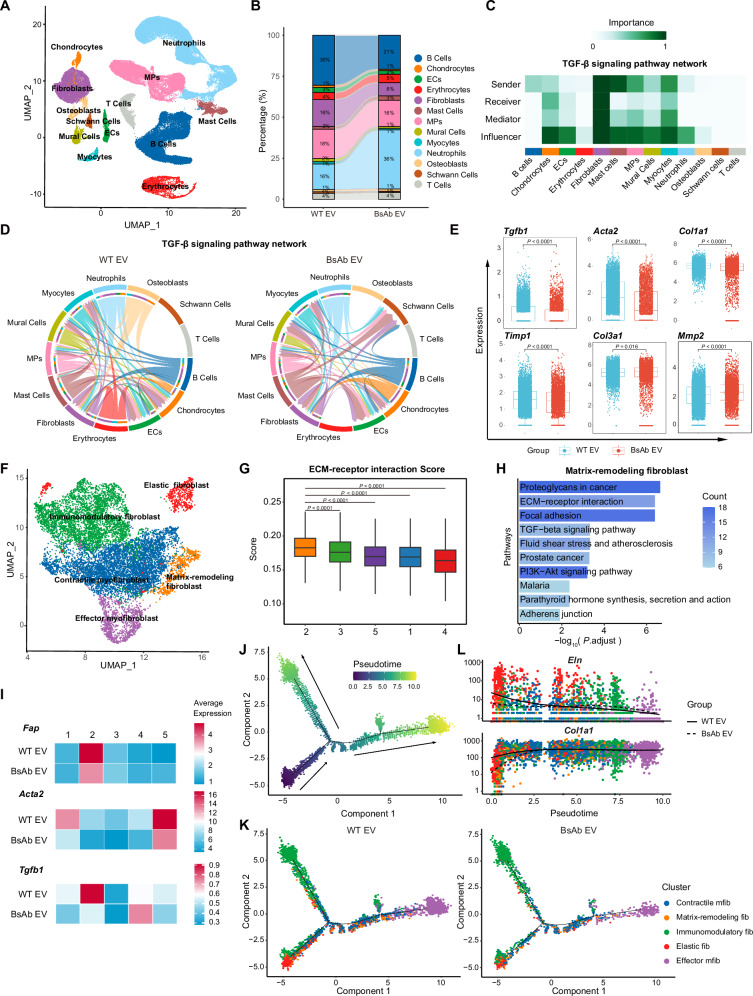
Single-cell RNA sequencing reveals BsAb EV-induced remodeling of the cellular architecture in postoperative fibrosis. **A** UMAP plot showing diverse cell populations within fibrotic lesions at postoperative day 7. scRNA-seq was performed on scar tissues from WT EV-treated and BsAb EV-treated mice (*n* = 3 mice per group). **B** Proportions of each cell population in fibrotic lesions treated with WT EVs or BsAb EV. **C** Heatmap showing the relative contribution of each cell population to the TGF-β signaling network based on four computed centrality measures. Color scale ranges from white (low) to dark green (high). **D** Inferred TGF-β signaling networks in WT EV- and BsAb EV-treated groups. Colored circle segments indicate 13 major cell types, and edge widths represent communication strength. **E** Expression levels of representative fibrosis-related genes in WT EV- and BsAb EV-treated groups. Box limits indicate interquartile range (IQR; 25th to 75th percentile); center lines denote median; whiskers extend to minima and maxima within 1.5 × IQR (WT EV, *n* = 46109 cells from 3 biological replicates; BsAb EV, *n* = 47330 cells from 3 biological replicates). **F** UMAP identifying five transcriptionally distinct fibroblast subsets. **G** ECM-receptor interaction signature scores across fibroblast subsets. Box limits indicate IQR; whiskers extend to minima and maxima within 1.5 × IQR (Fibroblasts-1, *n* = 5106 cells; Fibroblasts-2, *n* = 598 cells; Fibroblasts-3, *n* = 3967 cells; Fibroblasts-4, *n* = 583 cells; Fibroblasts-5, *n* = 1081 cells). **H** Top ten enriched signaling pathways associated with matrix-remodeling fibroblasts. **I** Heatmap showing expression of key fibroblast markers including *Fap*, *Acta2*, and *Tgfb1*. Color scale represents Z-score of normalized expression (blue, low; red, high). **J–L** Monocle2 trajectory analysis showing pseudotime ordering (**J**), cell type annotation (**K**), and dynamic expression of Eln and Col1a1 along fibroblast trajectories (**L**). Data are mean ± SEM. Two-group comparisons were performed using two-tailed unpaired Student’s *t*-test; multiple-group comparisons were performed using one-way ANOVA followed by Tukey’s test. Source data are provided as a Source Data file.

To investigate how BsAb EV therapy alters the cellular landscape of the postoperative site, we performed single-cell RNA sequencing (scRNA-seq) of epidural tissue harvested one week after laminectomy from mice treated with BsAb EVs or WT EVs (Fig. 4J). Unbiased clustering identified 13 transcriptionally distinct cell populations (Fig. 5A and Supplementary Fig. 9A, B). Cell type proportion analysis revealed a significant reduction in fibroblasts in the BsAb EVs group, whereas total T cells and mononuclear phagocytes (MPs) remained largely unchanged. Interestingly, neutrophil abundance was elevated (Fig. 5B), reflecting selective remodeling of the local cellular composition.

Then, we conducted network centrality and ligand-receptor interaction analyses, focusing on the TGF-β signaling axis. Fibroblasts emerged as primary hubs of TGF-β signaling, suggesting their pivotal role in initiating and sustaining fibrosis, with MPs acting as auxiliary senders and influencers (Fig. 5C). Both TGF-β–centered and overall cell–cell communication networks were markedly attenuated following BsAb EV treatment (Fig. 5D and Supplementary Fig. 9C–E), indicating effective disruption of profibrotic signaling circuits. Transcriptomic analysis of fibroblasts revealed broad transcriptional reprogramming: profibrotic genes, including *Tgfb1*, *Acta2*, *Col1a1*, and *Timp1* were downregulated, while genes associated with matrix remodeling, including *Mmp2* and *Col3a1*, were upregulated (Fig. 5E and Supplementary Fig. 9F), suggesting a shift toward a remodeling-prone fibroblast state.

To further explore the internal heterogeneity within scar fibroblasts, subclustering analysis identified five transcriptionally distinct fibroblast subsets (Clusters 1–5; Fig. 5F–H and Supplementary Fig. 10A–F). Among these, Cluster 2 (Matrix-remodeling fibroblasts) exhibited strong enrichment for ECM-receptor interaction, focal adhesion, and TGF-β signaling pathways according to KEGG analysis, along with the highest ECM module scores (Fig. 5G, H). Notably, *Fap* and *Tgfb1* expression was preferentially enriched in Cluster 2 and markedly diminished upon BsAb EV treatment (Fig. 5I and Supplementary Fig. 10G). Clusters 5, characterized by high *Acta2* (α-SMA) expression, reflected myofibroblast-like phenotype that was clearly distinct from Cluster 2 (Fig. 5I and Supplementary Fig. 10H–I). Although these α-SMA⁺ subsets expressed minimal FAP, *Acta2* levels were also significantly suppressed in response to BsAb EV treatment, suggesting that clearance of FAP⁺, TGF-β-producing fibroblasts indirectly attenuates myofibroblast activation through non-cell-autonomous mechanisms.

Pseudotime trajectory analysis revealed a continuum of fibroblast state transitions. Contractile myofibroblasts, matrix-remodeling fibroblasts, and elastic fibroblasts (Clusters 1, 2, and 4) were mainly distributed across the mid and early-to-mid stages of the trajectory, whereas immunomodulatory fibroblasts (Cluster 3) appeared both at the origin and at later stages. Effector myofibroblasts (Cluster 5) occupied a distal branch, forming a bifurcated structure together with Cluster 3 (Fig. 5J–L and Supplementary Fig. 11A, B). Gene expression dynamics along pseudotime further revealed that hallmark fibrogenic genes such as *Eln* and *Col1a1* were notably reduced at early stages following BsAb EV treatment (Fig. 5L), suggesting early disruption of fibrotic gene activation. Collectively, these data suggest that BsAb EV treatment selectively depletes a profibrotic FAP⁺ fibroblast subset and perturbs TGF-β–driven differentiation trajectories.

### BsAb EV treatment remodels the local immune microenvironment

**Fig. 6:**
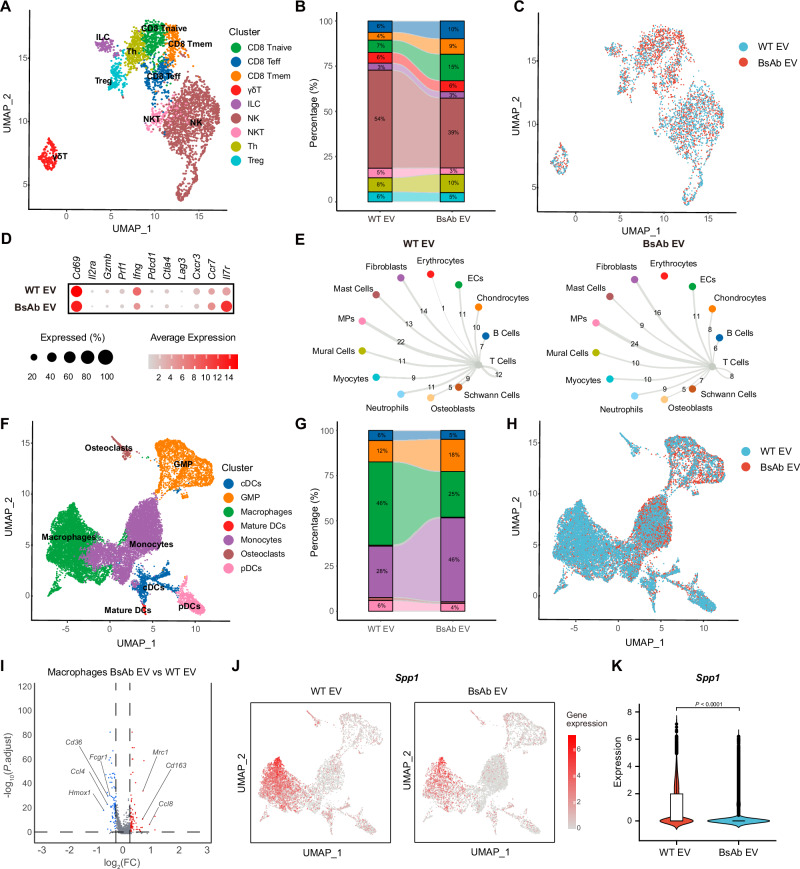
Characterization of T/NK cells and mononuclear phagocytes (MPs) in postoperative fibrotic lesions following treatment with WT EVs or BsAb EV. **A** UMAP plot depicting T and NK cells across fibrotic lesions at postoperative day 7. scRNA-seq was performed on scar tissues from WT EV-treated and BsAb EV-treated mice (*n* = 3 mice per group). **B** Proportions of T and NK cell subtypes in WT EV- and BsAb EV-treated lesions. **C** Distribution of T and NK cells from WT EVs (blue) and BsAb EVs (red) treatment groups. **D** Dot plot showing the average expression of genes associated with T cell function across groups. Dot size indicates the proportion of expressing cells; color intensity reflects average expression levels (white, low; red, high). **E** Inferred intercellular communication network within T/NK cells and between T/NK cells and other cell types. Number and edge thickness correspond to the number of ligand-receptor interactions. **F** UMAP plot visualizing mononuclear phagocytes (MPs) in fibrotic lesions. **G** Proportions of MP subtypes in WT EV- and BsAb EV-treated groups. **H** Distribution of MP cells from WT EVs (blue) and BsAb EVs (red) groups. **I** Volcano plot highlighting differentially expressed genes between BsAb EV- and WT EV-treated macrophages. Red points indicate significantly upregulated genes; blue points indicate significantly downregulated genes (adjusted *P* < 0.05, |log₂ fold change| > 0.25). **J** UMAP plot showing *Spp1* expression in MPs from both treatment groups. **K** Violin plot illustrating *Spp1* transcript levels in MPs from WT EV- and BsAb EV-treated groups. Embedded box plots denote median (center line), interquartile range (box limits, 25th to 75th percentile), and 1.5 × IQR (whiskers). WT EV, *n* = 8966 cells from 3 biological replicates; BsAb EV, *n* = 8808 cells from 3 biological replicates. For all quantitative data, data are presented as mean ± SEM. Comparisons between two groups were performed using two-tailed unpaired Student’s *t*-test, while comparisons among three or more groups were performed using one-way ANOVA followed by Tukey’s multiple comparisons test. Source data are provided as a Source Data file.

Given that BsAb EVs are designed to engage T cells, and that macrophages are central regulators of fibrotic remodeling, we next examined how BsAb EV treatment reshapes these two major immune compartments. T cells were classified into five major subsets: CD8⁺ naive, CD8⁺ effector, CD8⁺ memory, T helper, and T regulatory cells (Fig. 6A–C and Supplementary Fig. 12A). BsAb EV treatment led to an increased proportion of CD8⁺ naive, effector, and memory subsets, while T helper and Treg populations remained largely unchanged. Gene expression analysis revealed that CD8⁺ T cells in the BsAb EVs group exhibited elevated *Il7r* and Ccr7, accompanied by reduced expression of cytotoxicity-related genes, consistent with a transcriptional profile associated with memory differentiation and mild activation (Fig. 6D). Inferred cell–cell communication analysis also suggested enhanced interactions between T cells and both fibroblasts and mononuclear phagocytes (MPs) following treatment (Fig. 6E).

MPs were further resolved into seven transcriptionally distinct clusters, including macrophages and monocytes (Fig. 6F and Supplementary Fig. 12B). BsAb EV treatment was associated with a reduced macrophage fraction and an increased monocyte fraction (Fig. 6G, H). Macrophages from the BsAb EVs group showed increased expression of M2-associated genes, accompanied by reduced expression of *Spp1* and *Tgfb1*, genes previously linked to fibroblast activation (Fig. 6I–K and Supplementary Fig. 12D, E). Inferred cell–cell communication analysis further suggested weakened interactions between MPs and other populations (Supplementary Fig. 12C). Together, these findings indicate that BsAb EV treatment is associated with broad immune remodeling marked by shifts in T-cell and MP states, consistent with a less profibrotic microenvironment.

To further investigate the increased neutrophil abundance observed in the BsAb EVs group (Fig. 5B), functional interrogation of these neutrophils revealed that downregulated genes in the BsAb EVs group were significantly enriched in pathways related to inflammation, immune activation, and stress responses, suggesting a relatively quiescent, low‑activation phenotypic state (Supplementary Fig. 13A, B). However, in vitro experiments failed to detect any significant direct activation or chemotactic recruitment of neutrophils by BsAb EVs (Supplementary Fig. 13C–F). These findings suggest that the observed neutrophil dynamics may be secondary to BsAb EV-mediated fibroblast depletion rather than a direct effect on neutrophils.

### BsAb EVs alleviate fibrosis and promote peri-incisional tissue remodeling

**Fig. 7:**
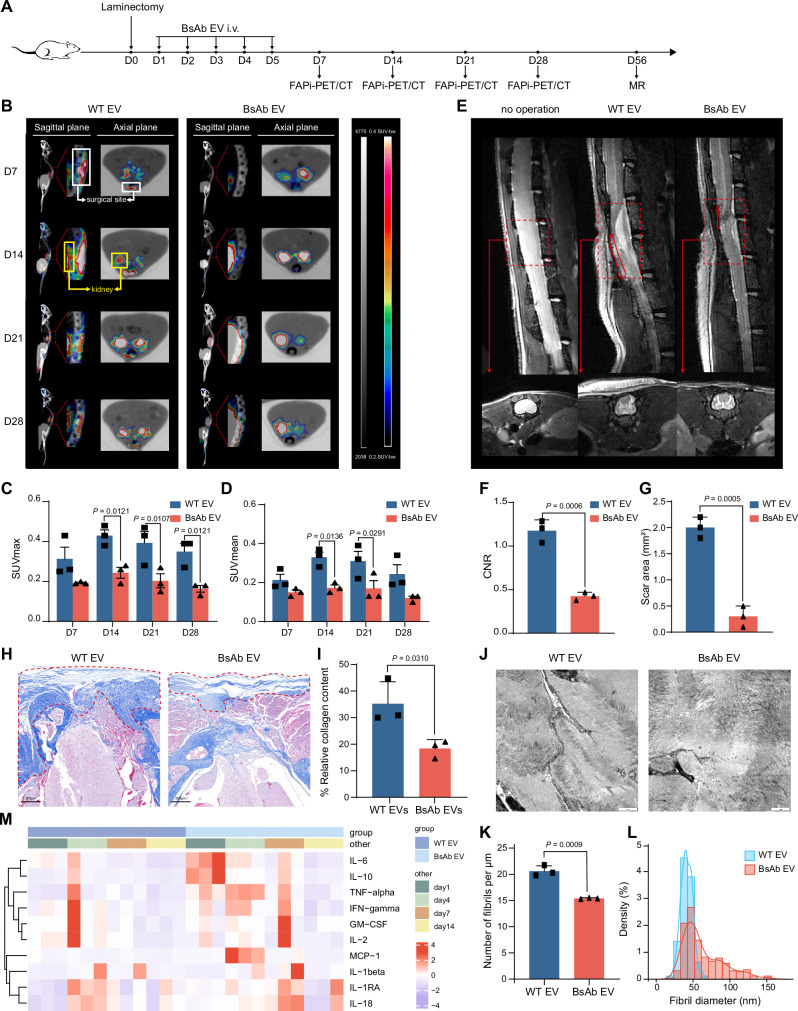
FAP × CD3 BsAb EVs reconstruct peri-incision tissue to improve fibrosis. **A** Schematic diagram of the in vivo experimental design. **B–D** Representative PET/CT images and quantification of maximum standardized uptake value (SUVmax) and mean standardized uptake value (SUVmean) in each treatment group at the indicated time points. Images include sagittal plane, magnified sagittal views, and axial plane. White boxes delineate the injury site; yellow boxes outline the kidneys (*n* = 3 mice per group). **E–G** Magnetic resonance imaging (MRI) evaluation of epidural scar formation and adhesion to the dura mater at 56 days post-laminectomy. Red boxes outline the scar tissue region (**E**). Bar graphs show the contrast-to-noise ratio (CNR) between epidural scar tissue and adjacent normal tissue, calculated as |S_L_–S_P_|/ σ_noise_, where SL and SP represent signal intensities of the lesion and paraspinal tissue, respectively, and σ_noise_ denotes the standard deviation of background noise (**F**, **G**) (*n* = 3 mice per group). **H**, **I** Representative Masson’s trichrome staining of laminectomy scar tissue at 56 days post-surgery (**H**) and corresponding quantification of collagen deposition scores (**I**). Red dashed curves delineate the fibrous capsule surrounding the dura mater (*n* = 3 mice per group, Scale bar, 200 μm). **J–L** Transmission electron microscopy analysis of fibroblast ultrastructure in the scar tissue at 56 days post-laminectomy (**J**) and quantitative analysis of collagen fiber density (**K**) and fiber diameter (**L**) (*n* = 3 mice per group, Scale bar, 2 μm). **M** Heatmap of differentially expressed serum cytokines in each treatment group at the indicated time points. Color scale represents Z-score of normalized cytokine levels (blue, low; red, high) (*n* = 3 mice per group). For all quantitative data, data are presented as mean ± SEM. Comparisons between two groups were performed using two-tailed unpaired Student’s *t*-test, while comparisons among three or more groups were performed using one-way ANOVA followed by Tukey’s multiple comparisons test. Source data are provided as a Source Data file.

We next examined whether BsAb EV treatment could attenuate EF and improve tissue remodeling following laminectomy. ^68^Ga-FAPI PET/CT imaging was performed weekly over 4 weeks to monitor FAP expression at the injury site in mice treated with BsAb EVs or WT EVs (Fig. 7A). As shown by PET/CT imaging (Fig. 7B–D), FAP signal peaked at day 14 post-laminectomy and declined thereafter. Compared with WT EV-treated controls, mice treated with BsAb EVs showed reduced FAP signals throughout the observation period, consistent with reduced FAP⁺ fibroblast abundance at the injury site. MRI at postoperative day 56 revealed dense epidural scar tissue in control mice, whereas BsAb EV-treated mice exhibited markedly reduced epidural scarring (Fig. 7E–G). In addition, BsAb EV-treated mice showed normal wound closure by postoperative day 56, with no evidence of impaired incisional healing compared with WT EV-treated controls (Supplementary Fig. 14A).

Representative H&E‑stained sections of the injury site at postoperative day 56 are shown in Supplementary Fig. 14A. Masson’s trichrome staining further demonstrated reduced collagen deposition across the tested BsAb EVs dose groups (Fig. 7H, I). TEM analysis of the BsAb EVs group showed sparser fibroblast filaments and the presence of larger-diameter filament clusters (Fig. 7J–L). Together, these findings indicate that BsAb EV treatment attenuates EF and improves postoperative tissue remodeling in vivo.

### BsAb EVs exhibit a favorable systemic safety profile

We next evaluated the systemic safety of BsAb EV treatment following laminectomy. Across the tested dose groups, mice showed no overt signs of systemic toxicity such as hyperthermia, body weight loss, or behavioral abnormalities, compared with controls (Supplementary Fig. 14B–D and Supplementary Table 2). To further assess potential hepatorenal toxicity, serum levels of alanine aminotransferase, aspartate aminotransferase, blood urea nitrogen, and creatinine were measured on postoperative days 7, 14, 27, and 56 (Supplementary Fig. 14E–H). Complete blood counts were evaluated at the same time points to monitor for potential hematological toxicity (Supplementary Fig. 14I–M). No significant differences were observed between the control and treatment groups in any of these parameters, indicating that BsAb EV treatment did not induce hepatorenal or hematological toxicity. No histological evidence of organ damage was observed in major solid organs from mice treated with different doses of BsAb EVs at postoperative day 56 (Supplementary Fig. 15).

Serum cytokine profiles were assessed in mice treated with WT EVs or BsAb EVs on postoperative days 1, 4, 7, and 14 (Fig. 7M and Supplementary Fig. 14N–W). In the BsAb EV-treated group, serum IL-6 and IL-10 showed mild transient increases on postoperative day 1, remaining below threefold above baseline (Supplementary Fig. 14N, O). Monocyte chemoattractant protein‑1 (MCP-1) was elevated on postoperative day 4 (Supplementary Fig. 14T), whereas IL-1RA, IL-1β, and IL-18 showed no significant differences between groups (Supplementary Fig. 14Q, U, W). Collectively, these results indicate that BsAb EV treatment is well tolerated in the tested perioperative mouse model without detectable acute hepatorenal, hematological, or major histological toxicity.

## Discussion

In this study, we identify FAP⁺ fibroblasts as a pathogenic stromal population enriched at postoperative spinal wound sites in both patients and murine models. These findings are consistent with the emerging role of FAP as a marker of activated fibroblasts across fibrotic and tissue-remodeling conditions, as also reflected by the expanding use of FAPI-based imaging in diseased tissues^26–31^. Our single-cell analyses further support this framework by showing that FAP⁺ fibroblasts are transcriptionally distinct from α-SMA⁺ myofibroblasts and are associated with ECM remodeling and TGF-β-related programs. Rather than establishing a definitive fibroblast lineage hierarchy, these data position FAP⁺ fibroblasts as a fibrogenic stromal state linked to pathological wound remodeling.

Consistent with this idea, FAP-directed immunotherapies have shown antifibrotic activity in preclinical models of cardiac fibrosis^22^. More recently, this concept was extended beyond conventional fibrotic disease settings to atherosclerosis^32^, where a FAP-directed BsAb strategy was used to target pathogenic vascular smooth muscle cells^33^. Our findings extend this concept to postoperative EF and support the broader applicability of FAP-directed immune redirection across non-malignant tissue-remodeling disorders.

Building on this conceptual framework, our BsAb-engineered EVs provide a cell-directed immunotherapeutic strategy that extends beyond conventional perioperative approaches based on physical separation or pharmacological modulation of fibrotic pathways. From a translational perspective, the EV platform may be particularly attractive for postoperative fibrosis, where lesions are localized and surgically accessible. BsAb EVs could potentially be administered directly to the wound site to enhance local efficacy while limiting systemic exposure. Moreover, the modular EV surface may enable future incorporation of therapeutic cargos, allowing combined immune redirection and local payload delivery within a single platform. By coupling lesion-directed localization with local administration, this strategy may further reduce off-target toxicity and improve therapeutic control in perioperative settings.

An important translational consideration is that FAP expression may not be restricted to the intended lesion. In one enrolled patient, prominent FAPI uptake was observed in the pectoralis minor outside the surgical field. Retrospective inquiry after imaging revealed a history of recent strenuous upper-extremity exercise, raising the possibility that non-target musculoskeletal remodeling may transiently increase FAP expression and thereby create a potential source of on-target, off-tissue toxicity for FAP-directed therapies. This observation requires validation in larger cohorts. If confirmed, recent intense musculoskeletal activity may need to be considered in pre-treatment screening, eligibility assessment, and avoided immediately post-therapy for future FAP-directed interventions.

Several limitations of this study should be acknowledged. First, the clinical FAPI PET/CT data provide only a single postoperative time point and therefore do not capture the temporal dynamics of FAP expression during scar development. In addition, direct histological correlation with the imaging signal was not available in this cohort. The clinical observations should be interpreted cautiously, as this part of the study was intended to provide supportive translational context rather than to define the precise determinants of surgical complexity. Second, our in vivo efficacy studies were performed in a mouse laminectomy model, and validation in rat or larger-animal models will be important to better assess surgical anatomy, dosing, biodistribution, and translational feasibility. Third, although local delivery may be attractive for surgically accessible postoperative lesions, BsAb EVs were administered systemically in this proof-of-concept study, and the optimal route of administration remains to be determined. Fourth, although our single-cell analyses provide a useful framework for identifying fibrogenic stromal states associated with postoperative remodeling, further mechanistic validation is needed. Finally, an additional limitation relates to the EV platform itself. The properties of EV-based platforms are strongly influenced by producer-cell origin^34,35^. The NIH/3T3 fibroblasts used here provide a robust engineering platform, but this cell line is not a clinically preferred EV source. Future studies should evaluate whether producer cells with greater translational relevance, such as mesenchymal stromal cells, can preserve therapeutic activity while offering advantages in biodistribution, immunogenicity, and clinical feasibility.

Overall, our study identifies FAP⁺ fibroblasts as a therapeutically actionable stromal population in postoperative EF and provides proof-of-concept for BsAb-engineered EVs as a strategy to redirect endogenous T cells, selectively deplete these cells, and attenuate pathological fibrotic remodeling.

## Methods

### Ethical statement

This prospective observational clinical study was approved by the Ethics Committee of Tongji Hospital, Huazhong University of Science and Technology (Approval No. TJ-IRB202407058). Written informed consent was obtained from all participants. Patients aged 18–70 years who underwent lumbar spinal decompression surgery (endoscopic surgery and open surgery) for lumbar disc herniation, spinal stenosis, or spondylolisthesis were enrolled. Exclusion criteria comprised uncontrolled comorbidities (e.g., infections, cardiovascular diseases), pregnancy, immunodeficiency, or severe allergies. Ten patients (5 open surgery, 5 endoscopic surgery) underwent postoperative ⁶⁸Ga-FAPI PET/CT imaging to assess FAP-associated postoperative tissue remodeling. Written informed consent was obtained for the publication of any potentially identifiable images.

The retrospective analysis of 207 patients who underwent TLIF at our center was approved by the Ethics Committee of Tongji Hospital, Huazhong University of Science and Technology (Approval No. TJ-IRB20230770). The requirement for written informed consent was waived by the ethics committee because this was a retrospective study of de-identified patient records, and the research involved no more than minimal risk to participants, in accordance with Article 39 of the Measures for the Ethical Review of Biomedical Research Involving Humans (National Health and Family Planning Commission Order No. 11, 2016).

### Sex as a biological variable

Our study included both male and female patients, as well as both male and female mice. Sex-specific analyses were not performed, and it is unknown whether the findings are differentially relevant across sexes.

### Cell culture and mice

The human chronic myeloid leukemia blast crisis cell line K562, and the mouse fibroblast cell line NIH/3T3 were sourced from the American Type Culture Collection (Manassas, VA, USA). NIH/3T3 cells were cultured in Dulbecco’s Modified Eagle Medium (DMEM, high glucose; Hyclone, Logan, UT, USA) supplemented with 10% fetal bovine serum (FBS; Hyclone) at 37 °C in a 5% CO_2_ atmosphere. K562 and K562-FAP cells were maintained in RPMI 1640 medium with 10% FBS, under the same conditions.

All animal experiments and procedures were conducted in accordance with the guidelines approved by the Institutional Animal Care and Use Committee of Tongji Medical College, Huazhong University of Science and Technology (Ethics approval number: TJH‑202407006). BALB/c mice (5–7 weeks old) were obtained from Beijing Weitong Lihua Experimental Animal Technology Co., Ltd. (Beijing, China) and housed under specific pathogen-free conditions. The mice were kept in a controlled environment with a 12-h light/12-h dark cycle, 40–50% relative humidity, and a temperature of 22 °C. They were regularly monitored for health and exhibited no apparent behavioral abnormalities.

FAP-deficient mice (*Fap*^LacZ/LacZ^) on a BALB/c background were used as FAP-deficient mice, as previously described in refs. ^36,37^. Age- and sex-matched WT BALB/c mice were used as controls. Both male and female mice were included in the study.

### Laminectomy mouse model

To create a laminectomy model, mice were anesthetized with an intraperitoneal injection of 30 μL/g of 1.25% tribromoethanol. The animals were positioned in the prone stance with their limbs secured on the operating table. A longitudinal incision of approximately 2 cm was made along the spine on the dorsal aspect of the mouse. The incision edges were retracted using a retractor, and the surrounding muscles were carefully separated along the spinal spinous process to fully expose the T12-L2 spinous processes and the adjacent laminae. The T12-L2 laminae were then grasped with forceps, and the spinous processes were removed to reveal the dura mater. Following the procedure, the fascia, muscles, and skin were sutured to close the incision.

### FAP inhibitor (FAPi) treatment

For pharmacological inhibition of FAP, mice received daily intraperitoneal injections of the small-molecule FAP inhibitor Ac-Gly-BoroPro (FAPi; MedChemExpress, catalog HY-101801) at a dose of 0.5 mg/kg body weight, or an equivalent volume of sterile PBS as vehicle control. Treatments were initiated on the day of laminectomy (day 0) and continued for five consecutive days (days 0–4 post-surgery). The dosing regimen was selected based on prior pharmacokinetic studies of Ac-Gly-BoroPro in murine fibrosis models.

### In vivo T cell depletion

To deplete T cells in vivo, mice received intraperitoneal injections of anti-CD3ε monoclonal antibody (clone 145-2C11, Bio × Cell, West Lebanon, NH, USA) at a dose of 10 µg per mouse in 200 µL sterile PBS on postoperative days −4, −2, and 0 relative to laminectomy. Control mice received an equivalent volume of hamster IgG isotype control antibody (Bio × Cell). T cell depletion efficiency was confirmed by flow cytometric analysis of splenic CD3⁺ T cells at the time of tissue harvest.

### Flow cytometry

All flow cytometry analyses of fibroblasts described in this manuscript were conducted on dissociated primary fibroblasts following lineage-negative gating to exclude hematopoietic, endothelial, and epithelial cell lineages. The lineage markers used included CD31, CD45, Tie2, Ter119, and EpCAM (CD326). During the harvesting protocol, cells were stained with a single APC-conjugated antibody, along with PE-conjugated antibodies against CD31 (clone W18222B, BioLegend, catalog 160203, Rat IgG2b, κ), CD45 (clone TEK4, BioLegend, catalog 103105, Rat IgG1), Tie2 (clone 30-F11, BioLegend, catalog 124007, Rat IgG2b), Ter119 (clone TER-119, BioLegend, catalog 116207, Rat IgG2b), and EpCAM (clone G8.8, BioLegend, catalog 118205, Rat IgG2a). Activated T cells were marked with CD3 (clone 17A2, BioLegend, catalog 100220, Rat IgG2b), CD69 (clone H1.2F3, BioLegend, catalog 104511, Armenian Hamster IgG), and CD25 (clone 3C7, BioLegend, catalog 101918, Rat IgG2b). Neutrophil purity was confirmed by flow cytometry using Ly6G (clone 1A8, BioLegend, catalog 127607 1, Rat IgG2a, κ) and CD11b (clone M1/70, BioLegend, catalog 101205, Rat IgG2b, κ) double positivity. Following staining, cells were resuspended in FACS buffer containing DAPI (Biolegend, catalog 422801) prior to flow cytometry analysis.

### Luciferase assays

Primary mouse T cells were purified from spleens using Stemcell negative selection T cell isolation kits (catalog 19853). Dynabeads Mouse T-Activator CD3/CD28 (Gibco, catalog 11452D) is intended for activation of mouse T cells. K562-FAP cells-expressing Luc served as target cells for luciferase cytotoxicity assay protocols. In brief, 1 × 10^4^ target cells were resuspended in 100 µL of assay medium (RPMI 10% FBS), co-cultured with 5 × 10^4^ or 1 × 10^5 ^T cells in 50 µL of assay medium, and different concentrations of FAP × BsAb EVs (volume 50 µL) were added for 24 h in the incubator and in technical triplicates. D-luciferin (Gold-Bio, St. Louis, MO, USA) was dissolved in pure water to a final concentration of 15 mg/mL and stored at −20 °C. To generate the working assay solution, a stock aliquot was mixed 1:4 in RPMI 10% FBS and 20 µL were added to each assay well (final volume 220 µL) immediately before the luminescence measurement in TECAN Spark reader (TECAN, Männedorf, Switzerland). Effector-specific lysis was calculated according to spontaneous lysis (target cells in assay medium with luciferin) and maximum killing controls (target cells in assay medium with Triton ×-100 and luciferin).

### Isolation of fibroblasts

Euthanize the mouse with a 200 mg/kg intraperitoneal injection of Fatal-Plus in 500 μL saline. Dissect the surgical tissue and place it into a 100 mm tissue culture Petri dish. Mince the tissue into small pieces using a razor blade or surgical scissors. Transfer the minced tissue to a 1.5 mL microcentrifuge tube and add 0.5 mL of collagenase (final concentration: 1000 U/mL). Incubate at 37 °C for 60 min. After incubation, centrifuge the suspension at 1000 × *g* for 5 min at room temperature (24 °C). Carefully decant and discard the supernatant. Wash the pellet by adding 1 mL of HBSS, then centrifuge again at 1000 × *g* for 5 min at room temperature. Carefully decant and discard the supernatant. Add 0.5 mL of 0.25% Trypsin-EDTA to the pellet, mix thoroughly, and incubate at 37 °C for 20 min. After incubation, centrifuge the sample at 1000 × *g* for 5 min at room temperature. Carefully decant and discard the supernatant. Resuspend the pellet in 0.5 mL of DMEM containing 15% FBS. Gently pipette up and down to disaggregate cell clusters. Plate the cell suspension into a well of a 6-well tissue culture plate, discarding any large tissue fragments. Add 2 mL of DMEM containing 15% FBS to achieve a final culture volume of approximately 2.5 mL. Incubate the cells at 37 °C in a 5% CO_2_ atmosphere.

### Collagen gel contraction assays

The contraction assay was conducted to assess the contractility of myofibroblasts. Collagen gels were prepared by mixing fibroblast cell suspensions in serum-free medium with type I collagen solution (Corning, catalog 354249). The final cell density was 2.0 × 10^5^ cells/mL, and the collagen concentration was 1 mg/mL. A 0.5 mL aliquot of the mixture was cast into each well of a 24-well plate and allowed to polymerize for 30 min at 37 °C. After polymerization, an additional 0.5 mL of serum-free DMEM was added to each well. The gels were observed at fixed distances above the gels at 12 and 24 h. Changes in gel surface area were quantified using ImageJ software. The percentage of contraction was calculated using the formula:

1 $${{{\rm{Percentage}}}}\; {{{\rm{of}}}}\; {{{\rm{Contraction}}}}=100\%\times \frac{({{{\rm{well}}}}\; {{{\rm{surface}}}}\; {{{\rm{area}}}}-{{{\rm{gel}}}}\; {{{\rm{surface}}}}\; {{{\rm{area}}}})}{{{{\rm{well}}}}\; {{{\rm{surface}}}}\; {{{\rm{area}}}}}$$

### FAP × CD3 BsAb EVs lentiviral vector design and transfection

FAP × CD3 BsAb sequences were designed with a CD8 leader peptide, scFvs targeting murine CD3 (clone 145-2C11) and FAP (clone 73.3), followed by a CD8 hinge and transmembrane domain. The entire construct was fused to the GFP sequence to facilitate quantitative analysis. After synthesizing, the constructs were cloned into a lentiviral plasmid backbone regulated by a human EF-1α promoter. We established the anti-FAP × CD3-NIH/3T3 cell line by transfecting the FAP × CD3 BsAb lentivirus into the NIH/3T3 cell line and then sorting by flow cytometry to harvest producer cells.

### Generation and isolation of EVs

Before the generation of EV, exosomes were removed from the FBS to culture producer cells. NIH/3T3 and anti-FAP × CD3-NIH/3T3 cells were exposed to ultraviolet irradiation (UVB, 300 J/m^2^) for 1 h. The culture medium was first centrifuged at 500 × *g* for 10 min to get rid of cells and then passed through 0.45-μm sterile cellulose acetate or polysulfonate syringe filter to remove cellular debris. At last, the supernatant was further centrifuged for 60 min at 14,000 × *g* (Avanti J-26S XPI High-Performance Centrifuge; Beckman Coulter, Brea, CA, USA) to pelletize the EV. The purified EVs were washed and resuspended in sterile phosphate-buffered saline (PBS) and used fresh or stored frozen at −80 °C until further use. All processes were performed at 4 °C. To confirm the size of the EVs that were isolated, samples were analyzed by Nanoparticle Tracking Analysis (NTA). The protein concentration of EV was assayed by a BCA Protein Assay Kit (Beyotime, China).

### Quantification of BsAb copy number per EV particle

GFP fluorescence was used as a proxy to estimate the loading of GFP-tagged BsAb constructs per EV particle. Purified BsAb EVs were first subjected to nanoparticle tracking analysis (ZetaView PMX120-Z, Particle Metrix) to determine the particle concentration (particles/mL). In parallel, the molar concentration of GFP-tagged BsAb protein in the same EVs preparation was quantified by fluorescence spectrophotometry against a GFP recombinant protein standard curve of known concentration. The average construct loading per EV particle was then calculated as: Copy number per EV = (BsAb molar concentration × Nₐ)/EV particle concentration (2), where *N*ₐ is Avogadro’s constant (6.022 × 10²³ mol⁻¹). Measurements were performed on three independent EV batches to assess batch-to-batch reproducibility.

### DiR/DID labeling of EVs

For in vivo tracking experiments, purified WT EVs and BsAb EVs were labeled with the lipophilic fluorescent dye DiR/DID. Briefly, DiR/DID powder (5 mg) was dissolved in 1 mL DMSO to prepare a 5 mmol/L stock solution. A working solution (1 mmol/L) was freshly prepared by diluting 10 μL of DiR/DID stock solution with 40 μL PBS. Purified EVs (990 μL) were incubated with 10 μL of DiR/DID working solution (final DiR/DID concentration, 10 μmol/L) for 30 min at room temperature in the dark. Following incubation, the labeled EV suspension was transferred to a high-speed centrifuge tube, diluted with 40 mL PBS, and centrifuged at 18,000 × *g* for 1 h at 4 °C to remove unincorporated dye. The resulting DiR/DID-labeled EVs pellet was resuspended in 400 μL sterile PBS and used immediately for in vivo imaging experiments.

### Micro-PET/CT

^68^Ga-FAPI was selected as the radiotracer^38^. Micro-PET/CT imaging of mice was performed using a micro-PET/CT scanner (Siemens, Erlangen, Germany). Mice were positioned lying down and given injections of 7.40–9.25 MBq of ^68^Ga-FAPI via the tail vein for either static or 60 min post-injection PET/CT scans.

### Magnetic resonance imaging

Magnetic resonance imaging (MRI) was generated 2 months after spine surgery. These mice were anesthetized with isoflurane and scanned at the T11-L4 spine. MRI measurements were carried out on a 9.4 T MR scanner with a 30 cm diameter bore (μMR 9.4 T, United Imaging Life Science Instrument, Wuhan, China). A fast spin echo T2-weighted imaging (FSE-2D T2WI) sequence with respiratory triggering was used, with the following parameters: Transverse: TR: 1864 ~ 3244 ms, TE: 47.18 ms, Bandwidth: 220 Hz, ETL: 13, FOV: 14 × 20 mm^2^, Slice thickness: 0.5 mm, Gap: 0 mm, Slice: 20, Resolution: 0.08 × 0.08 × 0.5 mm^3^, NEX:6; Sagittal: TR: 1904 ~ 3076 ms, TE: 47.18 ms, Bandwidth: 220 Hz, ETL: 13, FOV: 14 × 20 mm^2^, Slice thickness: 0.5 mm, Gap: 0 mm, Slice: 20, Resolution: 0.08 × 0.08 × 0.5 mm^3^, NEX: 4. The epidural scar area was measured by U_VIEWER software.

### Transmission electron microscopy

20 μL of the resuspended EVs shed from NIH/3T3 and anti-FAP×CD3-NIH/3T3 cells were added dropwise to 200-mesh grids and incubated at room temperature for 10 min, then the grids were negatively stained with 2% phosphotungstic acid for 3 min, and the remaining liquid was removed by filter paper. Then samples were observed with a JEM1400 transmission electron microscope.

Samples were fixed in 2% glutaraldehyde in PBS at 4 °C for 3 h. Fixed samples were embedded in low-viscosity resin as following: samples were osmicated using 1% OsO4; stained with 3% uranyl-acetate; dehydrated in 50–70–80–90–95–100% ethanol and embedded in low-viscosity resin. Plastic-embedded samples were sectioned using UCT ultramicrotome (Leica, Austria) and diamond knife (Diatome, Austria). Sections 60–80 nm thick were mounted on home-made EM grid(s) with plastic-carbon support film, stained with saturated uranyl-acetate and Sato’s lead-citrate. Sections were imaged using JEM-1400 transmission electron microscope (JEOL Japan Electronics) at 80 kV equipped with BioScan600W digital camera (Gatan, USA). Images were prepared for publication using a Photoshop (Adobe, USA). Approximately 300 fibrils were measured by ImageJ for analyzing fibril diameter in each group.

### Nanoparticle tracking analysis (NTA)

EVs were analyzed and quantified via NTA with Zetaview PMX120-Z (Particle Metrix, Meerbusch, Germany) and corresponding software ZetaView (version 8.05.14 SP7). Isolated exosome samples were appropriately diluted using 1× PBS buffer (Beyotime, China) to measure the particle size and concentration. NTA measurement was recorded and analyzed at 11 positions. The ZetaView system was calibrated using 110 nm polystyrene particles. Temperature was maintained around 23 and 30 °C.

### Untargeted lipidomics analysis

Sample aliquots (100 µL) were extracted with 300 µL of ice-cold methanol:acetonitrile (1:1, v/v) containing 0.5 µg/mL 2-chloro-L-phenylalanine internal standard. After vortexing and centrifugation (15,000 × *g*, 15 min, 4 °C), supernatants were analyzed using a Thermo Vanquish UHPLC coupled to an Orbitrap Exploris 120 mass spectrometer. Separation was performed on a Thermo Hypersil GOLD C18 column (2.1 × 100 mm, 1.9 µm) with mobile phases consisting of (A) acetonitrile:water (60:40, v/v) and (B) isopropanol:acetonitrile (90:10, v/v), both containing 0.1% formic acid and 10 mM ammonium acetate. A 15-min gradient elution was applied at 0.3 mL/min and 40 °C. Full-scan MS data (*m/z* 85–1250) were acquired at 60,000 resolutions in both positive and negative ESI modes, with data-dependent MS/MS using stepped NCE (10, 30, 50). Data were processed using Compound Discoverer (v3.1, Thermo Fisher Scientific) with lipid identification against the LipidSearch database (v4.2) and quantification by relative abundance normalized to total lipid signal.

### Bulk RNA sequencing

Three mice were used per group. RNA was extracted from the matched samples using RNeasy Kits (Qiagen, catalog 75144) and treated with DNase I (Thermo Fisher Scientific, catalog AM2222) according to standard protocols. RNA-sequencing was done in Genesky using TruSeq Stranded mRNA Library Preparation (Genesky). Briefly, intact RNA was fragmented, end repaired, adapter ligation and PCR amplified following the Illumina protocol. Libraries were sequenced by Illumina HiSeq 2000. After quality control, sequence data were processed with STAR to generate read alignments with GRCm38/mm10. Raw read counts for annotated genes were obtained with featureCounts with default settings, normalized and analyzed using DESeq2. Real-time PCR was used to validate the RNA-seq data.

### Single-cell RNA sequencing

#### Tissue dissociation and preparation

Three mice were used per group. Fresh tissues were preserved in sCelLive^TM^ Tissue Preservation Solution (Singleron) on ice within 30 min post-surgery. Specimens were washed with HBSS, minced, and digested with 3 mL sCelLiveTM Dissociation Solution (Singleron) (37 °C, 15 min) using Singleron PythoN™ Tissue Dissociation System. After filtration (40 μm strainer), red blood cells were lysed with GEXSCOPE® RCLB (1:2 ratio, 5–8 min, RT). The mixture was then centrifuged at 300 × *g* 4 °C for 5 min to remove supernatant, followed by PBS resuspension. Cell viability was assessed via Trypan Blue staining.

### RT & amplification & library construction

Single-cell suspensions (2 × 10^5^ cells/mL in PBS) were loaded onto a microwell chip using the Singleron Matrix® Single Cell Processing System. Barcoding Beads were collected from the chip, and reverse transcription was performed to generate cDNA from captured mRNA, followed by PCR amplification. The amplified cDNA was fragmented, ligated with sequencing adapters, and libraries were constructed using GEXSCOPE® Single Cell RNA Library Kits (Singleron)^22^. Libraries were diluted to 4 nM, pooled, and sequenced on an Illumina Novaseq 6000 platform with 150 bp paired end reads.

### Single-cell RNA-seq data processing

Raw sequencing data were processed using CeleScope v3.0.1 (Singleron Biotechnologies) with default settings. Cell barcodes and UMIs were extracted and corrected from Read 1, while Read 2 was trimmed to remove adapter sequences and poly-A tails and aligned to the mouse reference genome (GRCm38/mm10) using STAR (v2.6.1b). Uniquely mapped reads were assigned to exons using FeatureCounts(v2.0.1), and UMIs with identical cell barcodes and gene annotations were collapsed to generate a gene expression matrix.

### Quality control, normalization, and clustering

Quality control and downstream analyses were performed using Scanpy v 1.9.8 (Python 3.7)^39^. Cells were excluded if they met any of the following criteria: fewer than 200 detected genes, top 2% of gene or UMI counts, or >5% mitochondrial gene content. Genes detected in fewer than 5 cells were also removed. A total of 93439 high-quality cells were retained, with a median of 1711 genes and 5027 UMIs per cell.

Gene counts were normalized to total transcript counts per cell and log-transformed. The top 2000 highly variable genes were identified using the “seurat” method, followed by principal component analysis. The top 20 principal components were used for dimensionality reduction and clustering via the Louvain algorithm (resolution = 1.2). Clusters were visualized using Uniform Manifold Approximation and Projection (UMAP).

### Batch correction and contaminant/doublet filtering

To correct for inter-sample batch effects, Harmony v0.0.6 was applied to the top 20 principal components^40^. Putative doublets were identified based on co-expression of lineage-specific markers and manually excluded. Ambient RNA contamination was mitigated using the DecontX algorithm from the celda package (v.1.6.1), which models ambient transcripts as a mixture of inferred population transcriptomes via Bayesian variational inference. Raw UMI count matrices and clustering results from Seurat v5.1.0 were used as input, and a default random seed ensured reproducibility.

### Enrichment analysis

Cell-type enrichment across conditions was assessed by the ratio of observed to expected cell numbers (Ro/e), calculated as: Ro/e = Observed/Expected (3). Expected cell number were derived using chi-squared tests. A Ro/e > 1 indicates enrichment of a given cell type in the specified group beyond random expectation^41^.

### Differentially gene expression analysis

DEGs were identified using the rank_genes_groups function in Scanpy, based on the Wilcoxon rank-sum test. Genes expressed in > 10% of cells in either group and with an average log (Fold Change) > 1 were considered DEGs. Multiple testing correction was performed using the Benjamini–Hochberg method, with an adjusted *p*-value threshold of 0.05.

### Pathway enrichment analysis

Functional enrichment of DEGs was assessed using the Kyoto Encyclopedia of Genes and Genomes (KEGG) database via the “clusterProfiler” R package (v 3.16.1)^42^. Pathways with adjusted *p*-value < 0.05 were considered significantly enriched.

### Cell type annotation

Cell identities were assigned based on the expression of canonical markers from the SynEcoSys^TM^ reference database (Singleron Biotechnology), which integrates annotations from CellMakerDB, PanglaoDB, and recently published literatures^43^.

### Subclustering of major cell populations

To refine cellular resolution, major populations, including fibroblasts, mononuclear phagocytes (MPs), and T/NK cells were subsetted and reanalyzed following the same pipeline. Louvain clustering resolutions were set to 1.0 (fibroblasts), 1.2 (MPs), and 1.2 (T/NK cells).

### Cell–Cell communication analysis (CellChat)

Intercellular communication was inferred using CellChat (v1.6.1). A CellChat object was constructed with cell metadata and ligand-receptor interactions from the built-in database. Probabilistic inference of signaling networks was performed using default parameters^44^.

### Pseudotime trajectory inference

Cell fate trajectories of monocyte subsets were reconstructed using Monocle2 (v2.22.0). The top 2000 highly variable genes were selected using the FindVariableFeatures function in Seurat (v5.1.0), and dimensionality reduction was performed using DDRTree. Trajectories were visualized using plot_cell_trajectory^45^.

### Gene set scoring

Gene signature activity was quantified using UCell (v2.6.2), a rank-based method leveraging the Mann–Whitney *U* statistic. UCell scoring is robust to batch variation and optimized for large-scale single-cell datasets^46^.

### Neutrophil isolation and functional assays

Murine bone marrow neutrophils were isolated from the femurs and tibias of 8–12-week-old BALB/c mice using a density gradient centrifugation method (Histopaque 1077/1119, Sigma-Aldrich). Purity was confirmed to be >90% by flow cytometry using Ly6G and CD11b staining. Isolated neutrophils (1 × 10⁵ cells/well) were incubated with DiD-labeled WT EVs or BsAb EVs (40 nmol) in serum-free RPMI 1640 medium for 4 h at 37 °C. After washing, EV uptake was analyzed by flow cytometry (CytoFLEX, Beckman Coulter). Neutrophils were incubated with WT EVs or BsAb EVs for 2 h at 37 °C. ROSUP (100 μM) was used as a positive control. Reactive oxygen species (ROS) production was measured using a ROS assay kit (Beyotime) according to the manufacturer’s instructions, with fluorescence intensity quantified on a microplate reader (TECAN Spark).

### Western blot

After washing the cells with PBS thrice, the cells were lysed using RIP lysis buffer. The cell lysate was centrifuged at 14,000 × *g* for 15 min at 4 °C. After centrifugation, the supernatant was collected. Protein concentrations were then determined using the BCA kit. The protein samples were heated at 98 °C for 5 min and then immediately placed on ice for 5 min. 30 μg/well (protein sample) was subjected to SDS-PAGE analysis, and then the protein samples were transferred to PVDF membrane. After washing, the cell samples were blocked with 5% BSA at room temperature for 2 h in a shaker. After washing, the PVDF membranes were incubated with the indicated primary antibodies (FAP, Abcam, catalog ab207178, Rat IgG; GFP, Abcam, catalog ab290, Rat IgG; Annexin A1, Huabio, catalog ER1803-70, Rat IgG; Annexin V, Huabio, catalog ET1702-62, Rat IgG; β-tubulin, Proteintech, catalog 10094-1-AP, Mouse IgG; Calnexin, Proteintech, catalog 10427-2-AP, Rabbit IgG; TSG101, Proteintech, catalog 28283-1-AP, Rabbit IgG; CD63, Proteintech, catalog 25682-1-AP, Rabbit IgG) for 12 h at 4 °C, followed by incubation with secondary antibodies at 37 °C for 1 h. After the final three washes, the ECL system was used to visualize protein blots.

### Real-time PCR

RNA was isolated from snap-frozen tissues and extracted with Trizol reagent (Takara) according to the manufacturer’s instruction. cDNA was synthesized using High-Capacity cDNA Reverse Transcription Kit (Takara). Real-time quantitative PCR was carried out using SYBR Green Real-Time PCR Master Mix on benchtop Bio-Rad CFX96. Primer sequences are listed in Supplementary Table 3.

### Cytokine measurements

In brief, whole blood from mice was collected into procoagulant tubes and subjected to centrifugation at 4 °C and 1500 × *g* for 20 min. Subsequently, the mouse serum was isolated and stored at −80 °C. Luminex Performance Mouse XL Cytokine Panel (10-Plex) FCSTM20-10 (R&D Systems) contains MCP-1, GM-CSF, IFN-γ, IL-1β, IL-1RA, IL-2, IL-6, IL-10, IL-18, TNF-α. Bio-Plex 200 systems (Bio-Rad) was used to measure cytokine and chemokine secretion as suggested by the manufacturer.

### Immunohistochemistry

For immunohistochemistry (IHC), tissues were formalin fixed and paraffin-embedded. Five micrometer sections were rehydrated and antigen retrieval was performed in 10 mM citrate buffer (pH 6.0) or TE buffer (pH 9.0) for a period of 15 min to 1 h. After 1 h blocking with appropriate blocking buffer at room temperature, the sections were incubated overnight at 4 °C with primary antibody (FAP, Abcam, catalog ab207178, Rat IgG; α-SMA, Abcam, catalog ab5694, Rat IgG) and then with secondary antibody for 1 h at room temperature. For IHC, ABC reagent (Vector Laboratories, West Grove, PA) was applied and the sections were developed using diaminobenzidine reagent (DAB) according to the manufacturer’s instructions. For IHC staining of FAP and α-SMA, three to five non-overlapping, representative fields of view were randomly captured from each tissue section at 100× magnification using a light microscope equipped with a digital camera. The resulting images were analyzed to determine the positive staining area percentage. Briefly, color images were converted to 8-bit grayscale. A consistent color threshold was applied to selectively highlight the brown DAB positive signals, while masking the hematoxylin counterstain. The area of the thresholded positive pixels was then measured and expressed as a percentage of the total tissue area within the field of view. The mean value from all fields per sample was calculated and used for subsequent statistical analysis.

For Masson Trichrome staining, sections were stained using Masson Trichrome Stain kit (Thermo Scientific, 87019). The relative collagen content was quantified by applying a specific color threshold to identify the blue-stained collagen fibers. The collagen-positive area was measured and divided by the total tissue area of the field of view, yielding the percentage of collagen area for each image.

All threshold settings were predetermined and maintained constant for the analysis of all images within the same experimental group to ensure comparability. The investigators performing the quantitative analysis were blinded to the group assignments throughout the process.

### Immunofluorescence staining

For dual-color immunofluorescence analysis, formalin-fixed paraffin-embedded tissue sections were deparaffinized, rehydrated, and subjected to antigen retrieval in 10 mM citrate buffer (pH 6.0) for 15 min. After blocking with 5% bovine serum albumin (BSA) in PBS for 1 h at room temperature, sections were incubated overnight at 4 °C with primary antibodies (FAP, Abcam, catalog ab207178, Rat IgG; CD3, Proteintech, catalog 17617-1-AP, Rat IgG) diluted in blocking buffer. Following three washes with PBS, sections were incubated with fluorophore-conjugated secondary antibodies for 1 h at room temperature in the dark. Nuclei were counterstained with DAPI. Images were acquired using a confocal laser scanning microscope and analyzed with ImageJ software. Colocalization profiles were generated by line-scan analysis across regions of interest. Detailed antibody information for each specific staining panel is provided in the corresponding figure legends.

### Statistical analysis

The experimental data were analyzed utilizing GraphPad Prism version 8.0. Statistical analysis for comparisons between two groups was conducted using Student’s *t*-test. For comparisons involving multiple groups (three or more), a one-way analysis of variance (ANOVA) was performed, followed by pairwise multiple comparisons. Unless otherwise indicated, data are presented as mean ± SEM. For analyses intended to summarize batch-to-batch variability, values are presented as mean ± SD. All graphical representations were produced using GraphPad Prism version 8.0. Each experiment was conducted a minimum of three times.

### Reporting summary

Further information on research design is available in the Nature Portfolio Reporting Summary linked to this article.

## Supplementary information

Supplementary Information

Reporting Summary

Transparent Peer Review file

## Source data

Source data

## Data Availability

The bulk RNA-seq and single-cell RNA-seq data generated in this study have been deposited in the GEO database under accession code GSE310989 [GEO Accession viewer] and GSE310366 [GEO Accession viewer]. The EV lipidomics data generated in this study have been deposited in the Metabolomics Workbench Project ID PR003148 and Study ID ST004914 [10.21228/M8S27C]^47^. All other data generated in this study are provided in the Supplementary Information and Source Data file. Materials generated in this study, including plasmids, engineered producer cells, and FAP × CD3 BsAb EVs, are available upon request and may require completion of a Materials Transfer Agreement. Requests should be directed to Z.Z. (zhangzhengtj1992@gmail.com). Source data are provided with this paper.
