# Supplementary material for: Bispecific antibody engineered extracellular vesicles redirect T cells to prevent postoperative epidural fibrosis
Source: Nat Commun. 2026 Jun 27;17:8054. doi: 10.1038/s41467-026-74883-3 (PMC13454492; doi:10.1038/s41467-026-74883-3)
Supplement: Supplementary file 1 [file 41467_2026_74883_MOESM1_ESM.pdf]

Supplementary Information

Manuscript Title: Bispecific Antibody Engineered Extracellular Vesicles Redirect T Cells to Prevent Postoperative Epidural Fibrosis

Authors: Shanwei Ye[1,2,3,4]†, Qian Xu[2,5]†, Yong Xu[1], Hui Lu[6], Zhenfeng Liu[7], Jianfeng Guo[1], Yichuan Li[8], Wei Wang[9], Jun Ran[10], Xiaohua Zhu[11], Dongling Zhu[11], Wei Wu[1], Zechuan Yang[1], Shiqi Gu[12], Feng Li[1], Lugui Qiu[3,4], Ellen Puré[13], Vijay G. Bhoj[5], Liang Huang[3,4]*, Wei Xiong[1]*, Zheng Zhang[1,5]*

Affiliations:

[1]Department of Orthopedics, Tongji Hospital, Tongji Medical College, Huazhong University of Science and Technology, Wuhan, Hubei, 430030, China.

[2]Department of Hematology, Tongji Hospital, Tongji Medical College, Huazhong University of Science and Technology, Wuhan, Hubei, 430030, China.

[3]State Key Laboratory of Experimental Hematology, National Clinical Research Center for Blood Diseases, Haihe Laboratory of Cell Ecosystem, Institute of Hematology & Blood Diseases Hospital, Chinese Academy of Medical Science & Peking Union Medical College, Tianjin, 300020, China.

[4]Tianjin Institutes of Health Science, Tianjin, 301600, China.

[5]Department of Pathology & Laboratory Medicine, University of Pennsylvania, Perelman School of Medicine; Philadelphia, PA, 19104, USA.

[6]Department of Orthopedics, The First Affiliated Hospital, Zhejiang University School of Medicine; Hangzhou, Zhejiang, 310003, China.

[7]Department of Nuclear Medicine, The First Affiliated Hospital, Zhejiang University School of Medicine; Hangzhou, Zhejiang, 310003, China.

[8]Department of Dermatology, Tongji Hospital, Tongji Medical College, Huazhong University of Science and Technology, Wuhan, Hubei, 430030, China.

[9]Department of Surgery, University of Pennsylvania, Perelman School of Medicine, Philadelphia, PA 19104, USA.

[10]Department of Radiology, Tongji Hospital, Tongji Medical College, Huazhong University of Science and Technology, Wuhan, Hubei, 430030, China.

[11]Department of Nuclear Medicine, Tongji Hospital, Tongji Medical College, Huazhong University of Science and Technology, Wuhan, Hubei, 430030, China.

[12]Institute of Organ Transplantation, Tongji Hospital, Tongji Medical College, Huazhong

University of Science and Technology, Wuhan, Hubei, 430030, China.

[13]Department of Biomedical Sciences, University of Pennsylvania, Philadelphia, PA 19104, USA.

†These authors contributed equally to this work

*Corresponding authors:

Zheng Zhang

zhangzhengtj1992@gmail.com

Wei Xiong

xcxgreatwellus@hotmail.com

Liang Huang

huangliang@ihcams.ac.cn

Supplementary Figures

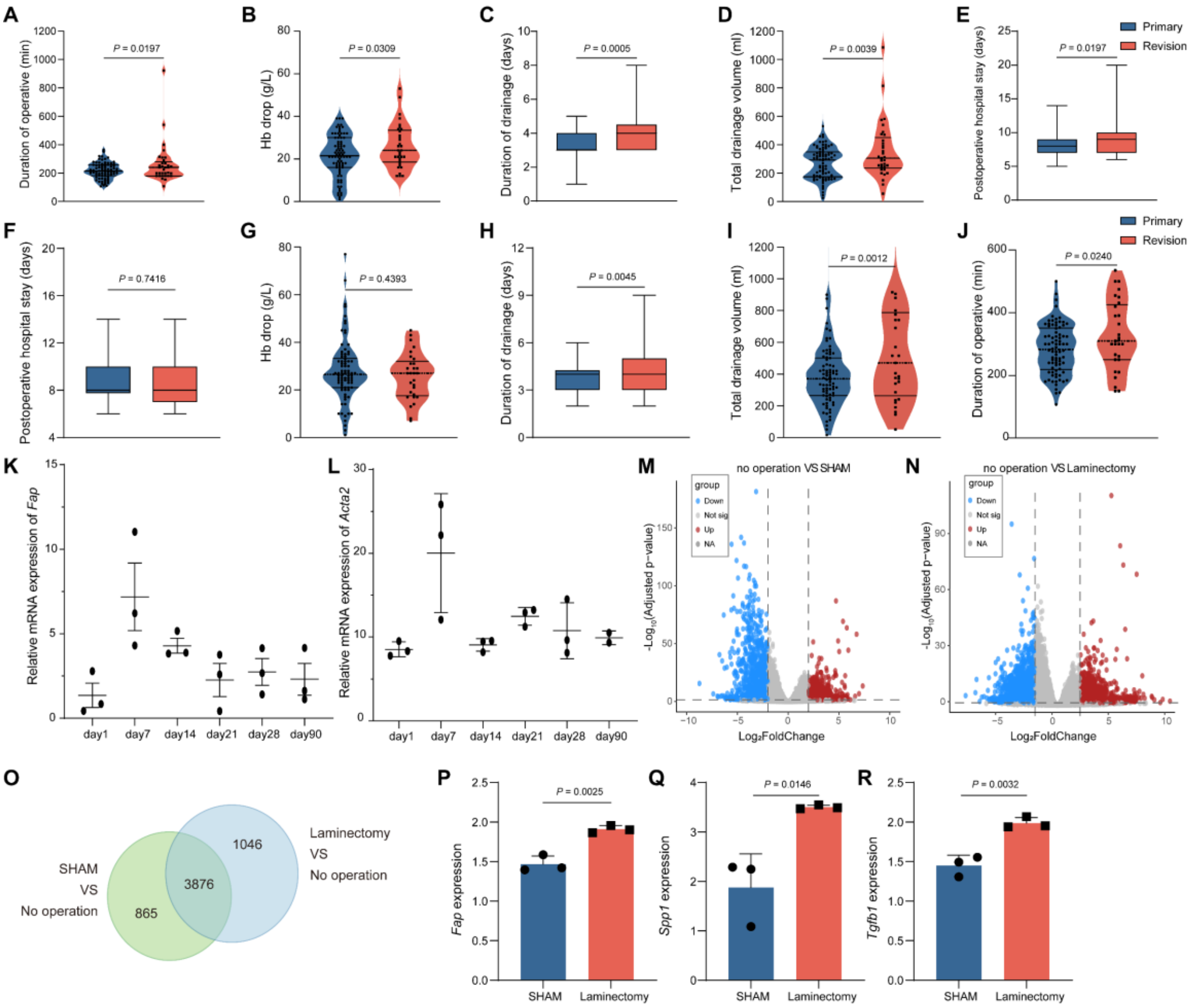

Supplementary Fig. 1. Clinical and molecular features of postoperative epidural fibrosis. (A-E) Clinical characteristics and outcome comparisons between patients undergoing primary versus revision lumbar surgery involving a single segment (Primary, n = 82 patients; Revision, n = 33 patients). Box limits indicate interquartile range (IQR; 25th to 75th percentile); center lines denote median; whiskers extend to minima and maxima within 1.5 × IQR. **(F-J)** Clinical characteristics of primary and revision surgery in patients with multi-segment involvement. Data are from a clinical cohort (Primary, n = 63 patients; Revision, n = 29 patients). Box limits IQR (25th to 75th percentile); center lines denote median; whiskers extend to minima and maxima within 1.5 × IQR. **(K-L)** Time-course qPCR analysis of *Fap* and *Acta2* (encoding α-SMA) mRNA expression at the injury site in a mouse laminectomy model (n = 3 mice per time point). **(M-N)** Volcano plots of differentially expressed genes identified by bulk RNA-seq analysis of mouse laminectomy scar tissue at day 7 (n = 3 mice per group). Red points, significantly upregulated; blue points, significantly downregulated (adjusted $P < 0.05$, $|\log_2$ fold change$| > 0.5$). **(O)** Venn diagram comparing DEGs between SHAM vs. naïve (left) and laminectomy vs. naïve (right) mouse groups. Overlapping region indicates genes common to both surgical conditions. **(P-R)** Bulk RNA-seq analysis of mouse scar tissue showing *Fap*, *Spp1*, and *Tgfb1* mRNA levels in naïve, SHAM, and laminectomy groups (n = 3 mice per group). Data are presented as mean ± SEM. Statistical comparisons between two groups were performed using two-tailed unpaired Student's *t*-test. Source data are provided as a Source Data file.

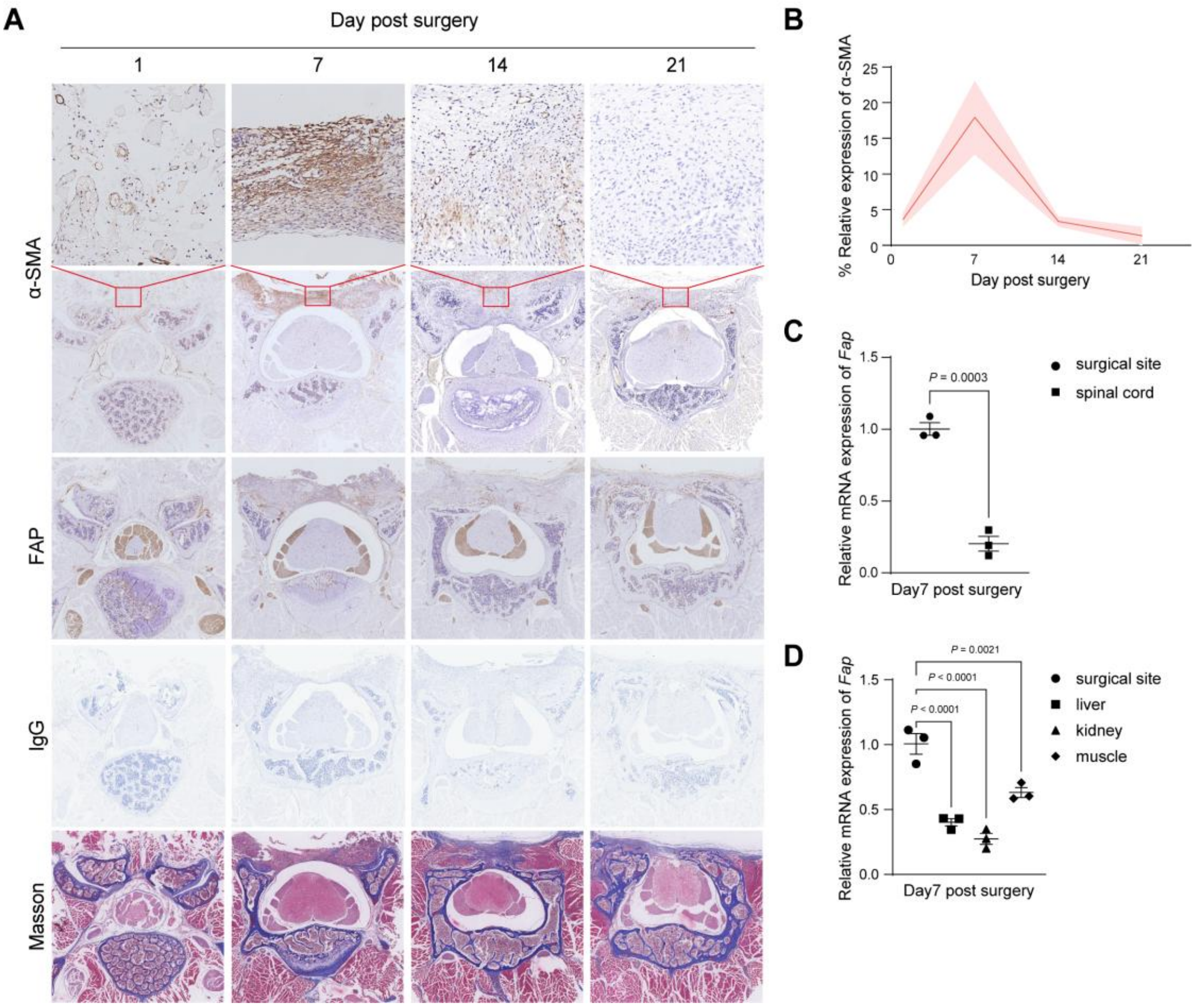

Supplementary Fig. 2. Histological and molecular characterization of the laminectomy site. **(A)** Representative immunohistochemistry and Masson's trichrome staining of the surgical site at postoperative days 1, 7, 14, and 21. Top two rows: α-SMA IHC at 40× and 10× magnification. Third row: FAP IHC at 10× magnification. Fourth row: Isotype control staining (rat IgG) confirming specificity of FAP detection. Bottom row: Masson's trichrome staining at 10× magnification. Images are representative of n = 3 mice per time point. **(B)** Quantification of α-SMA-positive area from IHC images in (A). **(C–D)** qPCR analysis of Fap mRNA expression in the spinal cord, liver, kidney, and muscle at postoperative day 7 relative to postoperative sites (n = 3 mice per group). No significant increase in Fap expression was detected in the spinal cord, liver, kidney, and muscle. Data are presented as mean ± SEM. Statistical comparisons between two groups were performed using two-tailed unpaired Student's *t*-test. Source data are provided as a Source Data file.

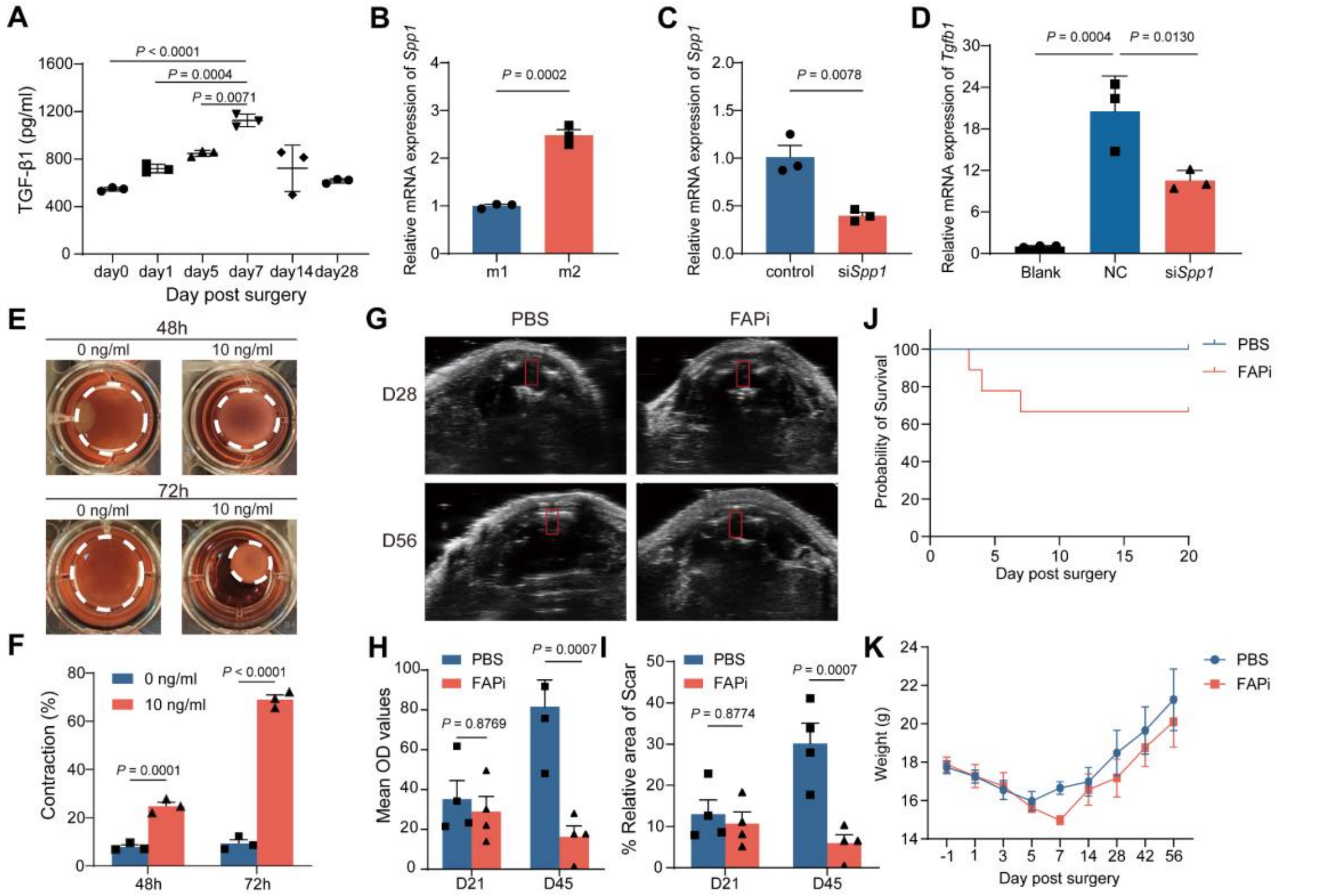

Supplementary Fig. 3. Validation of SPP1-TGF-β1-FAP axis in EF models. (A) Serum TGF-β1 levels measured by ELISA at days 0, 1, 5, 7, 14, and 28 after laminectomy (n = 3 mice per group). **(B)** qPCR analysis of *Spp1* expression in M1 versus M2 polarized (n = 3 biological replicates per group). **(C)** qPCR validation of *Spp1* silencing efficiency in M2 macrophages after siRNA transfection (n = 3 biological replicates per group). **(D)** qPCR analysis of *Tgfb1* expression in M2 macrophages following *Spp1* knockdown (n = 3 biological replicates per group). **(E-F)** Collagen gel contraction assay evaluating NIH/3T3 fibroblast contractility in the presence of TGF-β1 with or without FAPi treatment (n = 3 biological replicates per group). **(G-I)** B-scan ultrasound assessment of epidural scar formation and dural adhesion at 56 days post-laminectomy (n = 4 mice per group). **(J-K)** Kaplan-Meier survival curve (J, n = 9 mice per group) and body weight changes (K, n = 4 mice per group) in FAPi-treated and PBS-treated mice. FAPi treatment was associated with transient body weight loss and reduced survival. Data are presented as mean ± SEM. Statistical comparisons between two groups were performed using two-tailed unpaired Student's *t*-test. Source data are provided as a Source Data file.

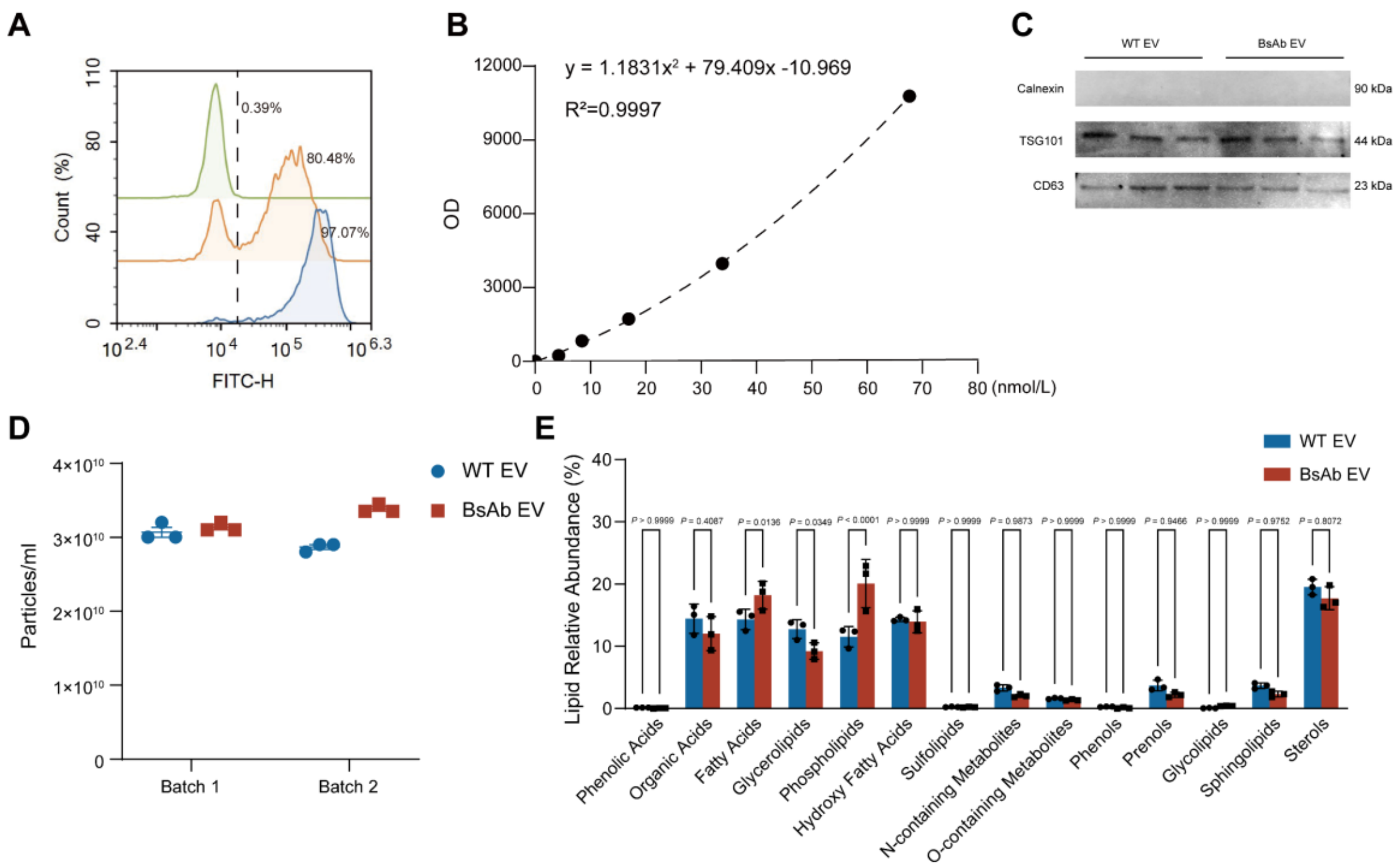

Supplementary Fig. 4. Characterization of FAP×CD3 BsAb EVs producer cells and purified vesicles. (A) Flow cytometry validation of GFP expression on the membrane surface of producer NIH/3T3 cells. Green, wild-type NIH/3T3 cells; yellow, pre-sorted producer cells; blue, post-sorted producer cells. **(B)** Quantification of GFP-tagged BsAb molecule copy number per EV particle. Copy number was calculated as (GFP-tagged BsAb molar concentration × Avogadro's number) / EV particle concentration. Data from three independent EV batches are shown (Batch 1: 820.3 nmol/L, 3.45×10^{10} particles/mL; Batch 2: 819.6 nmol/L, 3.20×10^{10} particles/mL; Batch 3: 819.5 nmol/L, 3.10×10^{10} particles/mL). Mean ± SD = 1.51 ± 0.08 × 10^4 molecules per EV particle. n = 3 independent batches. **(C)** Immunoblot analysis of EVs markers Calnexin, TSG101, and CD63 in WT EVs and BsAb EV preparations. **(D)** Particle counts of two independent EV batches for both WT and BsAb EV, measured by nanoparticle tracking analysis (n = 3 biological replicates per batch). No significant difference was observed between groups. **(E)** Pairwise comparison of lipid class composition between WT EVs and BsAb EV, as determined by untargeted lipidomic profiling (n = 3 biological replicates per group). Data are presented as mean ± SEM except **(B)**. Statistical comparisons between two groups were performed using two-tailed unpaired Student's *t*-test. Source data are provided as a Source Data file.

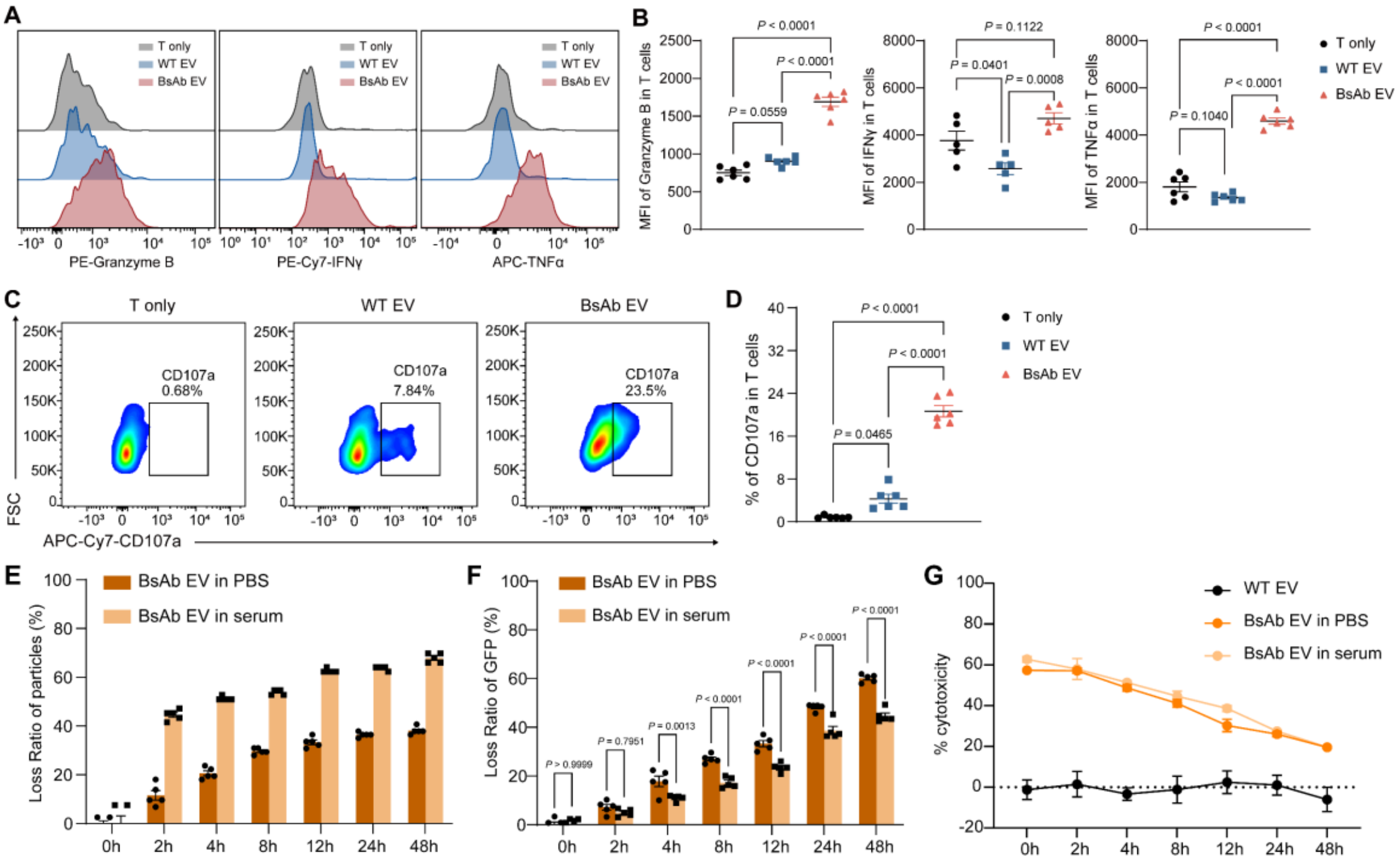

Supplementary Fig. 5. Functional stability and cytotoxic validation of FAP×CD3 BsAb EVs in vitro. (A-B) Representative overlaid flow cytometry histograms (A) and quantification (B) of Granzyme B, IFN-γ, and TNF-α expression in $CD3^{+}CD8^{+}$ T cells following 24 h co-culture with BsAb EV (Granzyme B, n = 6 biological replicates per group; IFN-γ, n = 5 biological replicates per group; TNF-α, n = 6 biological replicates per group). **(C-D)** Representative overlaid density plots (C) and quantification (D) of CD107a expression in $CD3^{+}CD8^{+}$ T cells following 24 h co-culture with BsAb EV (n = 6 biological replicates per group). **(E-G)** Functional stability assessment of FAP×CD3 BsAb EVs incubated in PBS or mouse serum under light-protected conditions at room temperature for 0–48 h. **(E)** Loss rate of GFP fluorescence intensity over time. **(F)** Vesicle particle concentration measured by nanoparticle tracking analysis. **(G)** T cell-mediated cytotoxicity against target cells at an effector-to-target ratio of 10:1 following addition of 50 µL of the EVs mixture at each time point. Quantification is based on 5 technical replicates per group. Data are presented as mean ± SEM. Statistical comparisons between two groups were performed using two-tailed unpaired Student's *t*-test. Source data are provided as a Source Data file.

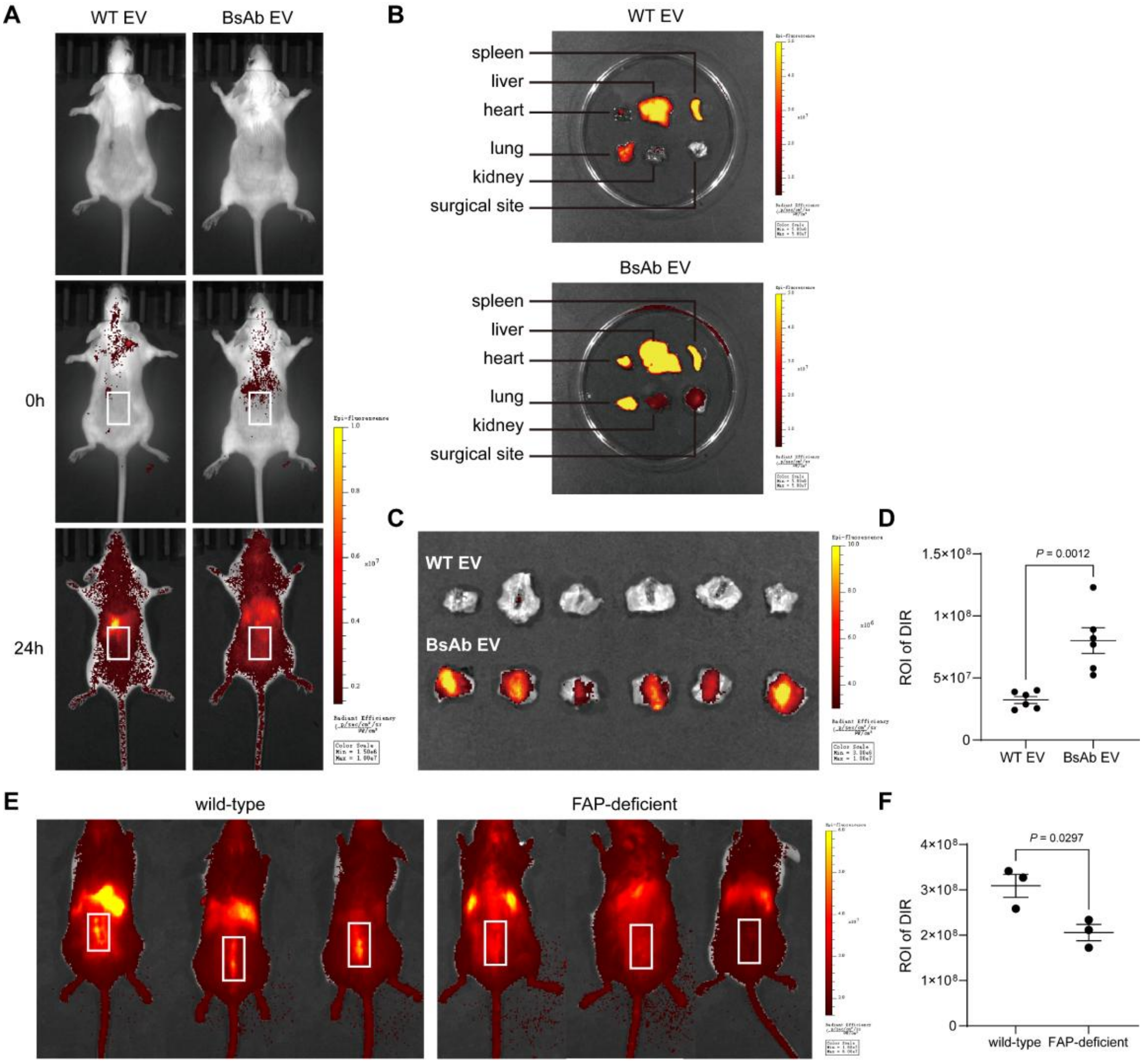

Supplementary Fig. 6. In vivo distribution and targeting of FAP BsAb EVs in a mouse model of laminectomy. (A) Representative whole-body fluorescence images of mice at 0 and 24 h after intravenous injection of DiR-labeled WT EVs and BsAb EVs administered at postoperative day 7. **(B)** Representative ex vivo DiR fluorescence images of major solid organs and laminectomy site tissue harvested at 24 h post-injection from the indicated treatment groups (n = 6 mice per group). **(C)** Close-up DiR fluorescence images of the laminectomy site tissue from each group (n = 6 mice per group). **(D)** Quantitative analysis of region of interest (ROI) fluorescence intensity in the surgical tissue at 24 h post-injection (n = 6 mice per group). **(E)** Representative whole-body fluorescence images of wild-type and FAP-deficient mice at 24 h after intravenous injection of DiR-labeled BsAb EVs administered at postoperative day 7 (n = 3 mice per group). **(F)** Quantitative analysis of ROI fluorescence intensity in the surgical tissue of WT and *Fap* KO mice at 24 h post-injection (n = 3 mice per group). Data are presented as mean ± SEM. Statistical comparisons between two groups were performed using two-tailed unpaired Student's *t*-test. Source data are provided as a Source Data file.

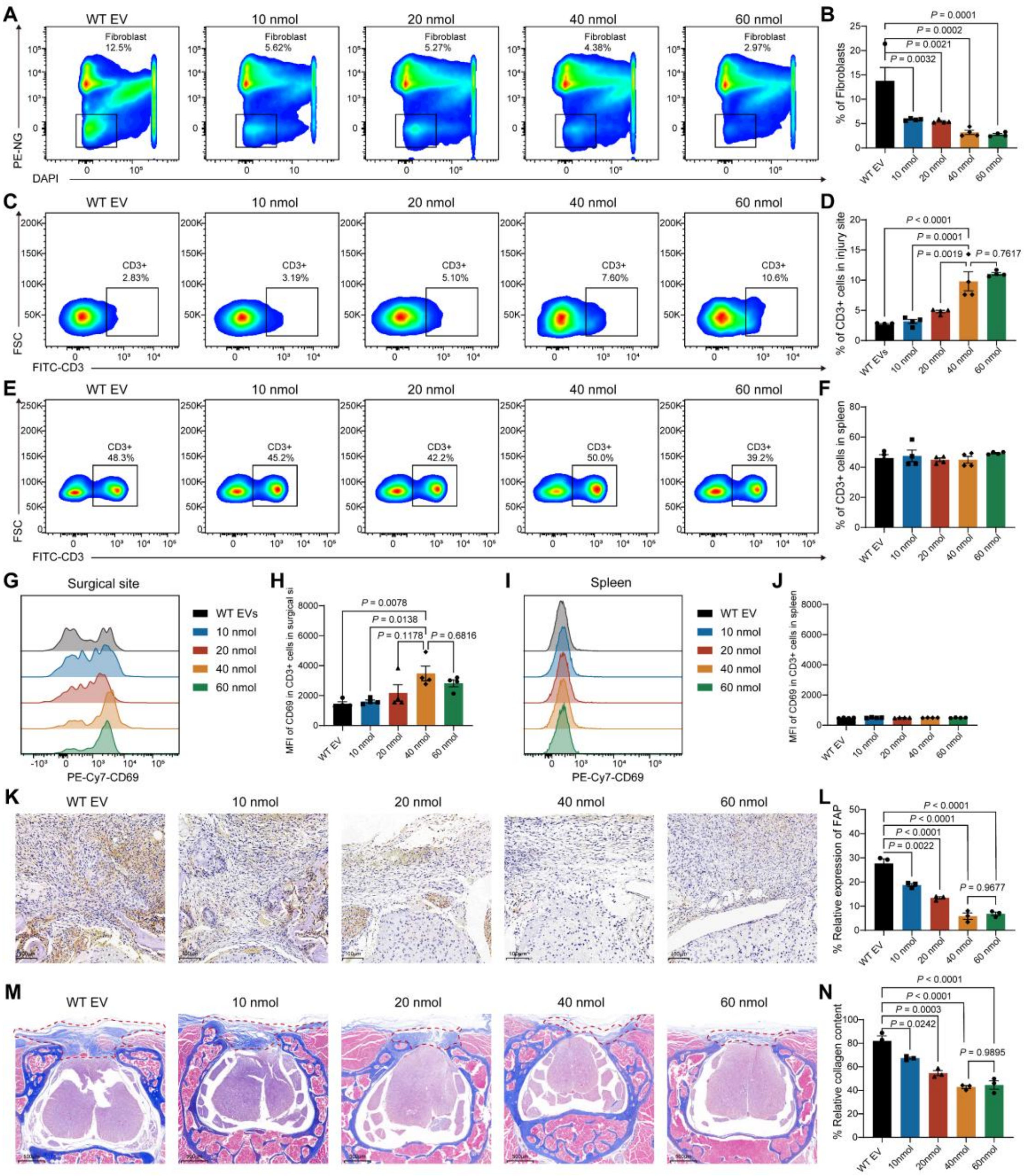

Supplementary Fig. 7. Dose-dependent effects of BsAb EV treatment on fibroblast and T cell populations in vivo. **(A-B)** Representative flow cytometry density plots (A) and quantification (B) of fibroblasts in the injured tissue at postoperative day 7 following treatment with WT EVs or BsAb EVs at 10, 20, 40, or 60 nmol (n = 4 mice per group). **(C-D)** Representative flow cytometry density plots (C) and quantification (D) of $CD3^+$ T cells in the injured tissue from the indicated treatment groups (n = 4 mice per group). **(E-F)** Representative flow cytometry density plots (E) and quantification (F) of $CD3^+$ T cells in the spleen from the indicated treatment groups (n = 4 mice per group). **(G-H)** Representative overlaid flow cytometry histograms (G) and mean fluorescence intensity (MFI) quantification (H) of CD69 expression on $CD3^+$ T cells from the injured tissue (n = 4 mice per group). **(I-J)** Representative overlaid flow cytometry histograms (I) and MFI quantification (J) of CD69 expression on $CD3^+$ T cells from the spleen (n = 4 mice per group). **(K-L)** Representative FAP immunohistochemistry images of the injured tissue at postoperative day 7 (K) and

quantification of FAP-positive area per field (L) (n = 3 mice per group, Scale bar, 100 μm). **(M-N)** Representative Masson's trichrome staining of the injured tissue at postoperative day 56 (M) and quantification of collagen-positive area per field (N) (n = 3 mice per group, Scale bar, 100 μm). Data are presented as mean ± SEM. Statistical comparisons between two groups were performed using two-tailed unpaired Student's *t*-test. Source data are provided as a Source Data file.

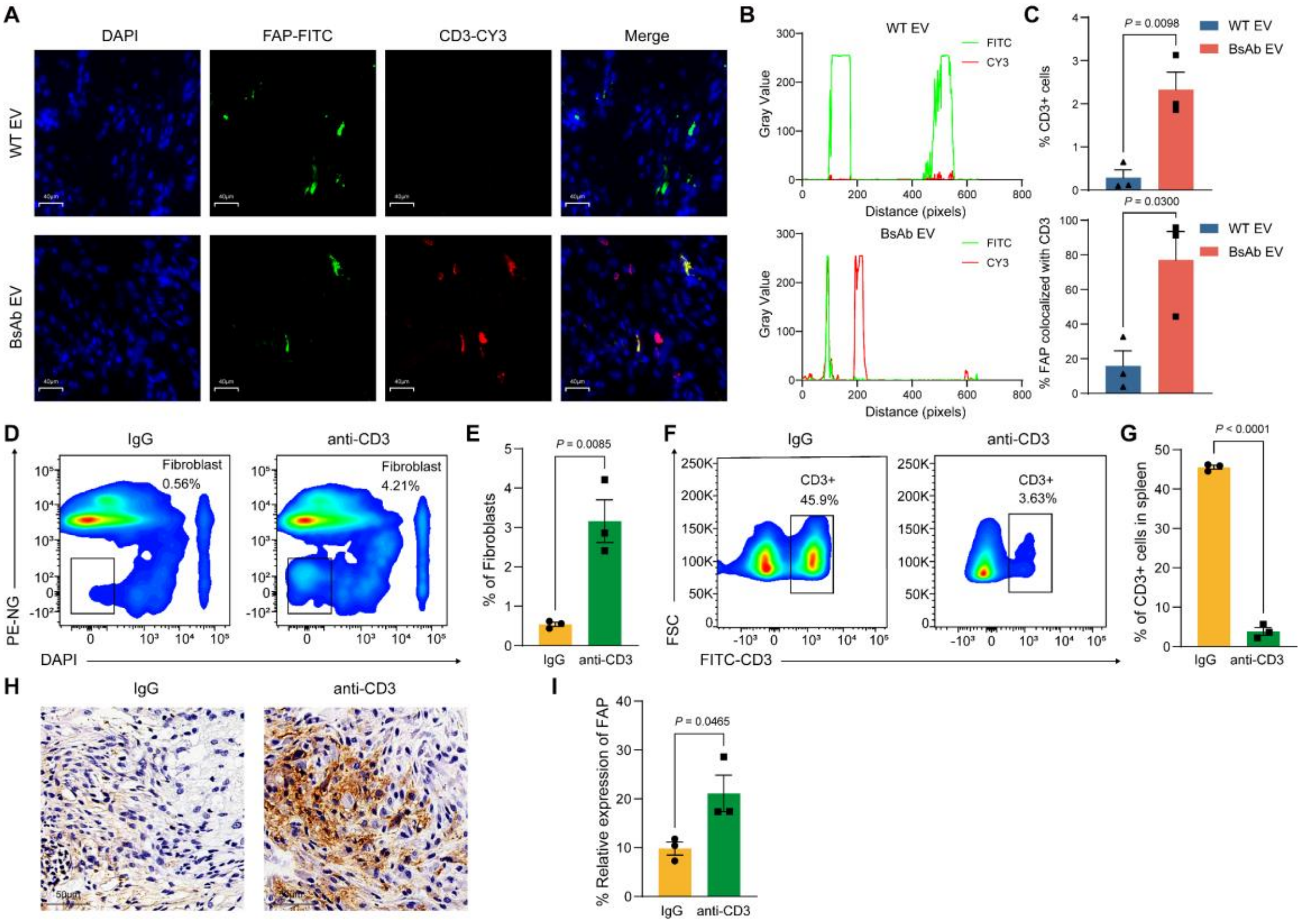

Supplementary Fig. 8. Spatial relationship between FAP⁺ fibroblasts and CD3⁺ T cells and the effect of T cell depletion on BsAb EVs efficacy. **(A-C)** Dual-color immunofluorescence analysis of the laminectomy scar tissue at postoperative day 14 following intravenous injection of WT EVs or BsAb EVs (40 nmol). **(A)** Representative images showing FAP (FITC, green) and CD3 (Cy3, red) staining (n = 3 mice per group, Scale bar, 40 μm). **(B)** Fluorescence colocalization profiles (line scan) of FITC and Cy3 signals along the indicated regions in (A). **(C)** Top: Percentage of $CD3^+$ T cells in the scar tissue. Bottom: Percentage of FAP^+ area colocalized with $CD3^+$ T cells. n = 3 mice per group. **(D-I)** Effect of T cell depletion on BsAb EVs therapeutic efficacy. Mice were pretreated with anti-CD3 monoclonal antibody or control IgG prior to BsAb EV treatment. **(D-E)** Representative flow cytometry density plots (D) and quantification (E) of fibroblasts in the injured tissue at postoperative day 7 (n = 3 mice per group). **(F-G)** Representative flow cytometry density plots (F) and quantification (G) of $CD3^+$ T cells in the spleen confirming successful T cells depletion (n = 3 mice per group). **(H-I)** Representative FAP immunohistochemistry images of the injured tissue at postoperative day 7 (H) and quantification of FAP-positive area per field (I) (n = 3 mice per group; scale bar, 100 μm). Data are presented as mean ± SEM. Statistical comparisons between two groups were performed using two-tailed unpaired Student's *t*-test. Source data are provided as a Source Data file.

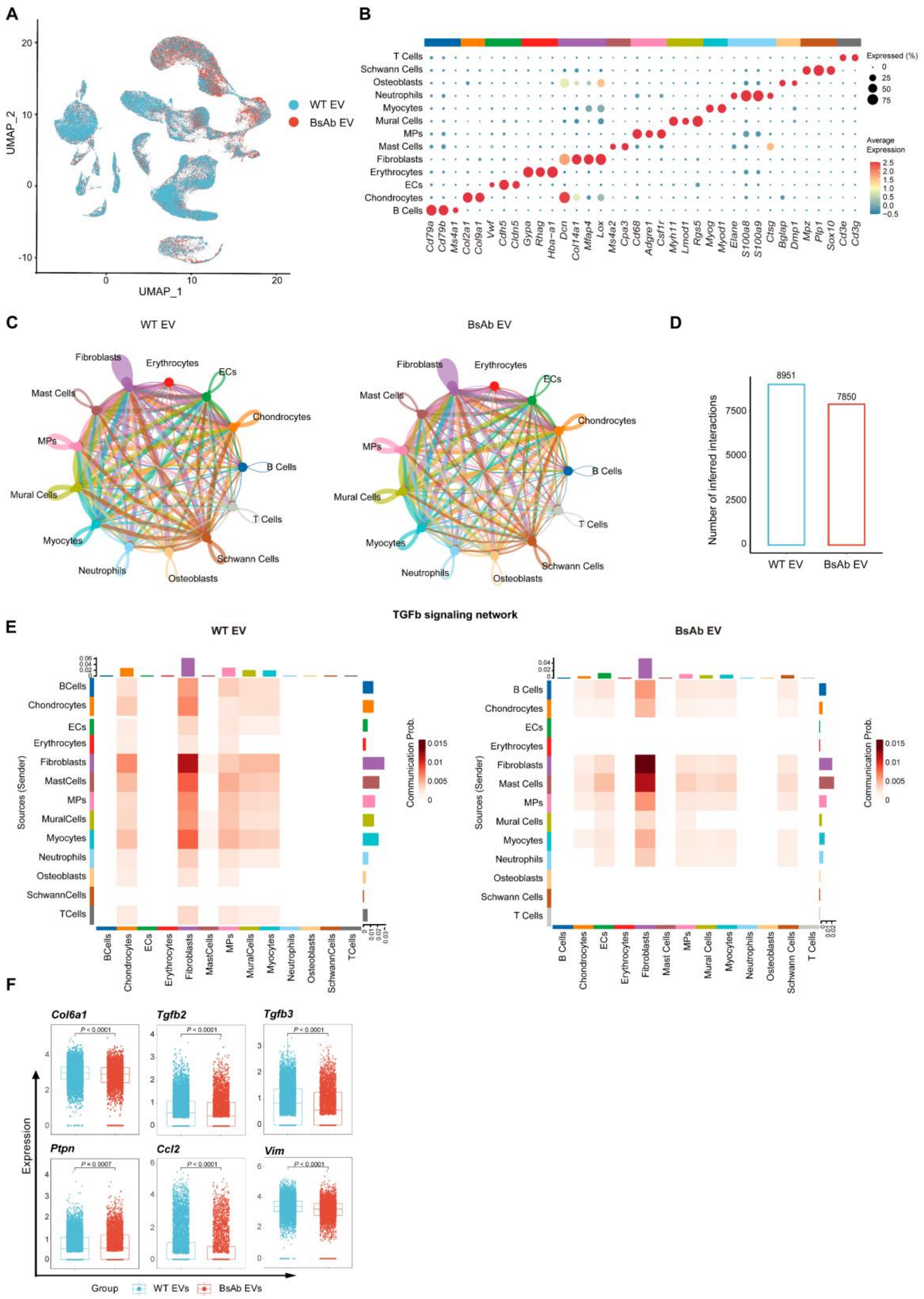

Supplementary Fig. 9. Global landscape of cell types and intercellular communication in postoperative fibrotic lesions following WT EVs or BsAb EV treatment. (A) UMAP plot showing cells from WT EV-treated (blue) and BsAb EV-treated (red) fibrotic lesions collected at postoperative day 7. **(B)** Dot plot illustrating representative marker genes for each identified cell population. Dot size indicates the proportion of expressing cells; color intensity reflects average expression levels (blue, low; red, high). **(C)** Inferred intercellular communication

network among 13 major cell types. Edge thickness reflects the number of ligand-receptor interactions. **(D)** Bar plot quantifying the total number of cell-cell interactions in WT EV- and BsAb EV-treated groups. **(E)** Heatmap showing the number of ligand-receptor pairs between cell type pairs. Rows represent ligand-expressing cells; columns represent receptor-expressing cells. Color intensity corresponds to interaction counts (white, low; red, high). **(F)** Expression levels of fibrosis-related genes in WT EV- and BsAb EV-treated fibrotic lesions. Box limits IQR (25th to 75th percentile); whiskers extend to minima and maxima within 1.5 × IQR (WT EV, n = 7555 cells from 3 biological replicates; BsAb EV, n = 3780 cells from 3 biological replicates). Data are presented as mean ± SEM. Statistical comparisons between two groups were performed using two-tailed unpaired Student's *t*-test. Source data are provided as a Source Data file.

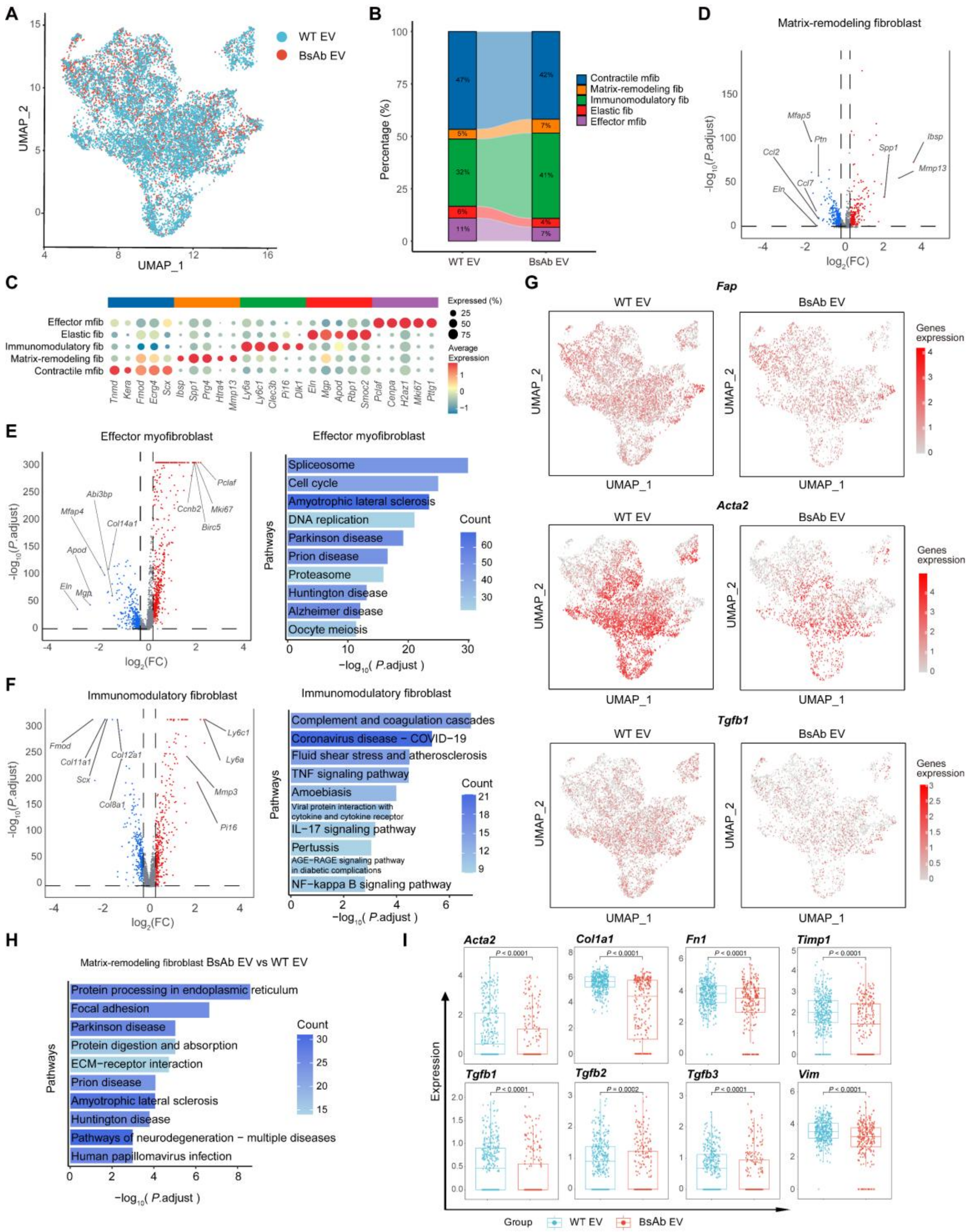

Supplementary Fig. 10. Transcriptional characterization of fibroblast subpopulations in WT EV- and BsAb EV-treated postoperative fibrotic lesions. (A) UMAP plot showing fibroblasts from WT EV-treated (blue) and BsAb EV-treated (red) fibrotic lesions collected at postoperative day 7. **(B)** Frequency of each fibroblast subtype in WT EV- and BsAb EV-treated fibrotic lesions. **(C)** Dot plot showing representative marker genes for each fibroblast population. Dot size indicates the proportion of expressing cells; color intensity reflects average expression levels (blue, low; red, high). **(D)** Volcano plot displaying differentially expressed genes between matrix-remodeling fibroblasts and the other four fibroblast subtypes. Red points,

significantly upregulated; blue points, significantly downregulated (adjusted $P < 0.05$, $|\log_2$ fold change$| > 0.25$). **(E)** Left: Volcano plot showing DEGs between effector myofibroblasts and the other four fibroblast subtypes. Right: Top ten signaling pathways enriched in effector myofibroblasts. **(F)** Left: Volcano plot showing DEGs between immunomodulatory fibroblasts and the other four fibroblast subtypes. Right: Top ten signaling pathways enriched in immunomodulatory fibroblasts. **(G)** UMAP plot showing expression of *Fap*, *Acta2*, and *Tgfb1* in fibroblasts from WT EV- and BsAb EV-treated lesions. **(H)** Top ten signaling pathways associated with genes enriched in matrix-remodeling fibroblasts from BsAb EV-treated lesions. **(I)** Expression levels of fibrosis-related genes in matrix-remodeling fibroblasts from WT EV- and BsAb EV-treated groups. Box limits IQR (25th to 75th percentile); whiskers extend to minima and maxima within 1.5 × IQR (WT EV, n = 350 cells from 3 biological replicates; BsAb EV, n = 248 cells from 3 biological replicates). Data are presented as mean ± SEM. Statistical comparisons between two groups were performed using two-tailed unpaired Student's *t*-test. Source data are provided as a Source Data file.

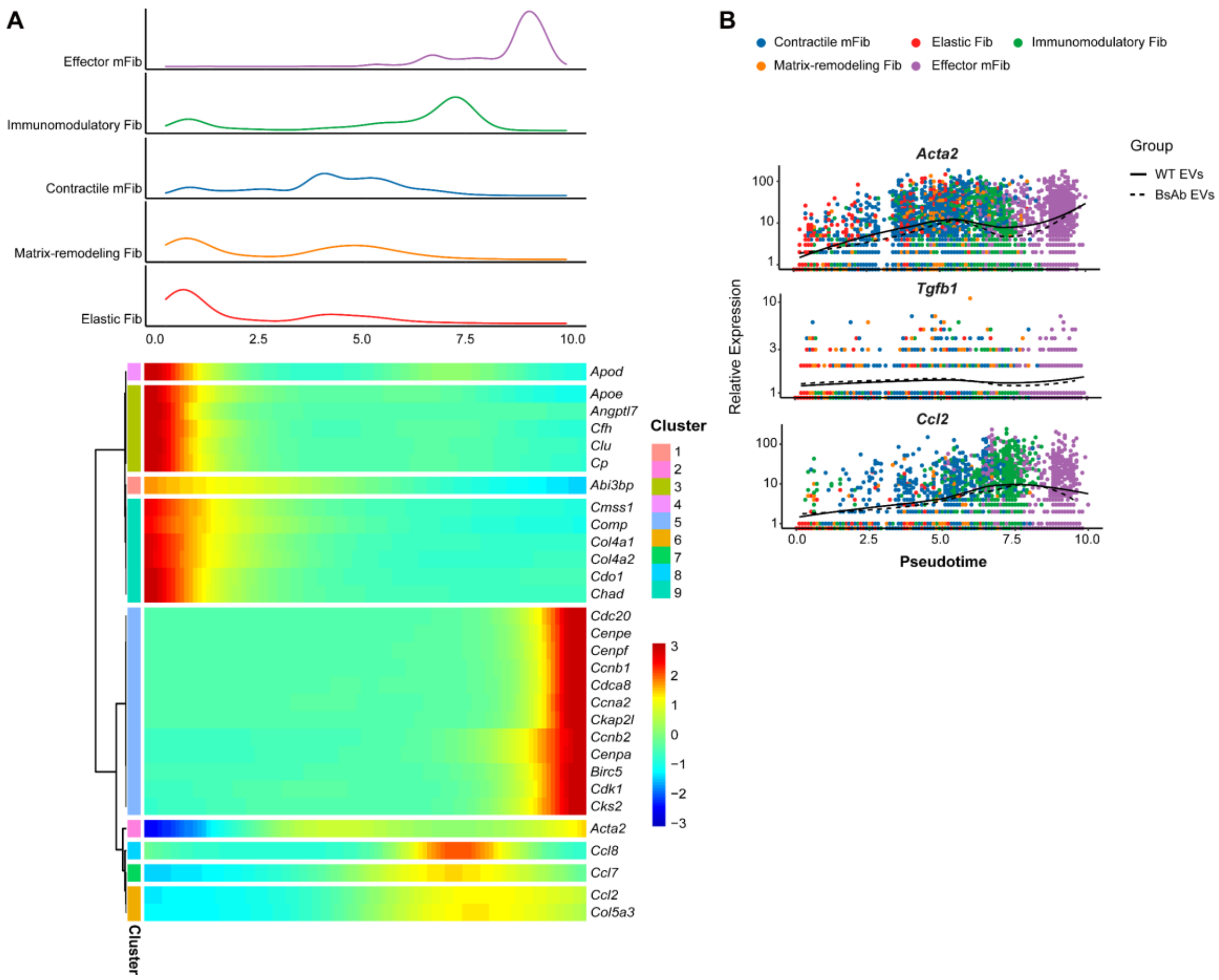

Supplementary Fig. 11. Pseudotime analysis reveals transcriptional dynamics of fibroblasts in WT EVs and BsAb EVs treated lesions. (A) Pseudotime trajectory of fibroblasts from five clusters (upper panel) and heatmap showing dynamic gene expression patterns along pseudotime (lower panel). Representative genes for each trajectory branch are shown on the right. Color scale in heatmap represents Z-score of normalized expression (blue, low; red, high). **(B)** Expression dynamics of *Acta2*, *Tgfb1*, and *Ccl2* along pseudotime in fibroblasts from WT EV- and BsAb EV-treated groups. Source data are provided as a Source Data file.

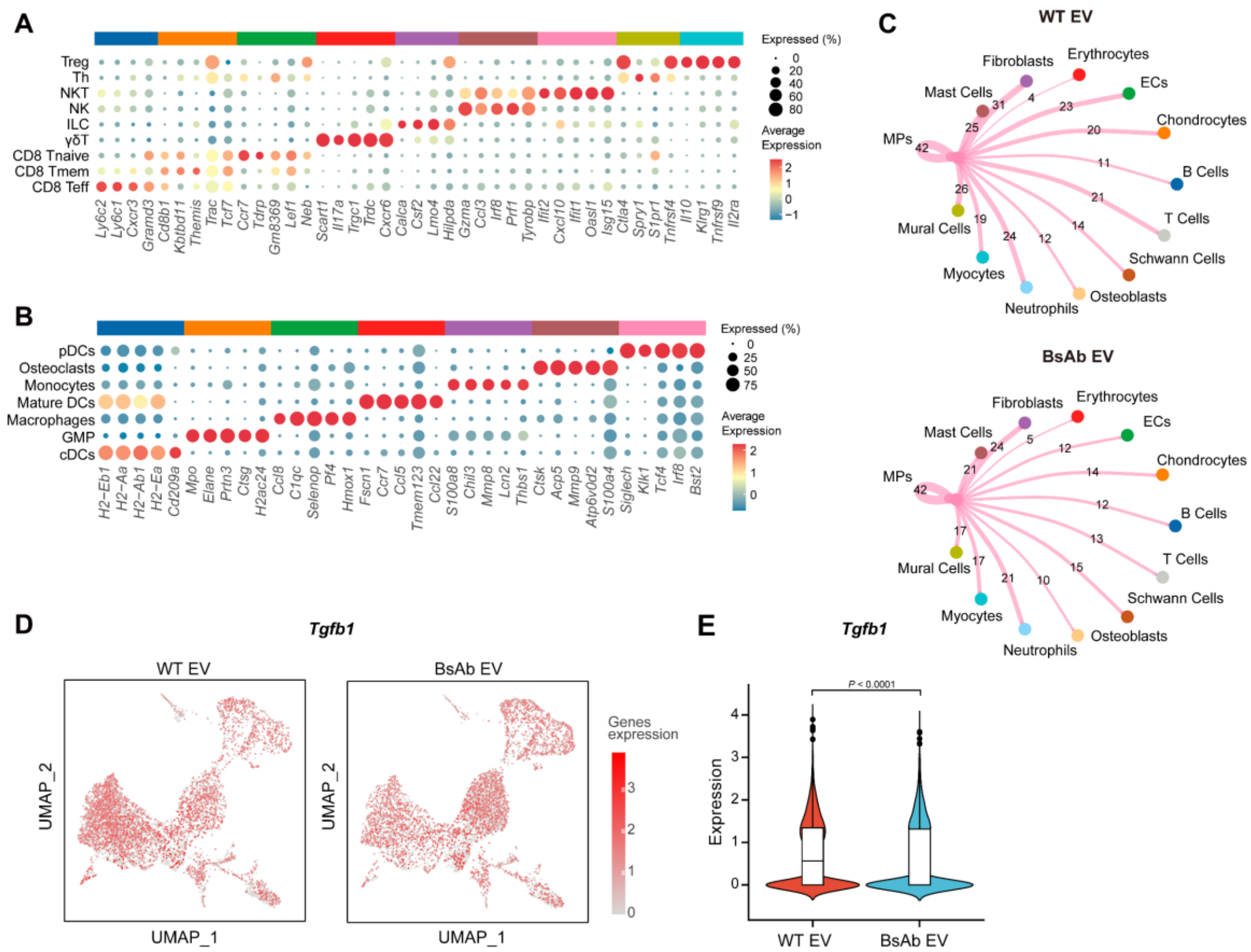

Supplementary Fig. 12. Characterization of marker expression and intercellular communication in T/NK cells and MPs under BsAb EV treatment. (A) Dot plot showing representative marker genes for each T/NK cell population. Dot size indicates the proportion of expressing cells; color intensity reflects average expression levels (blue, low; red, high). **(B)** Dot plot showing representative marker genes for each MP population. Dot size and color intensity are as described in (A). **(C)** Network view of cell-cell communication within MPs and across the 13 major cell types. Edge thickness reflects the number of ligand-receptor interactions. **(D)** UMAP plot showing *Tgfb1* expression in MPs from WT EV- and BsAb EV-treated groups. **(E)** Violin plot illustrating *Tgfb1* transcript levels in MPs from WT EV- and BsAb EV-treated groups. Embedded box plots denote median (center line), interquartile range (box limits, 25th to75th percentile), and 1.5 × IQR (whiskers). WT EV, n = 8996 cells from 3 biological replicates; BsAb EV, n = 8808 cells from 3 biological replicates. Data are presented as mean ± SEM. Statistical comparisons between two groups were performed using two-tailed unpaired Student's *t*-test. Source data are provided as a Source Data file.

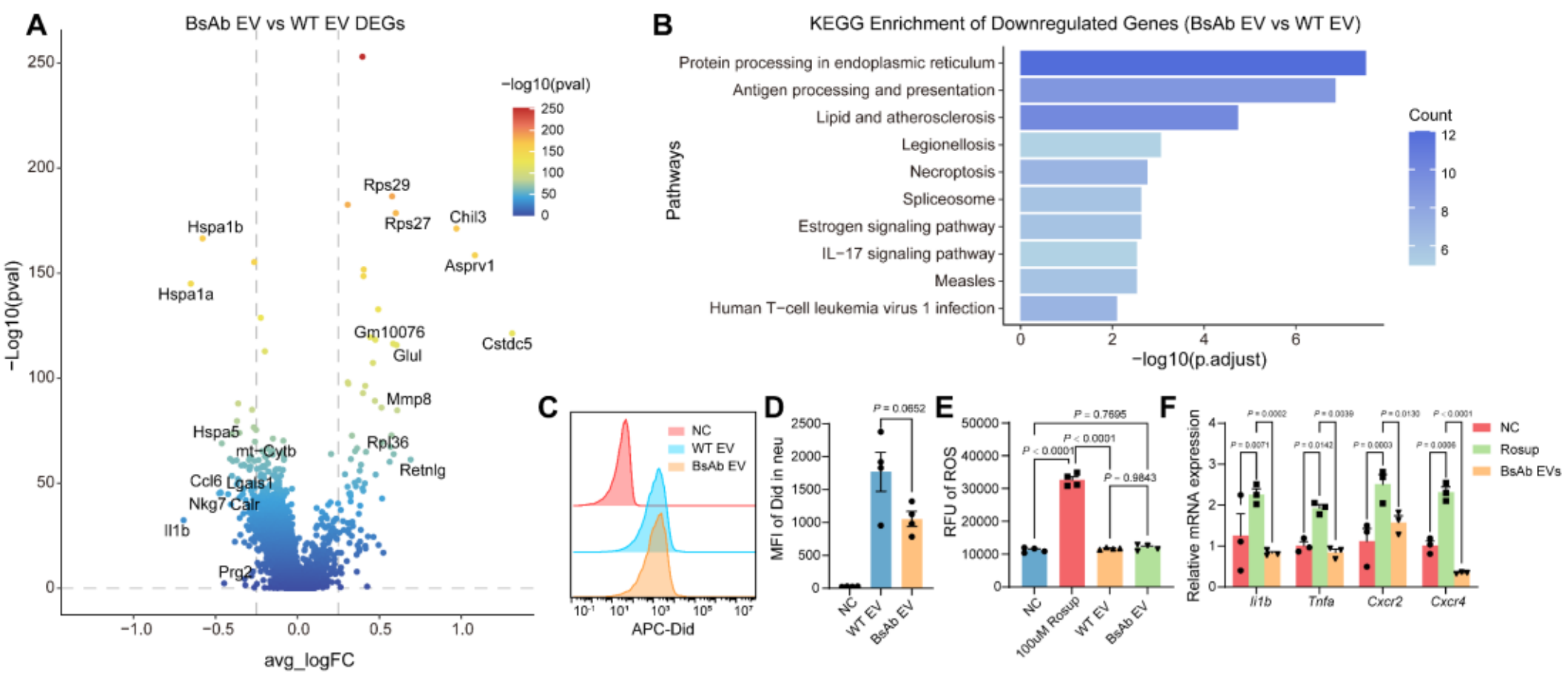

Supplementary Fig. 13. Functional characterization of neutrophils in BsAb EV-treated fibrotic lesions. (A) Volcano plot showing differentially expressed genes in neutrophils from BsAb EV-treated versus WT EV-treated fibrotic lesions at postoperative day 7 (adjusted $P <$ 0.05, $|\log_2$ fold change$| > 0.25$). **(B)** KEGG pathway enrichment analysis of genes downregulated in neutrophils from BsAb EV-treated lesions relative to WT EV-treated lesions. **(C-D)** Representative overlaid flow cytometry histograms (C) and quantification (D) of DiD-labeled EV uptake by neutrophils following co-incubation with WT EVs or BsAb EV. No significant difference in uptake was observed between groups (n = 4 per group). **(E)** Reactive oxygen species (ROS) production in neutrophils following co-incubation with WT EVs or BsAb EV, measured by microplate reader. Rosup (100 μM) was used as a positive control (n = 4 per group). **(F)** qPCR analysis of *Il1b*, *Tnfa*, *Cxcr2*, and *Cxcr4* mRNA levels in neutrophils following co-incubation with WT EVs or BsAb EV. Rosup (100 μM) was used as a positive control (n = 3 per group). Data are presented as mean ± SEM. Statistical comparisons between two groups were performed using two-tailed unpaired Student's *t*-test. Source data are provided as a Source Data file.

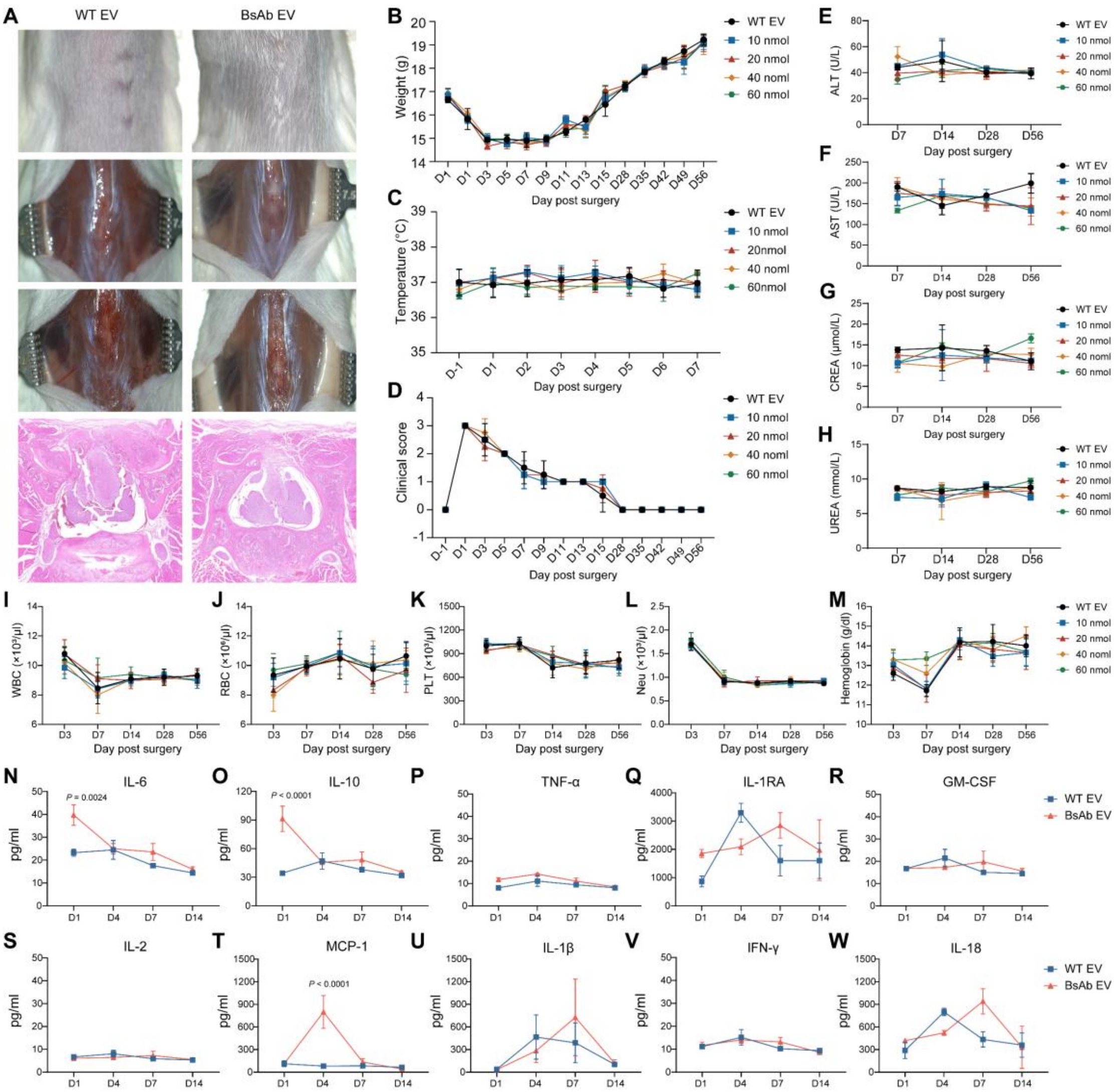

Supplementary Fig. 14. Systemic safety assessment following BsAb EV treatment. (A) Top: Macroscopic view of cutaneous and epidural scarring in each group at 14 days after laminectomy. Bottom: Representative H&E-stained sections of the injured tissue collected from both groups at 56 days post-surgery (Scale bar, 100 μm). **(B-D)** Body temperature (B), body weight change (C), and behavioral score (D) monitored over the 56-day experimental period (n = 4 mice per group). **(E-H)** Serum levels of alanine aminotransferase (ALT), aspartate aminotransferase (AST), blood urea nitrogen (BUN), and creatinine (CR) measured at postoperative days 7, 14, 27, and 56 (n = 3 mice per group). **(I-M)** Complete blood count parameters including white blood cells (I), red blood cells (J), hemoglobin (K), platelets (L), and neutrophils (M) evaluated at postoperative days 7, 14, 27, and 56 (n = 4 mice per group). **(N-W)** Time-course line graphs of serum cytokine levels corresponding to the heatmap shown in Figure 7M. Cytokines were measured at postoperative days 1, 4, 7, and 14 in WT EV- and BsAb EV-treated mice (n = 3 mice per group). Data are presented as mean ± SEM. Statistical comparisons between two groups were performed using two-tailed unpaired Student's *t*-test. Source data are provided as a Source Data file.

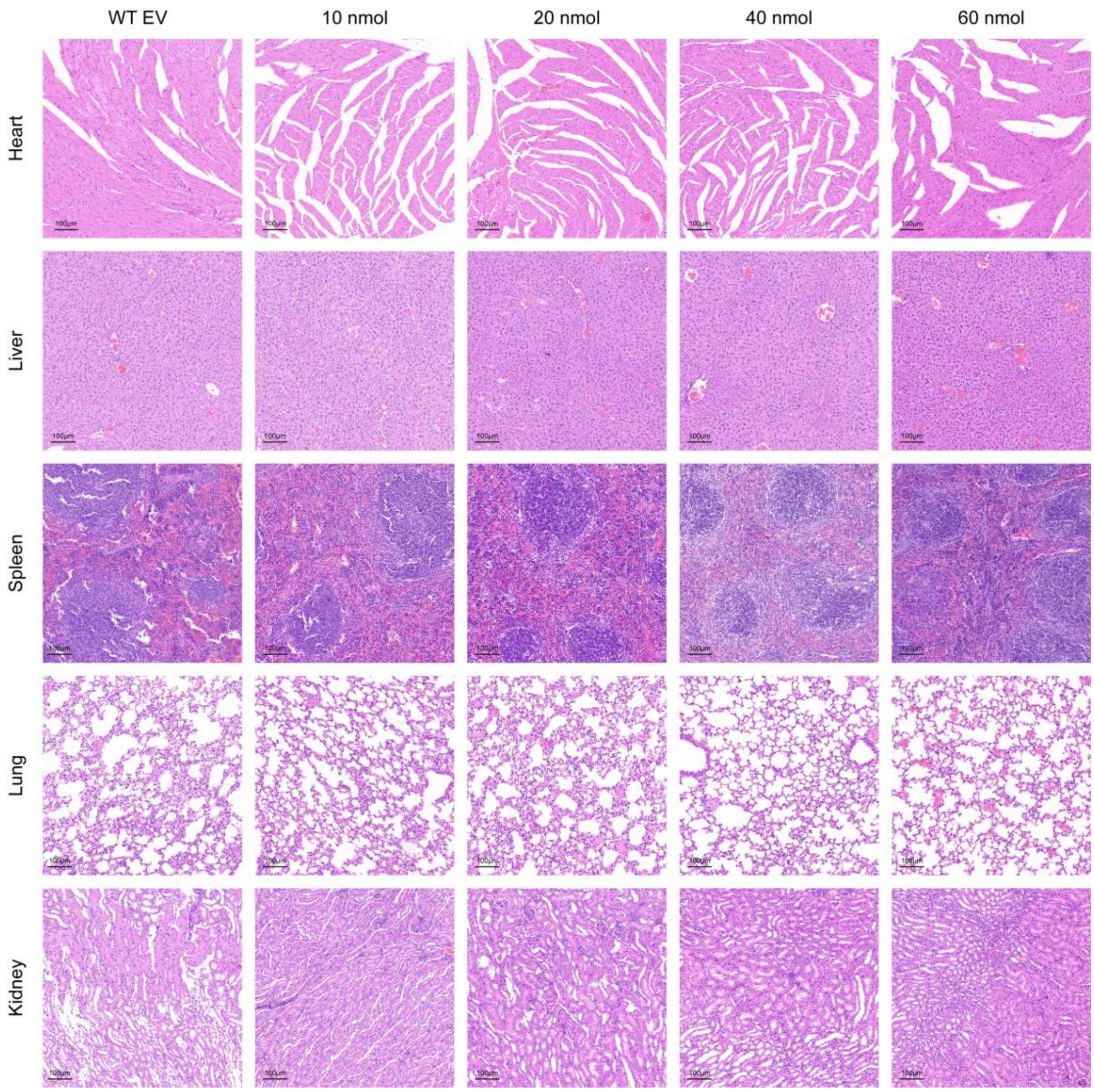

Supplementary Fig. 15. Histological evaluation of major organs following BsAb EV treatment. Representative hematoxylin and eosin (H&E) staining of major solid organs including heart, liver, spleen, lung, and kidney harvested at postoperative day 56 from mice treated with the indicated doses of BsAb EVs or WT EV. No histological evidence of organ damage was observed in any treatment group (Scale bar, 100 μm). Source data are provided as a Source Data file.

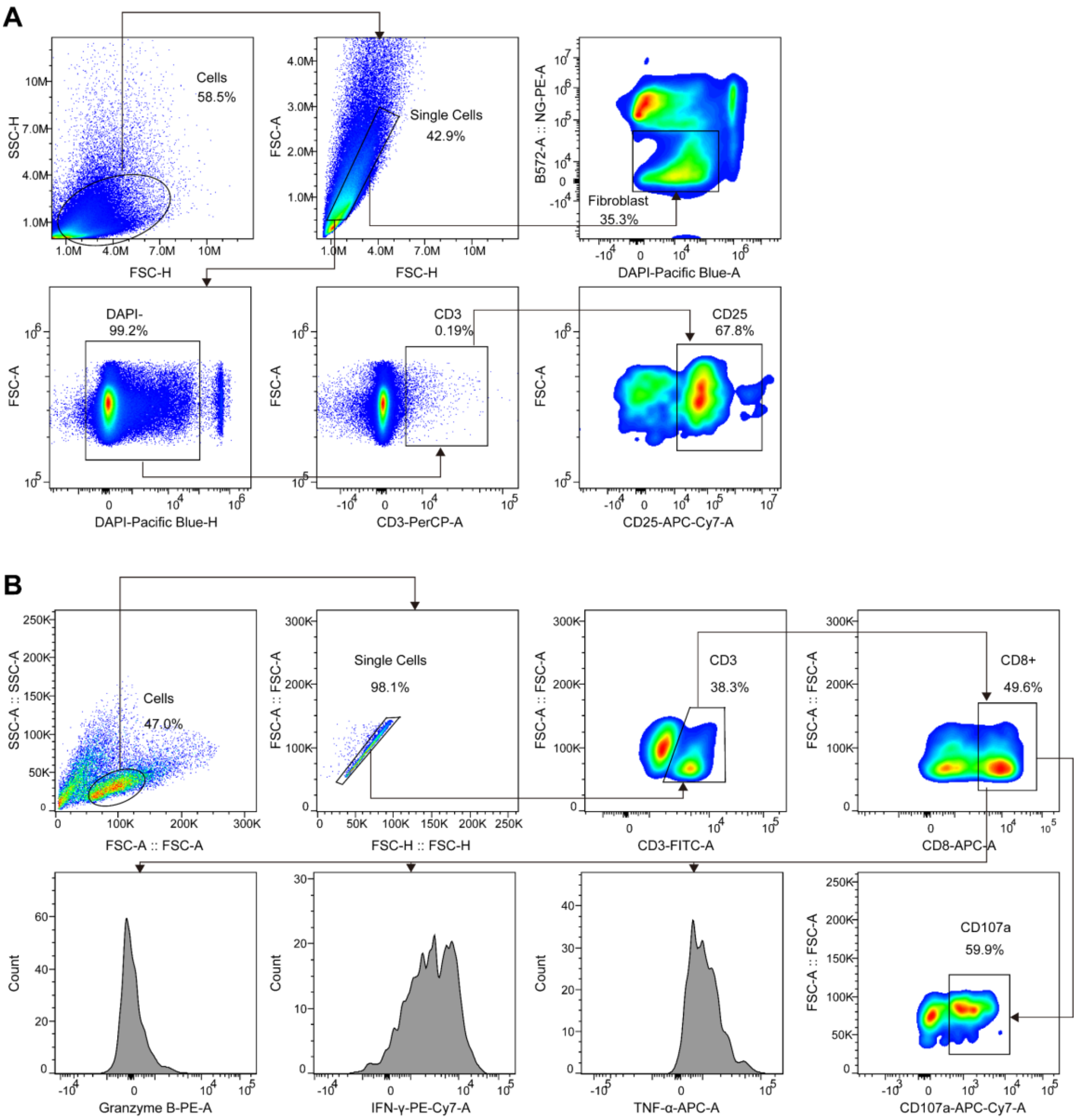

Supplementary Fig. 16. Flow cytometry gating strategies. (A) Representative gating strategy for flow cytometric analysis of cells isolated from mouse tissue. Fibroblasts were identified as PE-lineage (CD31, CD45, Tie2, Ter119, EpCAM)-negative and DAPI-negative cells. Activated T cells were identified as $CD3^+CD25^+$ cells. **(B)** Representative gating strategy for in vitro flow cytometric analysis of co-cultured cells. Cells were gated on FSC/SSC to exclude debris, followed by single cell discrimination. Source data are provided as a Source Data file.

Supplementary Table 1. The clinical characteristics of non-extensive and extensive surgeries.

	single-segment		multi-segment	
	primary	revision	primary	revision
n	82	33	63	29
Sex, male	27 (32.92%)	19 (57.58%)	20 (31.75%)	15 (51.72%)
Age, median years (range)	62 (57, 68)	61 (54, 67)	63 (58, 69)	64 (60, 72)
Postoperative infection, n (%)	1 (1.22%)	3 (9.09%)	0 (0.00%)	1 (3.44%)
Neurological symptoms, n (%)	0 (0.00%)	1 (3.03%)	1 (1.59%)	0 (0.00%)
Muscle weakness, n (%)	2 (2.44%)	1 (3.03%)	1 (1.59%)	2 (6.88%)
Dural tears, n (%)	0 (0.00%)	3 (9.09%)	3 (4.76%)	6 (20.69%)

Supplementary Table 2. Clinical score evaluation.

Score	Movement	Fur	Temperature
0	Moving	Smooth	Normal
1	Low mobility	Slight bristly	
2	Prostrate	Bristly	Cold

Supplementary Table 3. RT-qPCR primer sequences

Gene	Forward Sequence	Reverse Sequence
FAPα	AGTTGAGGACCAGCTCACAG	CATAACCTCCGTAGGACCAGC
ACTA2	CCGGGAGAAAATGACTCAAA	GCGTCCAGAGGCATAGAGAG
SPP1	AGCAAGAAACTCTTCCAAGCAA	GTGAGATTCGTCAGATTCATCCG
COL1A1	GCTCCTCTTAGGGGCCACT	CCACGTCTCACCATTGGGG
COL3A1	CTGTAACATGGAAACTGGGGAAA	CCATAGCTGAACTGAAAACCACC
GAPDH	CAGTGGCAAAGTGGAGATTGTTG	TCGCTCCTGGAAGATGGTGAT
