# Supplementary material for: Bispecific antibody engineered extracellular vesicles redirect T cells to prevent postoperative epidural fibrosis
Source: Nat Commun. 2026 Jun 27;17:8054. doi: 10.1038/s41467-026-74883-3 (PMC13454492; doi:10.1038/s41467-026-74883-3)
Supplement: Supplementary file 2 [file 41467_2026_74883_MOESM2_ESM.pdf]

nature portfolio

Corresponding author(s): Zheng Zhang

Last updated by author(s): Jun 8, 2026

Reporting Summary

Nature Portfolio wishes to improve the reproducibility of the work that we publish. This form provides structure for consistency and transparency in reporting. For further information on Nature Portfolio policies, see our Editorial Policies and the Editorial Policy Checklist.

Statistics

For all statistical analyses, confirm that the following items are present in the figure legend, table legend, main text, or Methods section.

n/a	Confirmed	
☐	☒	The exact sample size (*n*) for each experimental group/condition, given as a discrete number and unit of measurement
☐	☒	A statement on whether measurements were taken from distinct samples or whether the same sample was measured repeatedly
☐	☒	The statistical test(s) used AND whether they are one- or two-sided<br>*Only common tests should be described solely by name; describe more complex techniques in the Methods section.*
☒	☐	A description of all covariates tested
☒	☐	A description of any assumptions or corrections, such as tests of normality and adjustment for multiple comparisons
☐	☒	A full description of the statistical parameters including central tendency (e.g. means) or other basic estimates (e.g. regression coefficient) AND variation (e.g. standard deviation) or associated estimates of uncertainty (e.g. confidence intervals)
☐	☒	For null hypothesis testing, the test statistic (e.g. *F*, *t*, *r*) with confidence intervals, effect sizes, degrees of freedom and *P* value noted<br>*Give P values as exact values whenever suitable.*
☒	☐	For Bayesian analysis, information on the choice of priors and Markov chain Monte Carlo settings
☒	☐	For hierarchical and complex designs, identification of the appropriate level for tests and full reporting of outcomes
☒	☐	Estimates of effect sizes (e.g. Cohen's *d*, Pearson's *r*), indicating how they were calculated

Our web collection on statistics for biologists contains articles on many of the points above.

Software and code

Policy information about availability of computer code

Data collection	Bulk RNA-seq data were generated using the Illumina HiSeq 2000 platform with TruSeq Stranded mRNA library preparation. Single-cell RNA-seq data were generated using the Singleron Matrix® Single Cell Processing System and GEXSCOPE® Single Cell RNA Library Kit and sequenced on an Illumina NovaSeq 6000 platform.
Data analysis	Data analysis used commercial and open-source software, including STAR (v2.6.1b), FeatureCounts (v2.0.1), DESeq2, CeleScope (v3.0.1), Scanpy (v1.9.8), Harmony (v0.0.6), clusterProfiler (v3.16.1), ImageJ (v1.53), and GraphPad Prism (v9). No custom code was used.

For manuscripts utilizing custom algorithms or software that are central to the research but not yet described in published literature, software must be made available to editors and reviewers. We strongly encourage code deposition in a community repository (e.g. GitHub). See the Nature Portfolio guidelines for submitting code & software for further information.

Data

Policy information about availability of data

All manuscripts must include a data availability statement. This statement should provide the following information, where applicable:

- Accession codes, unique identifiers, or web links for publicly available datasets
- A description of any restrictions on data availability
- For clinical datasets or third party data, please ensure that the statement adheres to our policy

The bulk RNA-seq and single-cell RNA-seq data generated in this study have been deposited in the GEO database under accession code GSE310989 [GEO Accession viewer] and GSE310366 [GEO Accession viewer]. The EV lipidomics data generated in this study have been deposited in the Metabolomics Workbench Project ID

PR003148 and Study ID ST004914 [http://dx.doi.org/10.21228/M8S27C] 47.
All other data generated in this study are provided in the Supplementary Information and Source Data file. Materials generated in this study, including plasmids, engineered producer cells and FAP×CD3 BsAb EVs, are available upon reasonable request and may require completion of a Materials Transfer Agreement. Requests should be directed to Z.Z. (zhangzhengtj1992@gmail.com).

Research involving human participants, their data, or biological material

Policy information about studies with human participants or human data. See also policy information about sex, gender (identity/presentation), and sexual orientation and race, ethnicity and racism.

Reporting on sex and gender	Sex was recorded for human participants as male or female. Both male and female participants were included in this study. Sex-specific analyses were not performed, and it is unknown whether the findings are differentially relevant across sexes. Gender identity was not collected or analyzed.
Reporting on race, ethnicity, or other socially relevant groupings	No data on race, ethnicity, or other socially constructed or socially relevant groupings were collected for this study. Therefore, no analyses based on these variables were performed.
Population characteristics	Human participants included two cohorts. The prospective cohort consisted of adult patients who underwent lumbar spinal decompression surgery (open or endoscopic) for lumbar disc herniation, spinal stenosis, or spondylolisthesis. The retrospective cohort included 207 patients who underwent Transforaminal Lumbar Interbody Fusion (TLIF). Age and sex were collected as demographic characteristics. No genotypic information or additional clinical covariates were analyzed beyond surgical diagnosis and procedure type.
Recruitment	Participants in the prospective cohort were recruited from patients scheduled for lumbar spinal decompression surgery at Tongji Hospital. The retrospective cohort included consecutive patients who underwent TLIF during the study period. Participation in the prospective cohort was voluntary and may introduce self-selection bias, whereas the retrospective single-center design may introduce selection bias.
Ethics oversight	Human studies were approved by the Institutional Review Board of Tongji Hospital, Tongji Medical College, Huazhong University of Science and Technology (Approval Nos. TJ-IRB20230770 and TJ-IRB202407058). Written informed consent was obtained from all participants in the prospective cohort.

Note that full information on the approval of the study protocol must also be provided in the manuscript.

Field-specific reporting

Please select the one below that is the best fit for your research. If you are not sure, read the appropriate sections before making your selection.

[x] Life sciences [ ] Behavioural & social sciences [ ] Ecological, evolutionary & environmental sciences

For a reference copy of the document with all sections, see nature.com/documents/nr-reporting-summary-flat.pdf

Life sciences study design

All studies must disclose on these points even when the disclosure is negative.

Sample size	No statistical methods were used to predetermine sample size. For the human studies, the prospective cohort size was limited by feasibility and the availability of eligible patients, whereas the retrospective cohort included all eligible TLIF cases during the study period. For animal and in vitro experiments, sample sizes were based on standard practice in the field and our prior experience.
Data exclusions	No data were excluded from the analyses.
Replication	All major experimental findings were independently replicated. In vitro and in vivo experiments were repeated at least three times unless otherwise indicated. Clinical and imaging cohorts were analyzed as collected.
Randomization	Randomization was not applicable to the human cohorts, as participants were assigned based on clinical eligibility and surgical treatment rather than experimental allocation. All mice were randomly allocated across the treatment groups.
Blinding	Blinding was implemented for imaging and data analysis. For both animal (PET-CT and MRI) and human (PET-CT) imaging studies, the operators performing image acquisition and the investigators analyzing the imaging data were blinded to group allocation and clinical information. For other animal experiments, samples were collected, processed, and analyzed with group identities masked until data analysis to reduce assessment bias. Blinding was not applicable to in vitro experiments due to the nature of the procedures.

Reporting for specific materials, systems and methods

We require information from authors about some types of materials, experimental systems and methods used in many studies. Here, indicate whether each material, system or method listed is relevant to your study. If you are not sure if a list item applies to your research, read the appropriate section before selecting a response.

Materials & experimental systems

n/a	Involved in the study
	x Antibodies
	x Eukaryotic cell lines
x	Palaeontology and archaeology
	x Animals and other organisms
	x Clinical data
x	Dual use research of concern
x	Plants

Methods

n/a	Involved in the study
x	ChIP-seq
	x Flow cytometry
x	MRI-based neuroimaging

Antibodies

Antibodies used	CD31 (clone W18222B, BioLegend, catalog 160203, Rat IgG2b, #)<br>CD45 (clone TEK4, BioLegend, catalog 103105, Rat IgG1)<br>Tie2 (clone 30-F11, BioLegend, catalog 124007, Rat IgG2b)<br>Ter119 (clone TER-119, BioLegend, catalog 116207, Rat IgG2b)<br>EpCAM (clone G8.8, BioLegend, catalog 118205, Rat IgG2a)<br>CD3 (clone 17A2, BioLegend, catalog 100220, Rat IgG2b)<br>CD25 (clone 3C7, BioLegend, catalog 101918, Rat IgG2b)<br>CD69 (clone H1.2F3, BioLegend, catalog 104511, Armenian Hamster IgG)<br>Ly6G (clone 1A8, BioLegend, catalog 127607 1, Rat IgG2a, κ)<br>CD11b (clone M1/70, BioLegend, catalog 101205, Rat IgG2b, κ)<br>DAPI (Biolegend, catalog 422801)<br>FAP (Abcam, catalog ab207178, Rat IgG)<br>GFP (Abcam, catalog ab290, Rat IgG)<br>Annexin A1 (Huabio, catalog ER1803-70, Rat IgG)<br>Annexin V (Huabio, catalog ET1702-62, Rat IgG)<br>GAPDH (Proteintech, catalog 60004-1-Ig, Mouse IgG)<br>a-SMA (Abcam, catalog ab5694, Rat IgG)<br>Calnexin (Proteintech, catalog 10427-2-AP, Rabbit IgG)<br>TSG101 (Proteintech, catalog 28283-1-AP, Rabbit IgG)<br>CD63 (Proteintech, catalog 25682-1-AP, Rabbit IgG)<br>CD3 (Proteintech, catalog 17617-1-AP, Rat IgG)
Validation	All primary antibodies used in this study were commercially sourced and had been validated by the manufacturers for the stated species and applications. Validation information is available on the manufacturers' websites. Antibody performance was further supported by expected staining patterns and specificity observed in our experimental results.

Eukaryotic cell lines

Policy information about cell lines and Sex and Gender in Research

Cell line source(s)	The human myeloid leukemia cell line K562 and the mouse fibroblast cell line NIH/3T3 were obtained from the American Type Culture Collection (ATCC, Manassas, VA, USA). No primary human cell lines were used in this study.
Authentication	All cell lines were authenticated by the supplier using Short Tandem Repeat test.
Mycoplasma contamination	All cell lines tested negative for mycoplasma contamination.
Commonly misidentified lines (See ICLAC register)	No commonly misidentified or cross-contaminated cell lines were used in this study.

Animals and other research organisms

Policy information about studies involving animals; ARRIVE guidelines recommended for reporting animal research, and Sex and Gender in Research

Laboratory animals	This study involved laboratory animals. BALB/c mice (Mus musculus, 5–7 weeks old) were used for the in vivo experiments. FAP-deficient mice (FapLacZ/LacZ) on a BALB/c background were used where indicated. Both male and female mice were included in the study. Primary mouse fibroblasts and T cells used for in vitro experiments were isolated from mice as described in the Methods.
Wild animals	The study did not involve wild animals
Reporting on sex	Sex information was collected for both human participants and animals. Both male and female patients were included in the study. Both male and female mice were also included. Sex-specific analyses were not performed, and it is unknown whether the findings are differentially relevant across sexes. Gender identity was not collected.

Field-collected samples	No field-collected samples were used in the study.
Ethics oversight	Human studies were approved by the Institutional Review Board of Tongji Hospital, Tongji Medical College, Huazhong University of Science and Technology (Approval Nos. TJ-IRB20230770 and TJ-IRB202407058, where applicable). Written informed consent was obtained from participants in the prospective cohort. Animal experiments were approved by the Institutional Animal Care and Use Committee of Tongji Medical College, Huazhong University of Science and Technology.

Note that full information on the approval of the study protocol must also be provided in the manuscript.

Clinical data

Policy information about clinical studies
All manuscripts should comply with the ICMJE guidelines for publication of clinical research and a completed CONSORT checklist must be included with all submissions.

Clinical trial registration	This study was observational and did not involve an interventional clinical trial; therefore, clinical trial registration was not applicable. Human studies were reviewed and approved by the Institutional Review Board of Tongji Hospital, Tongji Medical College, Huazhong University of Science and Technology.
Study protocol	The study protocol was available from IRB of Tongji Hospital, Tongji Medical College, Huazhong University of Science and Technology.
Data collection	The prospective cohort comprised patients who underwent postoperative 68Ga-FAPI PET/CT imaging after lumbar spinal decompression. The retrospective cohort included 207 patients who underwent Transforaminal Lumbar Interbody Fusion (TLIF) at our center, with data collected from electronic medical records according to predefined eligibility criteria.
Outcomes	This study only contains observational study but not clinical trials. No pre-specified primary or secondary clinical outcome measures were defined.

Plants

Seed stocks	n/a
Novel plant genotypes	n/a
Authentication	n/a

Flow Cytometry

Plots

Confirm that:

- [x] The axis labels state the marker and fluorochrome used (e.g. CD4-FITC).
- [x] The axis scales are clearly visible. Include numbers along axes only for bottom left plot of group (a 'group' is an analysis of identical markers).
- [x] All plots are contour plots with outliers or pseudocolor plots.
- [x] A numerical value for number of cells or percentage (with statistics) is provided.

Methodology

Sample preparation	Single-cell suspensions from tissues and cultured cells were prepared and washed before staining. Red blood cell lysis was performed for tissue samples. Cells were stained with antibodies according to the manufacturer's instructions. After staining and washing, samples were acquired by flow cytometry.
Instrument	Flow cytometry data were acquired on BD flow cytometer.
Software	FlowJo (version 10)
Cell population abundance	Cell population abundance was quantified by flow cytometry after gating on the relevant live singlet populations. Where applicable, cells were enriched or sorted before downstream assays.
Gating strategy	FSC-SSC and singlets were pre-gated. All debris was excluded.

- [x] Tick this box to confirm that a figure exemplifying the gating strategy is provided in the Supplementary Information.
