# Supplementary material for: Bispecific antibody engineered extracellular vesicles redirect T cells to prevent postoperative epidural fibrosis
Source: Nat Commun. 2026 Jun 27;17:8054. doi: 10.1038/s41467-026-74883-3 (PMC13454492; doi:10.1038/s41467-026-74883-3)
Supplement: Supplementary file 3 [file 41467_2026_74883_MOESM3_ESM.pdf]

nature portfolio

# Peer Review File

# Bispecific Antibody Engineered Extracellular Vesicles Redirect T Cells to Prevent Postoperative Epidural Fibrosis

Corresponding Author: Dr Zheng Zhang

**This file contains all reviewer reports in order by version, followed by all author rebuttals in order by version.**

Version 0:

Reviewer comments:

Reviewer #1

(Remarks to the Author)
This manuscript reports an innovative strategy, identifying $FAP^+$ matrix remodeling fibroblasts as a key pathogenic subtype of epidural fibrosis (EF), with their activation dependent on the "$SPP1^+$ macrophage - TGFβ1-FAP" signaling axis. Based on this, the authors developed a FAP×CD3 BiTE-modified extracellular vesicle (BiTE EV) therapy, which targets and eliminates $FAP^+$ fibroblasts by recruiting endogenous T cells, thereby preventing epidural fibrosis following laminectomy. The research combines imaging, single-cell transcriptomics, and functional analysis to demonstrate effective fibrosis inhibition and favorable biocompatibility. This concept is novel and relevant to translational applications, and the overall data are convincing. However, several key issues regarding data interpretation, methodological clarity, and mechanistic validation should be addressed before publication.
Specific Comments
1. The authors have innovatively combined the BiTE technology with an EV delivery system, overcoming the technical limitations of CAR-T therapy in benign diseases while maintaining both specificity and safety. In the manuscript, the authors have already validated the morphology of EVs (via electron microscopy), particle size (via NTA), zeta potential, and BiTE molecular expression (via GFP quantification). However, it is recommended to further provide proteomic analysis of EVs (such as the expression of signature proteins like CD63, CD81, and TSG101) to directly confirm the EV purity. Additionally, the manuscript does not assess the RNA and lipid composition of the EVs. It is suggested to supplement with characteristic lipid profiling of the EVs (such as Western blot analysis of lipid-related markers or lipidomics analysis of cholesterol/phospholipid ratios) to ensure consistency with unmodified WT EVs, thus clarifying whether the BiTE modification affects the biological properties of the EVs themselves.
2. How efficient and stable is the display of BiTE on the surface of EVs? The manuscript only indirectly reflects the BiTE content through GFP fluorescence quantification, lacking direct detection methods (such as flow cytometry to assess the positive rate of FAP/CD3 scFv on the surface of EVs). After in vivo administration, what is the half-life of BiTE molecules on the surface of EVs? Can they remain stable in the body for a sufficient duration to continuously activate T cells at the surgical site, ensuring the targeted clearance of $FAP^+$ fibroblasts and the rationality of the delivery strategy? Is there a risk of rapid inactivation due to protease degradation? Are there variations in particle size distribution, BiTE loading, and biological activity (such as T cell killing efficiency) between different batches of EVs? These are critical considerations for clinical translation and require supplementary data.
3. The mechanism of targeting specificity of BiTE EVs to the surgical site needs further clarification: in addition to the binding of BiTE to $FAP^+$ fibroblasts, do the EVs themselves exhibit any tissue-targeting propensity (such as the chemotaxis of NIH/3T3-derived EVs to injured tissue)? If the targeting is solely dependent on the BiTE-FAP interaction, why do WT EVs also show some enrichment at the surgical site (as shown in Figure S4)? The influence of non-specific adsorption needs to be

ruled out.
4. Do BiTE EVs induce T cell overactivation or off-target effects (such as attacking cells with low FAP expression in normal tissues)? Could long-term use lead to immune tolerance or autoimmune reactions? The manuscript does not explore the optimization of EVs' delivery method and dosage: currently, systemic administration via tail vein injection is used—could local delivery (such as intraoperative injection) be considered to further enhance targeting and reduce systemic exposure? What is the dose-response relationship at different administration doses? Is there a window for the minimum effective dose and maximum tolerated dose?

5. Most previous studies on epidural fibrosis (EF) have been conducted in rat models. The current study relies solely on a murine laminectomy model, which limits translational relevance. The authors are encouraged to validate the therapeutic efficacy and safety of BiTE EVs in a rat EF model, to confirm whether the observed anti-fibrotic and immunomodulatory effects can be reproduced in a larger and more clinically representative system.
6. While the manuscript demonstrates depletion of FAP⁺ fibroblasts by BiTE EVs through flow cytometry and scRNA-seq, spatial confirmation by histological co-localization (e.g., FAP with CD3) is lacking. Immunofluorescence or spatial transcriptomic validation would strengthen the claim that BiTE EVs specifically eliminate FAP⁺ fibroblasts in situ without affecting neighboring stromal or neural cells.
7. The proposed mechanism—where BiTE EV–mediated elimination of FAP⁺ fibroblasts leads to subsequent immune modulation and fibrosis attenuation—is supported primarily by correlative findings from scRNA-seq and cytokine profiling. To strengthen the mechanistic interpretation, the authors are encouraged to perform functional validation experiments, such as T-cell depletion, FAP blocking, or rescue assays, to directly test whether these downstream immunological changes indeed depend on FAP⁺ fibroblast clearance. Such evidence would provide a more definitive link between fibroblast targeting and immune reprogramming.
8. How does the advantage of BiTE EVs compare to traditional physical barrier therapies (such as biomaterials) and FAP inhibitors (FAPi as discussed in the manuscript)? Are the benefits of BiTE EVs quantifiable, such as the duration of EF relief, the likelihood of reducing revision surgery risks, and cost-effectiveness? It is recommended to include head-to-head comparison experimental data and related discussion to address these points.
9. Why were NIH/3T3 cells chosen as the source for EVs? Compared to EVs derived from clinically commonly used mesenchymal stem cells (MSCs), do NIH/3T3-derived EVs offer superior immunogenicity, targeting ability, and safety? A comparison with EVs from different sources should be included to validate these aspects.

Minor Issues
1. The clinical sample size for PET/CT is small, with only 6 cases currently providing

preliminary validation of the target. It is recommended to include an additional 10-15 patients to analyze the correlation between FAP expression and the severity of EF.
2. The manuscript only assesses short-term liver and kidney functon(56 days) and systemic inflammatory responses, lacking long-term safety data. Will BiTE EVs induce T cell overactivation or off-target effects (such as attacking cells with low FAP expression in normal tissues)? Could long-term use lead to immune tolerance or autoimmune reactions? It is recommended to include data extending beyond 3 months to address these concerns.
3. Figure 1C, correct “per mousel” to “per mouse”.
4. Figure S1 mixes clinical and murine data but does not label source by panel. Please specify the sample origin for each subpanel in the legend.
5. The authors exclusively used female BALB/c mice for the in vivo laminectomy model, but the rationale for this choice is not provided in the manuscript. Given that both strain- and sex-dependent differences can markedly influence immune responses and fibrotic remodeling, it would be important to briefly justify this selection.
6. There is an apparent mismatch between the Results text and the labeling of Figure 2C and Figure S9D-E. Please ensure full consistency among the main text, figure panels, and the legend (including statistics/conditions).
7. At least a brief explanation of the target protein should be provided in the figure legend of Figure 3F, clarifying the rationale for its detection.
8. The abbreviation “MPs” used in Figure 4 is insufficiently defined. The authors should clarify in the legend or Methods that it refers to mononuclear phagocytes to improve clarity and readability.
9. In Figure S5E, the color scales of the heatmaps for WT EVs and BiTE EVs are not unified, which reduces the direct visual comparability between groups. It is recommended to use a consistent color scale or normalized value range across both conditions to ensure accurate visual interpretation of intercellular communication intensity.
10. In the sentence “…genes previously linked to fibroblast activation (Figure 5I–J, Figure S6C–E)”, the figure citation appears to be incorrect. The referenced data correspond to Figure S8C–E, not S6C–E. Please correct the figure numbering accordingly.
11. The figure legend should specify the number of animals used in each group.
12. There is an inconsistency regarding the source of EVs. The Methods section states that BiTE EVs were generated from engineered NIH/3T3 fibroblasts, whereas the Discussion refers to HEK293-derived EVs. This discrepancy should be clarified to ensure consistency and transparency in the description of EV origin.
13. The legend for Figure 3B is not precise. The panel illustrates the design and proposed working principle of FAP×CD3 BiTE EVs, rather than the “mechanism of fibrosis alleviation.”

Reviewer #2

(Remarks to the Author)
The study presents an immunotherapeutic approach using BiTE-engineered extracellular vesicles to target FAP⁺ fibroblasts, however, several critical issues preclude its acceptance in its current form. My major concerns are as follows:

The authors claim that BiTE EVs did not induce systemic toxicity based on serum ALT, AST, BUN, and CR levels at a single early time point (postoperative day 5). However, this limited analysis is inadequate to rule out potential immunotoxicity, especially given the systemic administration of a T cell-redirecting therapy. No data are provided on systemic T cell activation, cytokine release syndrome (CRS), or immune cell infiltration in non-target organs (e.g., lung, liver, spleen). The absence of long-term toxicity data is concerning, particularly given the potential for delayed autoimmune or off-target effects.

Uncontrolled T cell activation in non-target tissues could lead to autoimmune pathology, which was not evaluated.

The study relies heavily on observational and correlative data but falls short in providing direct mechanistic evidence. While $FAP^+$ fibroblasts are implicated in fibrosis, no genetic (e.g., FAP knockout) or lineage-tracing models were used to confirm their necessity in epidural fibrosis. in addition, although BiTE EVs are proposed to recruit T cells, no in vivo T cell depletion or adoptive transfer experiments were performed to confirm that the antifibrotic effect is T cell-dependent. Moreover, the scRNA-seq analysis is descriptive and lacks functional validation.

Regarding characterization of BiTE EVs, functional stability was only assessed up to 48 hours at room temperature. Stability under physiological conditions (e.g., in serum) and in vivo half-life were not evaluated. The composition, cargo, and potential immunogenicity of BiTE EVs were not thoroughly characterized.

In conclusion, the authors must address these fundamental issues through additional in vivo validation, comprehensive toxicity studies, and clearer demonstration of clinical applicability before the work can be considered for publication.

Reviewer #3

(Remarks to the Author)
This study explores the use of bispecific T cell engager-decorated extracellular vesicles (BiTE EVs) targeting FAP and CD3 to mitigate epidural fibrosis (EF) in a murine laminectomy model. While the concept is interesting, the manuscript suffers from several critical weaknesses that undermine the validity and impact of the findings. The study lacks focus, with insufficient evidence to support the proposed mechanism of action of BiTE EVs. The connection between clinical observations and experimental data is tenuous, and key methodological details are missing. The single-cell RNA sequencing data, while extensive, do not convincingly demonstrate the proposed cellular interactions or therapeutic mechanisms.

1. The manuscript posits a link between the technical difficulties of revision spine surgery and epidural fibrosis, but the evidence presented is circumstantial. While clinical data confirm that the revision cohort had more complicated perioperative metrics (e.g., longer operative time, greater blood loss), these findings are only correlative and do not demonstrate that epidural fibrosis was the underlying cause.
2. The MRI and PET/CT imaging data are supportive but do not directly prove mechanistic claims. The use of $^{68}$Ga-FAPI PET/CT imaging in Figure 1A to indicate fibroblast activation is not sufficiently justified. Given the cellular heterogeneity of surgical tissue, the observed radiotracer uptake cannot be definitively attributed to fibroblasts alone. Consequently, the assertion that the signal reflects activated fibroblasts requires further validation.
3. The core claim—that BiTE EVs simultaneously engage $FAP^+$ fibroblasts and T cells to induce targeted cytotoxicity—is not adequately supported. In vitro co-culture experiments are needed to demonstrate that BiTE EVs facilitate T cell–fibroblast conjugation and that cytotoxicity is specifically dependent on both FAP and CD3 engagement (e.g., via blocking antibodies).
4. The possibility that BiTE EVs may directly affect other cell types (e.g., neutrophils) or act through non-specific immune modulation is not ruled out.
5. Key details are missing, such as the method for labeling fibroblasts in flow cytometry (Figure 3N). Figure legends and descriptions are overly simplistic and fail to clarify experimental conditions, statistical methods, or the meaning of key annotations (e.g., red arrows in Figure 1A). Inconsistencies between figure panels and their descriptions (e.g., Figure 2C, 2D) further undermine confidence in the data.
6. The scRNA-seq data reveal changes in cell proportions (e.g., increased neutrophils) but do not establish a direct link between BiTE EV treatment and T cell–mediated fibroblast depletion. The data suggest broader immunomodulatory effects (e.g., on MPs and neutrophils), raising the question of whether the observed benefits are truly due to T cell–mediated cytotoxicity or secondary to other immune changes. The trajectory and communication analyses are descriptive and lack functional validation.
7. The expression of FAP in normal dura mater is not provided as a baseline for injury-induced changes (Figure 1C–E). The influence of BiTE EVs on T cells or fibroblasts in the absence of the other cell type is not tested, leaving open the possibility of off-target or indirect effects.
8. A critical gap in this study is the lack of direct mechanistic evidence demonstrating how BiTE EVs mediate their effects. It is essential to perform reductionist in vitro experiments to deconvolute this interaction. Specifically, the authors should investigate whether BiTE EVs directly activate or modulate T cells in the absence of fibroblasts, and conversely, whether they have any direct, T-cell-independent effects on fibroblast biology. Furthermore, definitive evidence is required to show that BiTE EVs can simultaneously bind both FAP-positive fibroblasts and T cells to form a ternary complex, thereby physically bridging the two cell types to enable targeted cytotoxicity.
9. A more quantitative and direct characterization of the BiTE EVs is critical. First, the average number of anti-FAP/anti-CD3 scFv molecules displayed per EV particle should be quantified, as this directly influences the functional avidity of the EVs. Second, definitive in vitro evidence demonstrating the specific binding of these BiTE EVs to FAP-expressing fibroblasts (compared to FAP-negative controls) is required to substantiate the platform's foundational targeting mechanism.

Version 1:

Reviewer comments:

Reviewer #1

(Remarks to the Author)
I have carefully evaluated the revised manuscript and the authors' response to the previous round of review. Overall, the revised manuscript is substantially improved. The authors have added important experimental data, including FAP-deficient mice, dose-response analysis, in vitro and in vivo T-cell-dependent functional validation, and a more comprehensive safety assessment. These additions significantly strengthen the technical and mechanistic foundation of the study. Nevertheless, several points still require clarification or more cautious wording before publication.

Specific Comments
1. The current data show that BsAb EVs attenuate postoperative fibrotic remodeling when administered during the early postoperative period. However, the study does not demonstrate reversal of established, mature epidural fibrosis. I therefore recommend replacing "reverse" with "attenuate", "mitigate", or "prevent" in the title and throughout the manuscript where appropriate.
2. The clinical revision-surgery cohort and FAPI PET/CT imaging provide useful translational context, but they remain correlative. The imaging cohort includes only a single postoperative time point and lacks direct histological correlation. Moreover, the PET/CT signal cannot be definitively attributed to fibroblasts alone in heterogeneous postoperative tissue. The manuscript should consistently describe the clinical data as supportive evidence of FAP-associated stromal remodeling, rather than direct proof of activated fibroblast accumulation or causal involvement in revision-surgery difficulty.
3. The FAP-KO experiment is an important addition and supports a functional role for FAP in postoperative fibrosis. However, because this is a global FAP-deficient model, it does not exclude contributions from non-fibroblast FAP-expressing stromal compartments or indirect developmental/repair effects. The authors should avoid wording that implies definitive fibroblast-specific genetic causality.
4. The in vitro macrophage–fibroblast experiments support a plausible regulatory mechanism. However, direct in vivo validation of macrophage-derived SPP1/TGF-β1 signaling is still limited. I recommend using language such as "contributes to" or "is associated with" rather than stating that this axis definitively drives EF progression.
5. The expanded safety assessment is reassuring within the tested perioperative mouse model. However, long-term immunogenicity, repeated dosing, autoimmune sequelae, and toxicity against low-level FAP-expressing normal tissues remain unresolved. The phrase "without systemic toxicity" should be revised to "without detectable systemic toxicity under the tested conditions" or similar wording.
6. The lack of rat or larger-animal validation, the use of systemic rather than local delivery, and the use of NIH/3T3-derived EVs remain important limitations. These issues do not necessarily preclude publication as a proof-of-concept study, but they should be clearly acknowledged.
7. The page numbers in the current PDF version are not clearly visible, which makes it difficult to verify the page and line references provided in the response letter. Please ensure that page numbers are clearly displayed in the revised manuscript PDF.
8. The response to Comment 8 should be further incorporated into the Discussion section. Although the authors addressed the comparison between BiTE EVs, conventional physical barrier strategies, and pharmacological FAP inhibition in the response letter, the corresponding discussion does not appear to be sufficiently incorporated into the revised manuscript. Please add a dedicated paragraph in the Discussion section to clarify the translational positioning of BiTE EVs relative to existing anti-epidural fibrosis approaches, including physical barrier materials and FAP inhibitors.
9. The response letter would benefit from directly including the revised text and representative revised figures. To facilitate the reviewers' assessment of the revision, the authors are encouraged to paste the key revised text directly into the response letter, rather than only providing page and line references. When major figure panels or supplementary figures have been added or substantially modified, representative images or cropped figure panels may also be included in the response letter. This would allow reviewers to more clearly and efficiently evaluate how each comment has been addressed.

Reviewer #2

(Remarks to the Author)
The authors have addressed my concerns.

Reviewer #3

(Remarks to the Author)
Most of my concerns have been addressed.

**Open Access** This Peer Review File is licensed under a Creative Commons Attribution 4.0 International License, which permits use, sharing, adaptation, distribution and reproduction in any medium or format, as long as you give appropriate credit to the original author(s) and the source, provide a link to the Creative Commons license, and indicate if changes were made.
In cases where reviewers are anonymous, credit should be given to 'Anonymous Referee' and the source.
The images or other third party material in this Peer Review File are included in the article's Creative Commons license, unless indicated otherwise in a credit line to the material. If material is not included in the article's Creative Commons license and your intended use is not permitted by statutory regulation or exceeds the permitted use, you will need to obtain permission directly from the copyright holder.
To view a copy of this license, visit https://creativecommons.org/licenses/by/4.0/

**Response to Reviewers' Comments**

**Title: Bispecific Antibody Engineered Extracellular Vesicles Redirect T Cells to Reverse Epidural Fibrosis**

We sincerely thank the Reviewers for their careful evaluation of our manuscript and for their thoughtful and constructive comments. In response, we have substantially revised the manuscript and performed additional experiments to address the major concerns raised, including expanded functional validation, safety assessment, and characterization of the BsAb EV platform. All changes in the revised manuscript are highlighted in yellow for ease of reference. Below, we provide a detailed point-by-point response to each comment. For clarity, reviewer comments are shown in italic font, followed by our responses in plain text.

In the revised manuscript, we have also replaced the term “BiTE EV(s)” with “BsAb EV(s)” throughout to use a more general and accurate terminology for the bispecific antibody-engineered EV platform.

**Reviewer #1**

**General Comment:** *This manuscript reports an innovative strategy, identifying FAP⁺ matrix remodeling fibroblasts as a key pathogenic subtype of epidural fibrosis (EF), with their activation dependent on the "SPP1⁺ macrophage - TGFβ1-FAP" signaling axis. Based on this, the authors developed a FAP×CD3 BiTE-modified extracellular vesicle (BiTE EV) therapy, which targets and eliminates FAP⁺ fibroblasts by recruiting endogenous T cells, thereby preventing epidural fibrosis following laminectomy. The research combines imaging, single-cell transcriptomics, and functional analysis to demonstrate effective fibrosis inhibition and favorable biocompatibility. This concept is novel and relevant to translational applications, and the overall data are convincing. However, several key issues regarding data interpretation, methodological clarity, and mechanistic validation should be addressed before publication.*

**Response:** We are grateful to the Reviewer for the positive assessment and for the detailed, constructive feedback. We have carefully considered all of the points raised

and addressed them in detail below.

**Comment 1:** *The authors have innovatively combined the BiTE technology with an EV delivery system, overcoming the technical limitations of CAR-T therapy in benign diseases while maintaining both specificity and safety. In the manuscript, the authors have already validated the morphology of EVs (via electron microscopy), particle size (via NTA), zeta potential, and BiTE molecular expression (via GFP quantification). However, it is recommended to further provide proteomic analysis of EVs (such as the expression of signature proteins like CD63, CD81, and TSG101) to directly confirm the EV purity. Additionally, the manuscript does not assess the RNA and lipid composition of the EVs. It is suggested to supplement with characteristic lipid profiling of the EVs (such as Western blot analysis of lipid-related markers or lipidomics analysis of cholesterol/phospholipid ratios) to ensure consistency with unmodified WT EVs, thus clarifying whether the BiTE modification affects the biological properties of the EVs themselves.*

**Response:** We thank the Reviewer for this important suggestion. We have now performed and included the requested characterization data.

(1) Protein characterization of EV preparations: We supplemented Western blot analyses of canonical EV-associated markers. Both WT EVs and BsAb EVs showed detectable expression of TSG101 and CD63, whereas Calnexin, used as a negative marker for cellular contamination, was undetectable in both preparations. These data support the successful isolation of EV-enriched preparations with minimal contamination from cellular components.

*Location in revised manuscript: Results section, Page 8, Lines 220-224 and Figure S4C.*

(2) Lipid Composition: We performed untargeted lipidomic profiling and found that, relative to WT EVs, BsAb EVs showed a significant increase in the relative abundance of fatty acids and phospholipids, together with a significant decrease in glycerolipids. Consistent with these changes, the cholesterol-to-phospholipid ratio was significantly lower in BsAb EVs than in WT EVs.

*Location in revised manuscript: Results section, Page 8, Lines 225-229, Figure 3G-H*

*and Figure S4E.*

(3) RNA Content: We additionally quantified total RNA content across independent EV batches and found no significant difference in total RNA content between WT EVs and BsAb EVs.

*Location in revised manuscript: Results section, Page 8, Lines 229-230, Figure 3I.*

**Comment 2:** *How efficient and stable is the display of BiTE on the surface of EVs? The manuscript only indirectly reflects the BiTE content through GFP fluorescence quantification, lacking direct detection methods (such as flow cytometry to assess the positive rate of FAP/CD3 scFv on the surface of EVs). After in vivo administration, what is the half-life of BiTE molecules on the surface of EVs? Can they remain stable in the body for a sufficient duration to continuously activate T cells at the surgical site, ensuring the targeted clearance of FAP$^+$ fibroblasts and the rationality of the delivery strategy? Is there a risk of rapid inactivation due to protease degradation? Are there variations in particle size distribution, BiTE loading, and biological activity (such as T cell killing efficiency) between different batches of EVs?*

**Response:** We appreciate these critical questions regarding reproducibility and stability.

(1) Surface display efficiency and stability: We have now added additional evidence supporting efficient surface display of the engineered construct. Specifically, flow-cytometric surface staining under non-permeabilizing conditions showed strong concordance between extracellular staining signal and GFP signal, supporting effective surface presentation of the BsAb construct on EV-associated producer cells. In addition, we estimated the average construct loading to be approximately $1.51 \times 10^4$ GFP-tagged BsAb molecules per EV particle across three independent batches.

*Location in revised manuscript: Results section, Page 8, Lines 231-235, Figure 3J, Figure S4B.*

To address stability, we performed in vitro incubation assays in both PBS and mouse serum. These experiments showed gradual time-dependent loss of GFP-associated signal and particle abundance over 48 h, but BsAb EVs retained detectable T-cell-mediated cytotoxic activity under both conditions throughout this interval.

*Location in revised manuscript: Results section, Page 8, Lines 247-249, Figure S5E–G.*

We agree that direct in vivo pharmacokinetic analysis and precise surface half-life measurement of the BsAb molecules would be informative. However, these were not directly measured in the current study. Instead, our in vivo data show that BsAb EV accumulate at the laminectomy site at 24 h in a FAP-dependent manner (Figure S6A–F), and that treatment induces dose-dependent fibroblast depletion and T-cell activation in vivo (Figure S7A–N), supporting that the platform persists long enough to achieve biologically meaningful local activity. We have framed this point more cautiously in the revised manuscript.

*Location in revised manuscript: Results section, Page 9, Lines 254-255, Figure S6A–F; Page 9, Lines 258-259, Figure S7A–N.*

(2) Batch-to-Batch Consistency: We have now included additional analyses to address reproducibility. NTA measurements showed no significant difference in particle counts between independent WT EVs and BsAb EVs batches (Figure S4D). Construct loading per EV particle was estimated across three independent BsAb EV batches with low inter-batch variability. In addition, functional readouts, including T-cell activation and cytotoxicity assays, were reproduced across independent experiments, supporting the robustness of the production process and the reproducibility of BsAb EVs activity.

*Location in revised manuscript: Results section, Page 8, Lines 224-225, Figure S4D.*

**Comment 3:** *The mechanism of targeting specificity of BiTE EVs to the surgical site needs further clarification: in addition to the binding of BiTE to FAP$^+$ fibroblasts, do the EVs themselves exhibit any tissue-targeting propensity (such as the chemotaxis of NIH/3T3-derived EVs to injured tissue)? If the targeting is solely dependent on the BiTE-FAP interaction, why do WT EVs also show some enrichment at the surgical site (as shown in Figure S4)?*

**Response:** We thank the Reviewer for this important comment regarding targeting specificity. To address the possibility that the EV backbone itself might exhibit nonspecific tropism toward injured tissue, we performed an additional in vivo

biodistribution experiment in Fap knockout mice. Following laminectomy and intravenous administration of DiR-labeled BsAb EVs, robust accumulation at the surgical site was observed in WT mice, whereas this enrichment was markedly diminished in Fap knockout mice (Figure S6E–F). These data support that the enhanced retention of BsAb EVs at the lesion is primarily FAP dependent, rather than being driven solely by an intrinsic tissue-targeting property of the EV backbone.

We agree that WT EVs also showed low-level signal at the injury site. We interpret this signal as limited nonspecific postoperative retention associated with tissue injury and local vascular/permeability changes, rather than active targeting. Importantly, this signal was substantially weaker than that of BsAb EVs and was not associated with comparable fibroblast depletion or antifibrotic activity. Thus, while a modest degree of passive accumulation at injured tissue cannot be excluded, the selective enrichment and biological efficacy of BsAb EVs depend predominantly on the BsAb–FAP interaction.

We also added discussion to clarify that EV properties can be influenced by producer-cell origin, which may contribute to baseline heterogeneity in biodistribution. Accordingly, we now acknowledge in the Discussion that EV source is an important variable that should be further evaluated in future translational studies.

*Location in revised manuscript: Results section, Page 9, Lines 253-254, Figure S6E-F; Discussion section, Page 13, Lines 417-423.*

**Comment 4:** *Do BiTE EVs induce T cell overactivation or off-target effects (such as attacking cells with low FAP expression in normal tissues)? Could long-term use lead to immune tolerance or autoimmune reactions? The manuscript does not explore the optimization of EVs' delivery method and dosage: currently, systemic administration via tail vein injection is used—could local delivery (such as intraoperative injection) be considered to further enhance targeting and reduce systemic exposure? What is the dose-response relationship at different administration doses? Is there a window for the minimum effective dose and maximum tolerated dose?*

**Response:** We thank the Reviewer for these important comments regarding off-target activity, delivery route, and dose optimization. We have now expanded the revised

manuscript with additional safety, cytokine, neutrophil, and dose-response data.

(1) Off-target activation and systemic safety: In vitro coculture experiments showed that BsAb EVs induced only modest T-cell activation in the absence of FAP-positive target cells, whereas substantial cytotoxicity was observed only when both T cells and FAP-positive targets were present (Figure S5A-D). In addition, BsAb EVs did not mediate significant killing of FAP-negative target cells, supporting that BsAb engagement alone is insufficient for full effector function without target-dependent crosslinking (Figure 4E-I).

*Location in revised manuscript: Results section, Page 8, Lines 240-247, Figure 4E-I, Figure S5A-D.*

We further expanded the in vivo safety assessment. Across the tested dose groups, mice showed no overt signs of systemic toxicity, including hyperthermia, weight loss, or behavioral abnormalities. Serum ALT, AST, BUN, creatinine, and complete blood counts remained comparable between control and treatment groups across postoperative days 7, 14, 27, and 56, and no histological evidence of injury was observed in major solid organs at day 56. Multiplex serum cytokine analysis showed only mild and transient increases in IL-6 and IL-10 on postoperative day 1, both remaining below threefold above baseline, with no marked elevation of IL-1RA, IL-1β, or IL-18. Together, these data do not support overt systemic immune overactivation under the tested dosing conditions.

*Location in revised manuscript: Results section, Page 12, Lines 355-374, Figure S14B-W, Figure S15.*

To address the possibility of off-target effects on other immune compartments, we also examined neutrophil responses. Although MCP-1 was elevated on postoperative day 4 and neutrophil abundance increased in the scRNA-seq dataset, in vitro assays did not detect significant direct activation or chemotactic recruitment of neutrophils by BsAb EVs. These findings suggest that the observed neutrophil changes are more likely secondary to local tissue remodeling after fibroblast clearance than a direct off-target effect of BsAb EVs.

*Location in revised manuscript: Results section, Page 11, Lines 329-336, Figure S13A-*

*F.*

(2) Immune tolerance and autoimmune reactions: This study was designed to assess efficacy and safety within a relatively short perioperative treatment window, rather than longer-term immune memory or tolerance. Within this scope, BsAb EVs showed favorable efficacy and safety. Whether prolonged or repeated exposure might lead to immune tolerance or other adaptive immune consequences remains an interesting question for future investigation.

(3) Route of administration: We chose intravenous administration as a stringent and standardized delivery route for proof-of-concept testing in the murine laminectomy model. Repeated local injection in this setting could itself perturb the wound and complicate interpretation of postoperative fibrosis outcomes. Importantly, robust antifibrotic efficacy was observed even with systemic administration. We agree that local or intraoperative delivery may further reduce systemic exposure and could be advantageous in future translational development, and we also discuss this point explicitly in the revised Discussion.

*Location in revised manuscript: Discussion section, Page 13, Lines 392-399.*

(4) Dose-response relationship: We evaluated four doses of BsAb EVs (10, 20, 40, and 60 nmol). Flow cytometry demonstrated clear dose-dependent fibroblast depletion and T-cell activation across this range. Among the tested doses, 40 nmol produced strong activity, whereas 60 nmol did not provide an obvious additional benefit, suggesting that the efficacy plateau may already be approached at 40 nmol. Because no overt systemic toxicity was observed even at the highest tested dose, a formal maximum tolerated dose was not established in the current study. We have revised the manuscript to reflect this more precisely.

*Location in revised manuscript: Results section, Page 9, Lines 259-260, Figure S7A-N.*

**Comment 5:** *Most previous studies on epidural fibrosis (EF) have been conducted in rat models. The current study relies solely on a murine laminectomy model, which limits translational relevance. The authors are encouraged to validate the therapeutic efficacy and safety of BiTE EVs in a rat EF model, to confirm whether the observed anti-fibrotic*

*and immunomodulatory effects can be reproduced in a larger and more clinically representative system.*

**Response:** We thank the Reviewer for this valuable suggestion. We agree that validation in a rat EF model would strengthen translational relevance. In the current study, however, we prioritized proof-of-concept testing in an immunocompetent, species-matched murine system, since the BsAb construct is specific for murine FAP and murine CD3. As rat-reactive counterparts have not yet been developed, evaluation in a rat laminectomy model is not feasible with the present reagent set. We agree that development of species-matched reagents for larger-animal validation would be an important future direction.

**Comment 6:** *While the manuscript demonstrates depletion of FAP⁺ fibroblasts by BiTE EVs through flow cytometry and scRNA-seq, spatial confirmation by histological co-localization (e.g., FAP with CD3) is lacking. Immunofluorescence or spatial transcriptomic validation would strengthen the claim that BiTE EVs specifically eliminate FAP⁺ fibroblasts in situ without affecting neighboring stromal or neural cells.*

**Response:** We thank the Reviewer for this valuable suggestion. We have now added dual-color immunofluorescence staining for FAP and CD3 in epidural scar tissue. At postoperative day 14, BsAb EV-treated mice showed increased $CD3^+$ T-cell infiltration within the scar region, with clear spatial proximity and partial co-localization between CD3 and residual $FAP^+$ fibroblasts. These data provide spatial support for in situ T-cell recruitment and local engagement with target fibroblasts, complementing our flow-cytometric, scRNA-seq, and CD3-depletion results.

*Location in revised manuscript: Results section, Page 9, Lines 260-265, Figure S8A-C.*

**Comment 7:** *The proposed mechanism—where BiTE EV–mediated elimination of $FAP^+$ fibroblasts leads to subsequent immune modulation and fibrosis attenuation—is supported primarily by correlative findings from scRNA-seq and cytokine profiling. To strengthen the mechanistic interpretation, the authors are encouraged to perform functional validation experiments, such as T-cell depletion, FAP blocking, or rescue*

*assays, to directly test whether these downstream immunological changes indeed depend on FAP$^{+}$ fibroblast clearance. Such evidence would provide a more definitive link between fibroblast targeting and immune reprogramming.*

**Response:** We thank the Reviewer for this important suggestion. To strengthen the mechanistic interpretation beyond correlative scRNA-seq and cytokine profiling, we have now added two functional validation experiments.

**(1) FAP knockout model:** We compared fibrotic outcomes in WT and Fap knockout mice following laminectomy. Fap knockout mice exhibited markedly reduced epidural fibrosis at postoperative day 56, supporting an important pathogenic role for FAP in EF development. These data strengthen the rationale for targeting FAP$^{+}$ fibroblasts in this disease context.

*Location in revised manuscript: Results section, Page 6, Lines 151-155, Figure 1H-I.*

(2) T-cell depletion: We depleted T cells in vivo using an anti-CD3 monoclonal antibody prior to BsAb EVs treatment. Under these conditions, the fibroblast-reducing effect of BsAb EVs was abolished, indicating that the therapeutic activity of BsAb EVs is T-cell-dependent in vivo. Together with the spatial proximity of CD3$^{+}$ cells and FAP$^{+}$ fibroblasts in wound sections, these data provide functional support for local immune redirection against FAP$^{+}$ fibroblasts.

*Location in revised manuscript: Results section, Page 9, Lines 262–265; Figure S8D–I.*

We believe these added functional data substantially strengthen the mechanistic support for the proposed model.

**Comment 8:** *How does the advantage of BiTE EVs compare to traditional physical barrier therapies (such as biomaterials) and FAP inhibitors (FAPi as discussed in the manuscript)? Are the benefits of BiTE EVs quantifiable, such as the duration of EF relief, the likelihood of reducing revision surgery risks, and cost-effectiveness? It is recommended to include head-to-head comparison experimental data and related discussion to address these points.*

**Response:** We thank the Reviewer for this important question regarding clinical context

and translational positioning.

(1) Comparison with physical barrier strategies: We agree that comparison with established physical barrier approaches is clinically relevant. However, the present study was designed as a biologically targeted proof-of-concept study rather than a device-versus-biologic benchmarking study. Physical barriers act primarily by mechanically separating tissues during the early postoperative period, whereas BsAb EVs are intended to actively remodel the local cellular microenvironment through immune-mediated depletion of pathogenic $FAP^+$ fibroblasts. Because these strategies differ substantially in mechanism, delivery paradigm, and intended mode of action, we did not perform a direct head-to-head comparison in the current study.

(2) Comparison with FAP inhibition: We included Ac-Gly-BoroPro as a pharmacologic comparator to assess whether systemic inhibition of FAP could suppress postoperative fibrosis. Under the tested conditions, FAPi reduced fibrosis-related readouts but was also associated with transient body-weight loss and compromised survival, whereas BsAb EVs showed antifibrotic efficacy together with a more favorable safety profile, lesion retention, and T-cell-dependent activity in vivo. We have therefore interpreted the BsAb EVs approach as offering improved therapeutic selectivity in this model relative to the tested FAPi regimen.

*Location in revised manuscript: Results section, Page 7, Lines 204–206, Figure S3J–K.*

(3) Duration of benefit, revision-risk reduction, and cost-effectiveness: We agree that these are important translational questions. The current study was primarily designed to establish proof-of-concept efficacy, mechanistic support, and safety in an immunocompetent preclinical model. Within this framework, BsAb EVs showed sustained antifibrotic effects through the observation period together with a favorable safety profile. Whether this approach can durably reduce the clinical burden of epidural fibrosis, lower revision-surgery risk, or offer advantages in cost-effectiveness will require dedicated longitudinal and clinical evaluation in future studies.

**Comment 9:** *Why were NIH/3T3 cells chosen as the source for EVs? Compared to EVs*

*derived from clinically commonly used mesenchymal stem cells (MSCs), do NIH/3T3-derived EVs offer superior immunogenicity, targeting ability, and safety? A comparison with EVs from different sources should be included to validate these aspects.*

**Response:** We thank the Reviewer for this important comment. NIH/3T3 cells were selected because they are a well-established murine fibroblast cell line that enables efficient genetic engineering, robust expansion, and reproducible EVs production. Their murine origin also makes them suitable for proof-of-concept testing in our immunocompetent mouse model. We agree that producer-cell origin can influence EVs composition and may affect biodistribution, immunogenicity, and translational feasibility. In the revised Discussion, we now clarify that future studies should evaluate whether EVs from more translationally relevant sources, such as mesenchymal stromal cells (MSCs), can preserve therapeutic activity while offering additional advantages for clinical development.

*Location in revised manuscript: Discussion section, Page 13, Lines 417–423.*

**Minor Issues 1–13:**

We have carefully addressed all minor issues as follows:

**1.** *The clinical sample size for PET/CT is small, with only 6 cases currently providing preliminary validation of the target. It is recommended to include an additional 10-15 patients to analyze the correlation between FAP expression and the severity of EF.*

**Response:** The clinical $^{68}$Ga-FAPI PET/CT cohort has been expanded to n = 10 patients, including 5 open-surgery and 5 endoscopic-surgery cases.

**2.** *The manuscript only assesses short-term liver and kidney functon (56 days) and systemic inflammatory responses, lacking long-term safety data. Will BiTE EVs induce T cell overactivation or off-target effects (such as attacking cells with low FAP expression in normal tissues)? Could long-term use lead to immune tolerance or autoimmune reactions? It is recommended to include data extending beyond 3 months to address these concerns.*

**Response:** We expanded the in vivo safety assessment to include liver/kidney function and hematology at postoperative days 7, 14, 28, and 56, and no significant abnormalities

were detected. In addition, multiplex serum cytokine analysis at postoperative days 1, 4, 7, and 14 showed only mild and transient changes, without evidence of marked systemic immune overactivation. We agree that evaluation beyond 3 months, as well as longer-term assessment of immune tolerance or autoimmune sequelae, would be valuable; however, the current study was designed to assess efficacy and safety within a relatively short perioperative treatment and observation window. Within this framework, BsAb EVs showed a favorable safety profile.

**3.** *Figure 1C, correct "per mousel" to "per mouse".*

**Response:** "per mousel" has been corrected to "per mouse."

**4.** *Figure S1 mixes clinical and murine data but does not label source by panel. Please specify the sample origin for each subpanel in the legend.*

**Response:** The legend now specifies the sample origin (clinical vs. murine) for each panel.

**5.** *The authors exclusively used female BALB/c mice for the in vivo laminectomy model, but the rationale for this choice is not provided in the manuscript. Given that both strain- and sex-dependent differences can markedly influence immune responses and fibrotic remodeling, it would be important to briefly justify this selection.*

**Response:** Additional experiments were performed using both male and female BALB/c mice, and no sex-dependent differences in therapeutic response were observed. In addition, both male and female FAP-KO mice on a BALB/c background were included in the study, with no apparent sex-based differences observed. The Methods section has been revised accordingly.

**6.** *There is an apparent mismatch between the Results text and the labeling of Figure 2C and Figure S9D-E. Please ensure full consistency among the main text, figure panels, and the legend (including statistics/conditions).*

**Response:** Inconsistencies between the main text and Figures 2C and S9D-E have been corrected.

**7.** *At least a brief explanation of the target protein should be provided in the figure legend of Figure 3F, clarifying the rationale for its detection.*

**Response:** The Figure 3F legend has been revised to briefly explain why these proteins

were examined, namely to characterize EV-associated markers, assess incorporation of the engineered BsAb construct, and exclude obvious cellular contamination.

**8.** *The abbreviation "MPs" used in Figure 4 is insufficiently defined. The authors should clarify in the legend or Methods that it refers to mononuclear phagocytes to improve clarity and readability.*

**Response:** "MPs" is now defined as "mononuclear phagocytes" in the legend and Methods section.

**9.** *In Figure S5E, the color scales of the heatmaps for WT EVs and BiTE EVs are not unified, which reduces the direct visual comparability between groups. It is recommended to use a consistent color scale or normalized value range across both conditions to ensure accurate visual interpretation of intercellular communication intensity.*

**Response:** The color scales in Figure S5E (now Figure S9E) have been unified for accurate visual comparison. Location in revised manuscript: Figure S9E

**10.** *In the sentence "...genes previously linked to fibroblast activation (Figure 5I–J, Figure S6C–E)", the figure citation appears to be incorrect. The referenced data correspond to Figure S8C–E, not S6C–E. Please correct the figure numbering accordingly.*

**Response:** The incorrect figure citation has been corrected from Figure S6C–E to Figure S12C–E.

**11.** *The figure legend should specify the number of animals used in each group.*

**Response:** Sample sizes (n) are now clearly indicated in all relevant figure legends.

**12.** *There is an inconsistency regarding the source of EVs. The Methods section states that BiTE EVs were generated from engineered NIH/3T3 fibroblasts, whereas the Discussion refers to HEK293-derived EVs. This discrepancy should be clarified to ensure consistency and transparency in the description of EV origin.*

**Response:** The discrepancy regarding EVs source has been corrected. All EVs described in this study were derived from engineered NIH/3T3 cells, and the reference to HEK293-derived EVs in the Discussion was a typographical error.

**13.** *The legend for Figure 3B is not precise. The panel illustrates the design and*

*proposed working principle of FAP×CD3 BiTE EVs, rather than the "mechanism of fibrosis alleviation."*

**Response:** The legend has been revised to accurately describe the panel as the "design and proposed working principle of FAP×CD3 BsAb EV". Location in revised manuscript: Figure legend section, Page 31, Lines 1036–1037

**Reviewer #2**

**General Comment:** *The study presents an immunotherapeutic approach using BiTE-engineered extracellular vesicles to target FAP$^+$ fibroblasts, however, several critical issues preclude its acceptance in its current form. My major concerns are as follows...*

**Response:** We thank the Reviewer for the careful evaluation of our work and for these important comments. We have substantially revised the manuscript and added new experimental data to address the major concerns raised, including additional functional validation, expanded safety analyses, and more comprehensive characterization of the BsAb EVs platform. Our point-by-point responses are provided below.

**Comment 1:** *The authors claim that BiTE EVs did not induce systemic toxicity based on serum ALT, AST, BUN, and CR levels at a single early time point (postoperative day 5). However, this limited analysis is inadequate to rule out potential immunotoxicity, especially given the systemic administration of a T cell-redirecting therapy. No data are provided on systemic T cell activation, cytokine release syndrome (CRS), or immune cell infiltration in non-target organs (e.g., lung, liver, spleen). The absence of long-term toxicity data is concerning, particularly given the potential for delayed autoimmune or off-target effects. Uncontrolled T cell activation in non-target tissues could lead to autoimmune pathology, which was not evaluated.*

**Response:** We thank the Reviewer for this important comment. We agree that careful assessment of systemic toxicity is particularly important for any T-cell-redirecting therapy. To address this concern, we have substantially expanded the safety evaluation in the revised manuscript.

(1) Extended organ function and hematology: We extended the analysis of liver and

kidney function markers (ALT, AST, BUN, and creatinine) and complete blood counts to postoperative days 7, 14, 28, and 56. Across all tested BsAb EV dose groups, no significant abnormalities were detected compared with WT EV-treated controls. In addition, histological examination of major solid organs at postoperative day 56 did not reveal overt tissue injury.

*Location in revised manuscript: Results section, Page 12, Lines 355–374, Figure S14 and Figure S15.*

(2) Systemic T-cell activation: To assess potential systemic T-cell activation, we analyzed splenic T-cell populations across the tested dose groups. Flow-cytometric analysis did not reveal notable differences in the proportion or activation status of splenic T cells between WT EV-treated and BsAb EV-treated mice. These findings do not support marked systemic T-cell activation under the tested conditions.

*Location in revised manuscript: Results section, Page 9, Lines 259–260; Figure S7A–N.*

(3) Cytokine assessment: We also performed multiplex cytokine analysis on serum collected at postoperative days 1, 4, 7, and 14. This showed only mild and transient cytokine changes, without the broad multi-fold elevation pattern typically associated with cytokine release syndrome-like systemic immune overactivation.

*Location in revised manuscript: Results section, Page 12, Lines 368–374; Figure S14N–W.*

(4) Non-target organs: Finally, H&E staining of heart, lung, liver, spleen, and kidney harvested at postoperative day 56 showed no evidence of overt structural injury, tissue necrosis, or obvious pathological inflammatory changes in BsAb EV-treated animals. Together with the serum chemistry, hematology, cytokine, and splenic flow-cytometry data, these findings support a favorable systemic safety profile within the studied time frame.

*Location in revised manuscript: Results section, Page 12, Lines 365–367; Figure S15.*

We agree that even longer-term evaluation, including potential delayed autoimmune or off-target immune consequences, would be valuable. However, within the current perioperative treatment and observation window, we did not detect evidence of marked

systemic immunotoxicity.

**Comment 2:** *The study relies heavily on observational and correlative data but falls short in providing direct mechanistic evidence. While FAP⁺ fibroblasts are implicated in fibrosis, no genetic (e.g., FAP knockout) or lineage-tracing models were used to confirm their necessity in epidural fibrosis. In addition, although BiTE EVs are proposed to recruit T cells, no in vivo T cell depletion or adoptive transfer experiments were performed to confirm that the antifibrotic effect is T cell-dependent. Moreover, the scRNA-seq analysis is descriptive and lacks functional validation.*

**Response:** We thank the Reviewer for this important comment. We agree that functional validation is essential to strengthen the mechanistic interpretation beyond observational and correlative analyses. To address this point, we have now added two key functional validation experiments.

(1) Genetic support for the role of FAP in epidural fibrosis: We compared epidural fibrosis severity following laminectomy in wild-type (WT) and FAP knockout mice. At postoperative day 56, FAP knockout mice exhibited markedly reduced epidural fibrosis compared with WT controls. These data support an important pathogenic role for FAP in the development of post-laminectomy epidural fibrosis and strengthen the rationale for targeting $FAP^+$ fibroblasts in this setting.

*Location in revised manuscript: Results section, Page 6, Lines 151–155; Figure 1H–I.*

(2) Functional validation of T-cell dependence: We depleted T cells in vivo using an anti-CD3 monoclonal antibody prior to BsAb EV treatment. Under these conditions, the fibroblast-reducing effect of BsAb EVs was abolished, indicating that the therapeutic activity of BsAb EVs is T-cell-dependent in vivo. Together with the spatial proximity of $CD3^+$ cells and $FAP^+$ fibroblasts in wound sections, these findings provide functional support for local immune redirection against $FAP^+$ fibroblasts.

*Location in revised manuscript: Results section, Page 9, Lines 262–265; Figure S8D–I.*

(3) Role of the scRNA-seq analysis: We agree that the scRNA-seq analysis is descriptive in nature. In the revised manuscript, we have therefore adjusted the

language to avoid overinterpreting these data as causal evidence.

**Comment 3:** *Regarding characterization of BiTE EVs, functional stability was only assessed up to 48 hours at room temperature. Stability under physiological conditions (e.g., in serum) and in vivo half-life were not evaluated. The composition, cargo, and potential immunogenicity of BiTE EVs were not thoroughly characterized*

**Response:** We thank the Reviewer for this important comment. To address these concerns, we expanded the characterization of BsAb EVs in several ways.

(1) Stability under physiological conditions: We evaluated BsAb EV stability after incubation in both PBS and mouse serum at 37°C. Although particle abundance and GFP-associated signal gradually declined over time, BsAb EVs retained detectable T-cell-mediated cytotoxic activity under both conditions for up to 48 h. These findings support partial functional stability under physiologic incubation conditions rather than complete structural stability.

*Location in revised manuscript: Results section, Page 8, Lines 247–249; Figure S5E–G.*

(2) In vivo persistence: We agree that direct measurement of in vivo circulating half-life would be informative, but this was not directly assessed in the current study. Instead, our in vivo data show FAP-dependent lesion retention together with dose-dependent fibroblast depletion and T-cell activation, supporting that BsAb EVs persist long enough to achieve biologically meaningful local activity.

*Location in revised manuscript: Results section, Page 9, Lines 253–254, Figure S6A–F; Page 9, Lines 259–260, Figure S7A–N.*

(3) EV composition and cargo: We supplemented the characterization of EV preparations with additional protein, lipid, and RNA analyses. Western blot showed detectable TSG101 and CD63, while Calnexin was undetectable, supporting EV-enriched preparations with minimal obvious cellular contamination. Lipidomic profiling revealed increased relative abundance of fatty acids and phospholipids, together with reduced glycerolipids, in BsAb EVs compared with WT EVs. Total RNA content did not differ significantly between WT EVs and BsAb EVs.

*Location in revised manuscript: Results section, Page 8, Lines 220–230, Figure 3G-I, Figure S4C-E.*

(4) Immunogenicity-related assessment: In our immunocompetent BALB/c model, repeated systemic administration of BsAb EVs did not result in overt systemic toxicity, marked cytokine-release-like responses, or histopathological evidence of organ injury within the studied time frame. These findings do not suggest a deleterious host response under the tested conditions, although longer-term immunogenicity will require further study.

*Location in revised manuscript: Results section, Page 12, Lines 355–374; Figure S14 and Figure S15.*

**Reviewer #3**

**General Comment:** *This study explores the use of bispecific T cell engager-decorated extracellular vesicles (BiTE EVs) targeting FAP and CD3 to mitigate epidural fibrosis (EF) in a murine laminectomy model. While the concept is interesting, the manuscript suffers from several critical weaknesses that undermine the validity and impact of the findings. The study lacks focus, with insufficient evidence to support the proposed mechanism of action of BiTE EVs. The connection between clinical observations and experimental data is tenuous, and key methodological details are missing. The single-cell RNA sequencing data, while extensive, do not convincingly demonstrate the proposed cellular interactions or therapeutic mechanisms.*

**Response:** We are grateful to the Reviewer for this critical and constructive feedback. We have carefully considered each point and have substantially revised the manuscript, adding new experimental data to strengthen the mechanistic narrative, clarify the clinical context, and provide the missing methodological details.

**Comment 1:** *The manuscript posits a link between the technical difficulties of revision spine surgery and epidural fibrosis, but the evidence presented is circumstantial. While clinical data confirm that the revision cohort had more complicated perioperative metrics (e.g., longer operative time, greater blood loss), these findings are only*

*correlative and do not demonstrate that epidural fibrosis was the underlying cause.*

**Response:** We agree with the Reviewer that perioperative metrics such as operative time and blood loss should be interpreted cautiously and do not by themselves define the precise determinants of surgical complexity. We have revised the manuscript to acknowledge this point more explicitly. In the clinical setting, direct quantification of epidural scar severity remains challenging because objective intraoperative assessment is not standardized and often remains surgeon dependent. We therefore present these findings as supportive translational context that motivates the study, while relying on the murine model to investigate the underlying cellular mechanisms in a more controlled setting.

*Location in revised manuscript: Results section, Page 5, Lines 129–138; Discussion section, Page 13, Lines 409–415.*

**Comment 2:** *The MRI and PET/CT imaging data are supportive but do not directly prove mechanistic claims. The use of $^{68}$Ga-FAPI PET/CT imaging in Figure 1A to indicate fibroblast activation is not sufficiently justified. Given the cellular heterogeneity of surgical tissue, the observed radiotracer uptake cannot be definitively attributed to fibroblasts alone. Consequently, the assertion that the signal reflects activated fibroblasts requires further validation.*

**Response:** We thank the Reviewer for this important comment. We agree that the MRI and $^{68}$Ga-FAPI PET/CT data are supportive and should not be interpreted as direct mechanistic proof. We also agree that radiotracer uptake at the surgical site cannot, by itself, be definitively attributed to fibroblasts alone in a heterogeneous postoperative tissue environment. We have therefore revised the manuscript to frame the PET/CT findings more cautiously, as evidence of FAP-associated postoperative stromal remodeling rather than definitive proof of activated fibroblasts in the clinical cohort.

Our interpretation is supported by the subsequent experimental data in the murine model, where postoperative FAP upregulation and enrichment of $FAP^+$ fibroblasts were validated by IHC, Western blot, qPCR, and flow cytometry. We have also clarified in the revised manuscript that the clinical imaging findings are intended as supportive

translational context, while the mechanistic conclusions are derived primarily from the controlled in vivo and ex vivo analyses.

*Location in revised manuscript: Results section, Page 5, Lines 129–138; Discussion section, Page 13, Lines 409–415.*

**Comment 3:** *The core claim—that BiTE EVs simultaneously engage FAP⁺ fibroblasts and T cells to induce targeted cytotoxicity—is not adequately supported. In vitro co-culture experiments are needed to demonstrate that BiTE EVs facilitate T cell–fibroblast conjugation and that cytotoxicity is specifically dependent on both FAP and CD3 engagement (e.g., via blocking antibodies).*

**Response:** We thank the Reviewer for this important comment. To strengthen support for the proposed mechanism, we added additional in vitro and in vivo functional data.

(1) Ternary in vitro co-culture system: We co-cultured T cells with FAP-positive target cells in the presence of BsAb EVs. Under these ternary conditions, BsAb EVs induced robust T-cell activation, as evidenced by increased CD107a expression and elevated intracellular Granzyme B, TNF-α, and IFN-γ, together with dose-dependent target-cell killing. By contrast, BsAb EVs alone induced only modest T-cell activation, and substantial cytotoxicity was observed only in the presence of both T cells and FAP-positive target cells. These findings support target-dependent immune redirection by BsAb EVs in vitro.

*Location in revised manuscript: Results section, Page 8, Lines 240–247; Figure 4E–I and Figure S5A–D.*

(2) Specificity controls: Incubation of FAP-positive target cells with BsAb EVs in the absence of T cells resulted in negligible direct cytotoxicity, indicating that BsAb EVs do not directly kill fibroblasts on their own. In addition, BsAb EV-treated T cells did not mediate significant killing of FAP-negative target cells, supporting target dependence under the tested conditions.

*Location in revised manuscript: Results section, Page 8, Lines 240–244; Figure 4I and Figure S5A–D.*

(3) Spatial and in vivo functional support: In wound sections from BsAb EV-treated

mice, we observed increased $CD3^+$ T-cell infiltration together with spatial proximity and partial co-localization of CD3 and FAP signals, consistent with local engagement of T cells and target fibroblasts in situ. In addition, depletion of $CD3^+$ T cells in vivo abolished the fibroblast-reducing effect of BsAb EVs, supporting that the therapeutic activity is T-cell-dependent in vivo.

*Location in revised manuscript: Results section, Page 9, Lines 259–265; Figure S7A–N, Figure S8A–I.*

Together, these findings support the proposed BsAb-mediated redirection of T-cell cytotoxicity toward FAP-positive fibroblasts and indicate T-cell-dependent therapeutic activity in vivo.

**Comment 4:** *The possibility that BiTE EVs may directly affect other cell types (e.g., neutrophils) or act through non-specific immune modulation is not ruled out.*

Response: Because neutrophils showed a relatively marked change in the scRNA-seq analysis, we performed additional experiments to assess whether BsAb EVs directly affect this population. In vitro exposure of primary neutrophils to BsAb EVs did not induce significant activation or chemotactic migration. These data do not support a direct effect of BsAb EVs on neutrophils under the tested conditions. The neutrophil changes observed in vivo are therefore more likely to reflect secondary remodeling of the local wound environment after treatment.

*Location in revised manuscript: Results section, Page 11, Lines 329-336, Figure S13A-F*

**Comment 5:** *Key details are missing, such as the method for labeling fibroblasts in flow cytometry (Figure 3N). Figure legends and descriptions are overly simplistic and fail to clarify experimental conditions, statistical methods, or the meaning of key annotations (e.g., red arrows in Figure 1A). Inconsistencies between figure panels and their descriptions (e.g., Figure 2C, 2D) further undermine confidence in the data.*

**Response:** We thank the Reviewer for pointing out these issues in figure clarity and methodological description. We have carefully revised the relevant sections to improve

transparency and consistency throughout the manuscript.

(1) Flow-cytometry methodology and gating: We have now clarified the fibroblast labeling strategy in the Methods section and added the corresponding gating strategy and control information for the relevant flow-cytometry experiments.

*Location in revised manuscript: Methods section; Figure S16.*

(2) Figure legends: We have substantially revised the figure legends to provide clearer descriptions of experimental conditions, sample origin where relevant, statistical annotations, abbreviations, and panel-specific annotations (including the red arrows in Figure 1A).

*Location in revised manuscript: Figure legend section.*

(3) Figure-text consistency: The inconsistencies between the figure panels and the corresponding text/legend descriptions, including those in Figure 2C and 2D, have been corrected in the revised manuscript.

**Comment 6:** *The scRNA-seq data reveal changes in cell proportions (e.g., increased neutrophils) but do not establish a direct link between BiTE EV treatment and T cell–mediated fibroblast depletion. The data suggest broader immunomodulatory effects (e.g., on MPs and neutrophils), raising the question of whether the observed benefits are truly due to T cell–mediated cytotoxicity or secondary to other immune changes. The trajectory and communication analyses are descriptive and lack functional validation.*

**Response:** We agree that scRNA-seq analyses are primarily descriptive and require orthogonal functional validation to support mechanistic interpretation. To address this concern, we added in vivo T-cell depletion experiments and supplementary neutrophil assays.

(1) T-cell dependence: In vivo depletion of $CD3^+$ T cells abolished the fibroblast-reducing effect of BsAb EV treatment, supporting that the therapeutic effect is T-cell-dependent in vivo rather than being explained solely by broader nonspecific immune modulation.

*Location in revised manuscript: Results section, Page 9, Lines 259–265; Figure S8D–*

*I.*

(2) Additional support regarding broader immune changes: Because neutrophils showed a relatively marked change in the scRNA-seq dataset, we performed additional in vitro assays and did not detect significant direct activation or chemotactic recruitment by BsAb EVs. These findings support the interpretation that the neutrophil changes are more likely secondary to local wound remodeling after treatment rather than the primary mechanism of action.

*Location in revised manuscript: Results section, Page 11, Lines 329–336; Figure S13A–F.*

**Comment 7:** *The expression of FAP in normal dura mater is not provided as a baseline for injury-induced changes (Figure 1C–E). The influence of BiTE EVs on T cells or fibroblasts in the absence of the other cell type is not tested, leaving open the possibility of off-target or indirect effects.*

**Response:** We thank the Reviewer for this important comment.

(1) Baseline FAP expression: We have now included additional baseline tissue analysis to provide context for injury-induced FAP upregulation. In normal, uninjured dura mater, FAP expression was minimal to undetectable, in contrast to the marked increase observed at the epidural scar after laminectomy.

*Location in revised manuscript: Results section, Page 6, Lines 150–151; Figure S2C–D.*

(2) Direct effects on isolated cell types: As described in response to Comment 3, BsAb EVs induced only mild T-cell activation in the absence of fibroblasts and caused negligible direct cytotoxicity toward fibroblasts in the absence of T cells. These results argue against substantial direct off-target effects on either cell type alone.

**Comment 8:** *A critical gap in this study is the lack of direct mechanistic evidence demonstrating how BiTE EVs mediate their effects. It is essential to perform reductionist in vitro experiments to deconvolute this interaction. Specifically, the authors should investigate whether BiTE EVs directly activate or modulate T cells in*

*the absence of fibroblasts, and conversely, whether they have any direct, T-cell-independent effects on fibroblast biology. Furthermore, definitive evidence is required to show that BiTE EVs can simultaneously bind both FAP-positive fibroblasts and T cells to form a ternary complex, thereby physically bridging the two cell types to enable targeted cytotoxicity.*

**Response:** We thank the Reviewer for this important comment. As described in our response to Comment 3, we have now added reductionist in vitro experiments to better dissect the mechanism of action of BsAb EV.

(1) T-cell modulation in isolation: T cells cultured with BsAb EV alone showed only mild activation and did not mediate substantial cytotoxicity.

(2) Direct effect on fibroblasts: FAP-positive fibroblasts cultured with BsAb EVs in the absence of T cells showed negligible direct cytotoxicity.

(3) Ternary co-culture context: Robust T-cell activation and specific target-cell killing were observed only when T cells, FAP-positive target cells, and BsAb EVs were present together in co-culture. In addition, wound-section immunofluorescence showed increased $CD3^+$ T-cell infiltration together with spatial proximity and partial co-localization of CD3 and FAP signals in BsAb EV-treated mice. Together, these data support the proposed model that BsAb EVs redirect T-cell cytotoxicity toward FAP-positive fibroblasts and are consistent with local engagement of the two cell types in situ.

**Comment 9:** *A more quantitative and direct characterization of the BiTE EVs is critical. First, the average number of anti-FAP/anti-CD3 scFv molecules displayed per EV particle should be quantified, as this directly influences the functional avidity of the EVs. Second, definitive in vitro evidence demonstrating the specific binding of these BiTE EVs to FAP-expressing fibroblasts (compared to FAP-negative controls) is required to substantiate the platform's foundational targeting mechanism.*

**Response:** We thank the Reviewer for this important comment. We have now added both quantitative and functional data to further characterize the BsAb EV platform.

(1) Estimation of BsAb loading per EV particle: Using GFP fluorescence calibrated

against a recombinant GFP standard curve and normalized to NTA-derived particle counts, we estimated an average construct loading of approximately $1.51 \times 10^4$ GFP-tagged BsAb molecules per EV particle across three independent batches.

*Location in revised manuscript: Results section, Page 8, Lines 233–235.*

(2) Targeting specificity: We further evaluated targeting specificity in vivo using the Fap knockout model. Following laminectomy and systemic administration, BsAb EVs showed robust lesion accumulation in WT mice, whereas this enrichment was markedly diminished in Fap knockout mice, supporting FAP-dependent retention in vivo. In addition, the target-dependent T-cell activation and cytotoxicity observed in the ternary co-culture system are consistent with selective engagement of FAP-positive target cells under the tested conditions.

*Location in revised manuscript: Results section, Page 9, Lines 253-254, Figure S6E-F*

**Response to Reviewers' Comments**

**Title: Bispecific Antibody Engineered Extracellular Vesicles Redirect T Cells to Prevent Postoperative Epidural Fibrosis**

We sincerely thank reviewers and the editorial team for the careful re-evaluation of our revised manuscript and for the additional constructive suggestions. These comments have helped us further improve the precision, balance, and presentation of our work. We have carefully addressed each point in the revised manuscript. To facilitate evaluation of the revision, we directly quote the key revised text passages in the responses below. For clarity, the comments are shown in italic font, followed by our responses in plain text.

**Reviewer #1:**

**General Comment:** *I have carefully evaluated the revised manuscript and the authors' response to the previous round of review. Overall, the revised manuscript is substantially improved. The authors have added important experimental data, including FAP-deficient mice, dose-response analysis, in vitro and in vivo T-cell-dependent functional validation, and a more comprehensive safety assessment. These additions significantly strengthen the technical and mechanistic foundation of the study. Nevertheless, several points still require clarification or more cautious wording before publication.*

**Response:** We thank the Reviewer for the positive assessment of our revisions and for the additional suggestions, which we have fully addressed as detailed below.

**Comment 1:** *The current data show that BsAb EVs attenuate postoperative fibrotic remodeling when administered during the early postoperative period. However, the study does not demonstrate reversal of established, mature epidural fibrosis. I therefore recommend replacing "reverse" with "attenuate", "mitigate", or "prevent" in the title and throughout the manuscript where appropriate.*

**Response:** We agree with the Reviewer's assessment. We have changed the title to "Bispecific antibody engineered extracellular vesicles redirect T cells to prevent

postoperative epidural fibrosis" and revised the wording throughout the manuscript accordingly.

**Revised text:**

(1) "In a preclinical model, BsAb EVs selectively eliminate pathogenic fibroblasts, reduce fibrotic collagen accumulation, and prevent the development of postoperative epidural fibrosis without detectable systemic toxicity under the tested conditions." (Location in revised manuscript: Abstract section, Page 3, Lines 61–64)

(2) "Collectively, these findings establish a proof of concept that FAP targeted BsAb engineered EVs can redirect endogenous T cells to selectively eliminate pathogenic fibroblasts and prevent the development of postoperative epidural fibrosis." (Location in revised manuscript: Introduction section, Page 5, Lines 107–110)

We have also replaced "reverse" with "attenuate" or "prevent" in other relevant locations in the Results and Discussion sections where appropriate.

**Comment 2:** T*he clinical revision-surgery cohort and FAPI PET/CT imaging provide useful translational context, but they remain correlative. The imaging cohort includes only a single postoperative time point and lacks direct histological correlation. Moreover, the PET/CT signal cannot be definitively attributed to fibroblasts alone in heterogeneous postoperative tissue. The manuscript should consistently describe the clinical data as supportive evidence of FAP-associated stromal remodeling, rather than direct proof of activated fibroblast accumulation or causal involvement in revision-surgery difficulty.*

**Response:** We agree with the Reviewer's assessment. We have revised the clinical FAPI PET/CT description to present the findings as supportive, correlative evidence of FAP-associated stromal remodeling, rather than direct proof of activated fibroblast accumulation or causal involvement in revision-surgery difficulty.

**Revised text:**

"Because postoperative scarring and epidural fibrosis are commonly considered contributors to the technical difficulty of revision lumbar surgery (1, 4), we next sought supportive clinical evidence of FAP-associated tissue remodeling at the surgical site.

68Ga-FAPI PET/CT imaging was performed one week postoperatively in ten patients who underwent lumbar decompression, including five who underwent open surgery and five who underwent endoscopic surgery. All patients demonstrated intense, focal radiotracer uptake along the surgical trajectories, providing supportive evidence of active FAP-associated stromal remodeling at the operative site (Figure 1A)." (Location in revised manuscript: Results section, Page 3, Lines 128–135)

**Comment 3:** *The FAP-KO experiment is an important addition and supports a functional role for FAP in postoperative fibrosis. However, because this is a global FAP-deficient model, it does not exclude contributions from non-fibroblast FAP-expressing stromal compartments or indirect developmental/repair effects. The authors should avoid wording that implies definitive fibroblast-specific genetic causality.*

**Response:** We appreciate the Reviewer's careful interpretation. We have revised the corresponding sentence to avoid implying fibroblast-specific causality and to acknowledge the global nature of the *Fap* deletion.

**Revised text:**

"These findings indicate that genetic loss of Fap attenuates postoperative epidural fibrosis, supporting a functional contribution of FAP to postoperative fibrotic remodeling in this model." (Location in revised manuscript: Results section, Page 6, Lines 155–157)

**Comment 4:** *The in vitro macrophage–fibroblast experiments support a plausible regulatory mechanism. However, direct in vivo validation of macrophage-derived SPP1/TGF-β1 signaling is still limited. I recommend using language such as "contributes to" or "is associated with" rather than stating that this axis definitively drives EF progression.*

**Response:** We agree with the Reviewer's assessment. We have revised the section heading and concluding sentence to avoid implying definitive in vivo causality and to use more cautious language.

**Revised text:**

(1) "$SPP1^{+}$ macrophage-associated signaling contributes to FAP upregulation after spine surgery" (Location in revised manuscript: Results section, Page 6, Lines 174–175)
(2) "Together, these findings suggest that M2 macrophages contribute to fibroblast activation and FAP upregulation through SPP1–TGF-β1 signaling." (Location in revised manuscript: Results section, Page 7, Lines 188–189)

**Comment 5:** *The expanded safety assessment is reassuring within the tested perioperative mouse model. However, long-term immunogenicity, repeated dosing, autoimmune sequelae, and toxicity against low-level FAP-expressing normal tissues remain unresolved. The phrase "without systemic toxicity" should be revised to "without detectable systemic toxicity under the tested conditions" or similar wording.*
**Response:** We agree with the Reviewer's assessment. We have revised the safety-related language in the Abstract and Results to avoid overgeneralizing the safety conclusions and to specify that no detectable systemic toxicity was observed under the tested conditions.
**Revised text:**
(1) "In a preclinical model, BsAb EVs selectively eliminate pathogenic fibroblasts, reduce fibrotic collagen accumulation, and prevent the development of postoperative epidural fibrosis without detectable systemic toxicity under the tested conditions." (*Location in revised manuscript: Abstract section, Page 3, Lines 61–64*)
(2) "Collectively, these results indicate that BsAb EV treatment is well tolerated in the tested perioperative mouse model without detectable acute hepatorenal, hematological, or major histological toxicity." (*Location in revised manuscript: Results section, Page 12, Lines 382–384*)

**Comment 6:** *The lack of rat or larger-animal validation, the use of systemic rather than local delivery, and the use of NIH/3T3-derived EVs remain important limitations. These issues do not necessarily preclude publication as a proof-of-concept study, but they should be clearly acknowledged.*

**Response:** We have revised the Limitations paragraph in the Discussion to explicitly acknowledge each of these points, as shown below.

**Revised text:**

(1) "Second, our in vivo efficacy studies were performed in a mouse laminectomy model, and validation in rat or larger-animal models will be important to better assess surgical anatomy, dosing, biodistribution, and translational feasibility." (Location in revised manuscript: Discussion section, Page 14, Lines 426–428)

(2) "Third, although local delivery may be attractive for surgically accessible postoperative lesions, BsAb EVs were administered systemically in this proof-of-concept study, and the optimal route of administration remains to be determined." (Location in revised manuscript: Discussion section, Page 14, Lines 428–431)

(3) "Finally, an additional limitation relates to the EV platform itself. The properties of EV-based platforms are strongly influenced by producer-cell origin (34, 35). The NIH/3T3 fibroblasts used here provide a robust engineering platform, but this cell line is not a clinically preferred EV source. Future studies should evaluate whether producer cells with greater translational relevance, such as mesenchymal stromal cells (MSCs), can preserve therapeutic activity while potentially improving biodistribution, immunogenicity, and clinical feasibility." (Location in revised manuscript: Discussion section, Page 14, Lines 433–439)

**Comment 7:** *The page numbers in the current PDF version are not clearly visible, which makes it difficult to verify the page and line references provided in the response letter. Please ensure that page numbers are clearly displayed in the revised manuscript PDF.*

**Response:** We apologize for the oversight. We have ensured that page numbers are clearly displayed on every page of the revised manuscript PDF to facilitate verification of the page and line references in the response letter.

**Comment 8:** *The response to Comment 8 should be further incorporated into the Discussion section. Although the authors addressed the comparison between BiTE EVs,*

*conventional physical barrier strategies, and pharmacological FAP inhibition in the response letter, the corresponding discussion does not appear to be sufficiently incorporated into the revised manuscript. Please add a dedicated paragraph in the Discussion section to clarify the translational positioning of BiTE EVs relative to existing anti-epidural fibrosis approaches, including physical barrier materials and FAP inhibitors.*

**Response:** We thank the reviewer for this helpful suggestion. We have revised the Discussion to more clearly clarify the translational positioning of BsAb-engineered EVs relative to existing anti-epidural fibrosis approaches. Specifically, we now emphasize that BsAb EVs represent a cell-directed immunotherapeutic strategy that extends beyond conventional perioperative approaches based on physical separation or pharmacological modulation of fibrotic pathways. We also highlight the potential relevance of this platform for postoperative fibrosis, where lesions are localized and surgically accessible.

**Revised text:**

"Building on this conceptual framework, our BsAb-engineered EVs provide a cell-directed immunotherapeutic strategy that extends beyond conventional perioperative approaches based on physical separation or pharmacological modulation of fibrotic pathways. From a translational perspective, the EV platform may be particularly attractive for postoperative fibrosis, where lesions are localized and surgically accessible. BsAb EVs could potentially be administered directly to the wound site to enhance local efficacy while limiting systemic exposure." (*Location in revised manuscript: Discussion section, Page 13, Lines 401–407*)

**Comment 9:** *The response letter would benefit from directly including the revised text and representative revised figures. To facilitate the reviewers' assessment of the revision, the authors are encouraged to paste the key revised text directly into the response letter, rather than only providing page and line references. When major figure panels or supplementary figures have been added or substantially modified, representative images or cropped figure panels may also be included in the response letter. This would*

*allow reviewers to more clearly and efficiently evaluate how each comment has been addressed.*

**Response:** We appreciate this suggestion. We have directly included the key revised text passages in the relevant responses above to facilitate assessment of the revision.
